# Effect of Hypoglycemic Drugs on Patients with Heart Failure with or without T2DM: A Bayesian Network Meta-analysis

**DOI:** 10.31083/RCM26154

**Published:** 2025-03-21

**Authors:** Zhaolun Zhang, Siqi Liu, Jiawen Xian, Yali Zhang, Chunyu Zhang, Zhiyuan Wang, Hongmei Deng, Jian Feng, Lei Yao

**Affiliations:** ^1^Department of Cardiovascular Medicine, The Affiliated Hospital of Southwest Medical University, 646000 Luzhou, Sichuan, China; ^2^Department of Laboratory Medicine, The Affiliated Hospital of Southwest Medical University, 646000 Luzhou, Sichuan, China; ^3^Institute of Cardiovascular Research, Southwest Medical University, 646000 Luzhou, Sichuan, China; ^4^Provincial Key Laboratory for Human Disease Gene Study, Sichuan Academy of Medical Sciences and Sichuan Provincial People's Hospital, University of Electronic Science and Technology of China, 610072 Chengdu, Sichuan, China; ^5^The Center for Medical Genetics, Sichuan Academy of Medical Sciences and Sichuan Provincial People's Hospital, University of Electronic Science and Technology of China, 610072 Chengdu, Sichuan, China; ^6^Department of Laboratory Medicine, Sichuan Academy of Medical Sciences and Sichuan Provincial People's Hospital, University of Electronic Science and Technology of China, 610072 Chengdu, Sichuan, China; ^7^Research Unit for Blindness Prevention, Chinese Academy of Medical Sciences (2019RU026), Sichuan Academy of Medical Sciences and Sichuan Provincial People’s Hospital, 610072 Chengdu, Sichuan, China; ^8^Medical Experiment Center, The Affiliated Hospital of Southwest Medical University, 646000 Luzhou, Sichuan, China

**Keywords:** heart failure, hypoglycemic drugs, T2DM

## Abstract

**Background:**

Anti-diabetic drugs have been noted to have a cardioprotective effect in patients with diabetes and heart failure (HF). The purpose of this study was to perform a Bayesian network meta-analysis to evaluate the impact of various anti-diabetic drugs on the prognosis of HF patients with and without diabetes.

**Methods:**

We searched PubMed, Embase, Cochrane, and Web of Science for randomized controlled trials (RCTs) published before November 2024 that investigated the use of anti-diabetic medications in patients with HF. Primary outcomes included re-admission due to HF, all-cause death, cardiovascular death, serum N-terminal pro-brain natriuretic peptide (NTpro-BNP) levels, and left ventricular ejection fraction (LVEF). A Bayesian network meta-analysis was used to compare the effectiveness of different anti-diabetic drugs.

**Results:**

A total of 33 RCTs involving 29,888 patients were included. Sotagliflozin was the most effective in reducing the risk of re-admission due to HF and all-cause death, with a cumulative probability of 0.84 and 0.83, respectively. Liraglutide reduced the risk of cardiovascular death in HF patients with a cumulative probability of 0.97 and had the best efficacy in reducing NTpro-BNP levels with a cumulative probability of 0.69. Empagliflozin was best in improving LVEF in HF patients, with a cumulative probability of 0.69.

**Conclusions:**

This Bayesian network meta-analysis demonstrates that sotagliflozin may be the best option for HF patients with and without diabetes. However, due to the small number of articles in this study, our results must be treated cautiously. Subsequently, there is an urgent need for more high-quality studies to validate our findings.

## 1. Introduction 

Heart failure (HF) is one of the most severe cardiac conditions, characterized by an inability of the heart to efficiently pump blood to meet the demands of the body [1]. HF represents the predominant cause of cardiovascular-related hospitalizations in individuals aged over 60 and afflicts approximately 60 million patients globally [2, 3]. The prognosis for patients with HF is poor, with an average five-year survival rate of 46% [4, 5]. Type 2 diabetes mellitus (T2DM) manifests as elevated blood glucose levels in patients, stemming from a gradual reduction in insulin secretion by β-cells amidst insulin resistance [6]. Almost half of patients with T2DM also have HF, meaning HF is now considered an inevitable consequence of the cardiovascular progression of advanced T2DM [7, 8, 9]. Furthermore, both T2DM and HF independently increase the risk of other diseases [10, 11]. In the T2DM population, the prevalence of HF is greater than 40%, and the prevalence of T2DM in patients hospitalized with HF is also relatively high (around 20%) [11, 12, 13]. In patients with concurrent T2DM and HF, these two diseases adversely affect the prognosis of the other; meanwhile, the risk of hospitalization and death is significantly increased for HF patients [14, 15, 16]. Thus, implementing preventive measures and ensuring appropriate treatment for these two diseases is imperative.

Drug therapy has consistently served as the cornerstone of HF management. Diuretics positively impact patients’ prognoses by limiting fluid retention and alleviating cardiac work [17]. Angiotensin-converting enzyme (ACE) inhibitors, angiotensin receptor blockers, and β-blockers can positively influence cardiac remodeling during HF, thereby enhancing patients’ prognoses [18, 19, 20, 21, 22]. Simultaneously, numerous large-scale clinical randomized controlled trials (RCTs) have indicated that anti-diabetic drugs also exert a positive impact on the prognosis of HF patients [23, 24, 25, 26, 27, 28, 29, 30]. Several studies suggest that novel anti-diabetic drugs such as sodium–glucose cotransporter 2 (SGLT-2) inhibitors and glucagon-like peptide-1 (GLP1) receptor agonists (GLP1RAs) can enhance cardiovascular prognoses in patients with diabetes [31, 32, 33, 34, 35, 36, 37, 38]. GLP1RA can improve myocardial function by improving myocardial insulin resistance, promoting myocardial glucose uptake and oxidation [39], and increasing myocardial oxygen consumption and coronary blood flow [39, 40], as well as decreasing body weight [41], all of which promote the recovery of cardiac function. The cardiovascular benefits of GLP1RAs in non-T2DM patients were demonstrated in a large clinical trial, Semaglutide Effects on Cardiovascular Outcomes in People with Overweight or Obesity (SELECT) [42]. Applying an SGLT-2 inhibitor allows for a better prognosis by affecting glucose and sodium reabsorption in the proximal renal tubules [43, 44], improving cardiac workloads, and, consequently, improving ventricular remodeling [45]. SGLT-2 inhibitors also improve cardiac function in patients by reducing cardiomyocyte lipotoxicity [46], improving insulin resistance in the myocardium, and enhancing cardiac energy production [47]. The Dapagliflozin and Prevention of Adverse Outcomes in Heart Failure (DAPA-HF) trial [48] findings indicate that irrespective of the presence of T2DM, patients receiving an SGLT-2 inhibitor had a significantly lower risk of HF deterioration or cardiovascular death than the placebo group. This suggests that the therapeutic effects of SGLT-2 inhibitors in HF can be extended to patients without T2DM. The 2021 European Society of Cardiology (ESC) Guideline for the Management of Heart Failure [49] departs from the traditional Golden Triangle regimen but recommends four classes of drugs as the fundamental treatment for patients with HF with reduced ejection fraction (HFrEF) unless they are contraindicated or intolerant. These drugs include SGLT-2 inhibitors, ACE/angiotensin receptor–neprilysin inhibitors (ARNI), β-blockers, and mineralocorticoid receptor antagonists (MRAs). This treatment regimen is commonly referred to as a new quadruple therapy. The guidelines stipulate that, unless contraindicated or intolerant, all patients with HFrEF, regardless of diabetes status, should receive SGLT-2 inhibitors to mitigate the risk of hospitalization and death due to HF. In the latest 2023 ESC Guideline for the Management of Heart Failure [50], SGLT-2 inhibitors (dapagliflozin or empagliflozin) are recommended to diminish the risk of hospitalization or cardiovascular death (Class I, Level A) for patients with HF with mildly reduced ejection fraction (HFmrEF). The SGLT-2 inhibitors (dapagliflozin or empagliflozin) have become the first-line treatment with Class I recommendations for both HFmrEF and heart failure with preserved ejection fraction (HFpEF), as well as for reducing hospitalization or cardiovascular death due to HF. SGLT-2 inhibitors exhibit beneficial effects for HF patients with preserved ejection fraction.

Previous meta-analyses [51, 52, 53] have shown that SGLT-2 inhibitors and GLP1RAs exert positive effects on the prognosis of HF in both diabetic and non-diabetic patients. However, due to limited drug selections, a constrained research scope, and a scarcity of included articles, a more comprehensive discussion on the impact of anti-diabetic drugs on HF patients is lacking. A previous network meta-analysis (NMA) study [54] examined whether anti-diabetic drugs could enhance the prognosis of non-diabetic HF patients. However, this analysis had several limitations, including the exclusive focus on non-diabetic HF patients, a restricted database, a limited literature search, and incomplete results.

A network meta-analysis has many advantages over a traditional meta-analysis. Multiple drugs can be simultaneously compared, and direct and indirect evidence can be synthesized to compare drug efficacy. Clinical decision-making can incorporate various factors to provide a more comprehensive basis for individuals to choose the right drug. This is improved from the traditional method of comparing only two drugs. Therefore, this study aimed to conduct a network meta-analysis to compare the efficacy of different anti-diabetic drugs on re-admission rates, all-cause mortality, cardiovascular mortality, left ventricular ejection fraction (LVEF), and serum N-terminal pro-brain natriuretic peptide (NTpro-BNP) levels in heart failure patients with or without T2DM.

## 2. Materials and Methods

### 2.1 Retrieval Strategy

This network meta-analysis adhered to the Preferred Reporting Items for Systematic Reviews and Meta-Analyses (PRISMA) guidelines [55]. Up to November 11, 2024, we conducted searches on the PubMed, Embase, Web of Science, and Cochrane Library databases using the keywords: “Heart failure”, “anti-diabetic drugs”, and “RCT”. In addition, we manually searched the reference lists of the included studies and relevant reviews to identify any additional pertinent research. All search formulas are shown in **Supplementary Table 1**.

### 2.2 Exclusion and Inclusion Criteria

Studies that met the following criteria were included: (1) The diagnostic criteria for HF were 18 years or older and New York Heart Association functional class II–IV; other organic cardiac diseases were excluded. To minimize the impact of renal disease, participants were required to have an eGFR (estimated glomerular filtration rate) ≥30 mL/min/1.73 m^2^. The diagnostic criteria for HF for each of the included trials can be found in **Supplementary Table 2**. (2) The interventions involved anti-diabetic drugs, encompassing biguanides, sulfonylureas, thiazolidinediones, nateglinide, α-glucosidase inhibitors, dipeptidyl peptidase IV (DPP-4) inhibitors, SGLT-2 inhibitors, and new injectables (GLP1RAs). (3) The control group received a placebo or other anti-diabetic drugs. (4) Studies reported at least one of the outcome indicators of interest. The primary outcome indicators for this study were re-admission due to HF, all-cause death, and cardiovascular death. Secondary outcome indicators were changes in NTpro-BNP or LVEF levels. (5) The study design was a RCT. Excluded studies were observational studies, case–control studies, animal experiments, reviews, meta-analyses, editorials, comments, conference abstracts, and non-English articles.

### 2.3 Data Extraction

Two independent authors performed eligibility anonymized assessments for the included studies. The authors initially read titles and abstracts to exclude irrelevant studies and then downloaded and comprehensively reviewed the full text. A third author was consulted to resolve any disagreements.

Furthermore, two independent blinded authors extracted the following variables: (1) Patient baseline data, including age, region, and the presence of diabetes. (2) Pre-specified outcome indicators encompassing re-admission due to cardiovascular emergencies, cardiovascular death, all-cause death, and changes in LVEF or NTpro-BNP levels.

### 2.4 Risk of Bias

The quality of the included studies was assessed using the Cochrane Bias Risk Assessment Tool (RoB2.0, https://methods.cochrane.org/risk-bias-2) [56] in six domains: bias in randomization, bias from defined interventions, bias in missing outcome data, bias in outcome measurement, bias in selective reporting of results, and bias from other sources. Two investigators independently evaluated each study as “low risk”, “high risk”, and “possible risk” in the aforementioned six aspects. Any disagreements during the review process were resolved through discussion or consultation with a third researcher (if necessary).

### 2.5 Statistical Analysis

We chose the Bayesian network meta-analysis for the following reasons: (1) Bayesian methods can deal with uncertainty and complexity by providing a complete probability distribution of the parameter estimates through the posterior distribution, which helps to understand the uncertainty of the estimates more comprehensively. When dealing with complex models, Bayesian methods provide greater flexibility and are particularly suitable for dealing with multilevel data and nonlinear relationships. (2) The Bayesian methods allow us to incorporate prior information into the model, which can improve the accuracy of the estimates in some cases, especially when using small sample sizes or limited data.

We reported the mean after analyzing the posterior distribution. Dichotomous outcomes were displayed as the risk ratio (RR) with 95% credible intervals (CrI). Continuous variables outcomes were indicated as weighted mean differences (MDs) with 95% CrI. Given the heterogeneity between trials, the Bayesian hierarchical random effects model was first fitted for multiple comparisons of different disease treatment options [57, 58]. All the calculations and graphs were obtained using the R 4.2.1 (R Foundation for Statistical Computing, https://www.r-project.org) and Stata SE (StataCorp, College Station, TX, USA), version 15.1. We chose a non-informative, normal page distribution to minimize subjective effects. The specific parameters of the prior distribution were set as follows: mean 0 and variance 1. Based on the theory of likelihood function and some initial assumptions, the Markov chain Monte Carlo (MCMC) simulation was performed using Bayesian inference with R 4.2.1 software. In total, 500,000 were set for iterations and 20,000 for annealing to investigate the posterior distributions of the interrogated nodes [59, 60, 61]. The goodness-of-fit was assessed for the model fit by calculating the deviation information criterion (DIC). We used a random effects model to cope with the heterogeneity among the studies and reported the I^2^ statistic to quantify the extent of heterogeneity. We conducted subgroup and sensitivity analyses to explore further the sources of heterogeneity and their impact on our conclusions. The node-splitting method evaluated the local inconsistency for outcomes with closed loops. The relationships among the different treatments were presented as a network graph. A comparison-adjusted funnel plot was utilized to test for potential publication bias [62, 63]. We adopted surface under the cumulative ranking (SUCRA) probability values to rank the examined treatments, and the SUCRA values ranged from 0 to 1. A higher SUCRA value corresponds to a higher ranking than other treatments [64, 65]. The conjugate prior distribution was used for the Bayesian NMA. A league table was created to illustrate the comparisons between each pair of interventions for each outcome.

## 3. Results

### 3.1 Literature Search and Selection

**Fig. 1.**
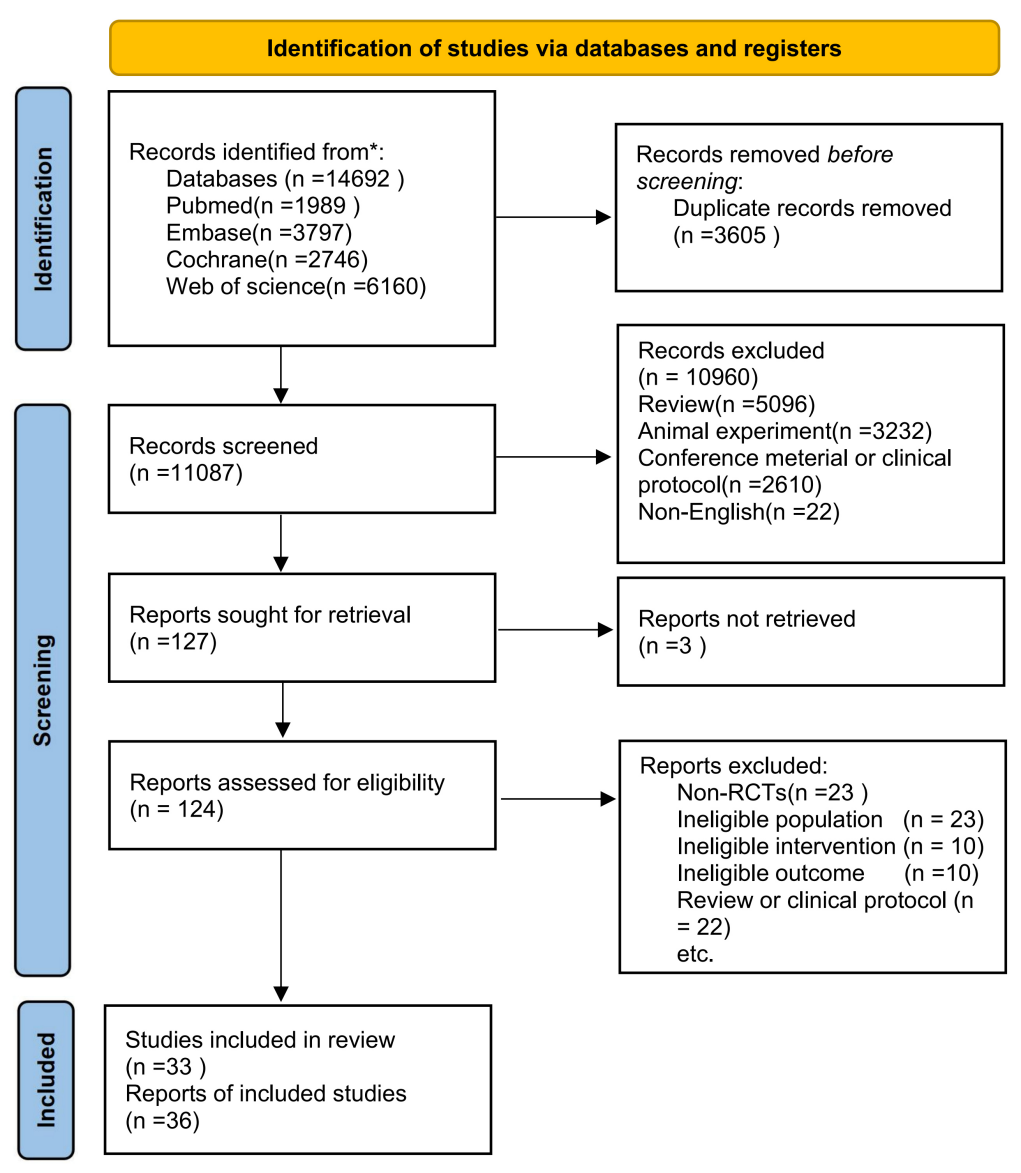
**Flow diagram of the Preferred Reporting Items for Systematic Reviews and Meta-Analyses (PRISMA) criteria**. RCTs, randomized controlled trials.

A total of 14,692 records were initially identified, and 11,087 records remained after deduplication. After screening titles and abstracts, 127 full-text articles were retrieved and assessed for eligibility. Ultimately, the analysis included 36 RCTs [23, 24, 25, 26, 31, 36, 48, 66, 67, 68, 69, 70, 71, 72, 73, 74, 75, 76, 77, 78, 79, 80, 81, 82, 83, 84, 85, 86, 87, 88, 89, 90, 91, 92, 93, 94], as depicted in Fig. 1. These studies encompassed 29,888 patients. The detailed baseline data of the included studies can be found in the **Supplementary Materials (Supplementary Table 2)**. The included studies were conducted in various countries, including China, Denmark, Italy, the United States, the United Kingdom, and Spain. All studies utilized a placebo control. All doses used in the experiments were therapeutic doses. Among the studies, 13 reported re-admission due to cardiovascular causes [23, 25, 26, 31, 48, 66, 67, 68, 69, 71, 74, 82, 83], 13 reported cardiovascular death [25, 26, 31, 48, 66, 67, 69, 70, 71, 73, 74, 75, 83], 12 reported all-cause death [25, 26, 31, 48, 66, 67, 69, 71, 73, 74, 75, 82], 15 reported LVEF [24, 26, 36, 69, 76, 77, 78, 79, 80, 81, 83, 88, 91, 92, 93], and 15 reported NTpro-BNP [23, 25, 48, 72, 75, 77, 79, 80, 81, 82, 87, 88, 89, 90, 94]. In total, 19 studies [23, 25, 36, 48, 66, 67, 74, 75, 76, 81, 82, 83, 87, 88, 89, 90, 91, 92, 94] included patients with or without T2DM, 13 included [31, 68, 69, 70, 71, 72, 73, 77, 79, 84, 85, 86, 93] patients with T2DM, and 4 included [24, 26, 78, 80] patients without T2DM. Giles *et al*. [84] compared the effects of pioglitazone and glyburide on patients, Carbone *et al*. [85] compared the effects of canagliflozin and sitagliptin, and Ejiri *et al*. [86] compared the effects of luseogliflozin and voglibose. Network analysis was not conducted as no closed loop was formed between interventions. **Supplementary Table 2** summarizes the baseline data for the included articles and the present Bayesian meta-analysis of pre-specified outcome metrics.

**Fig. 2.**
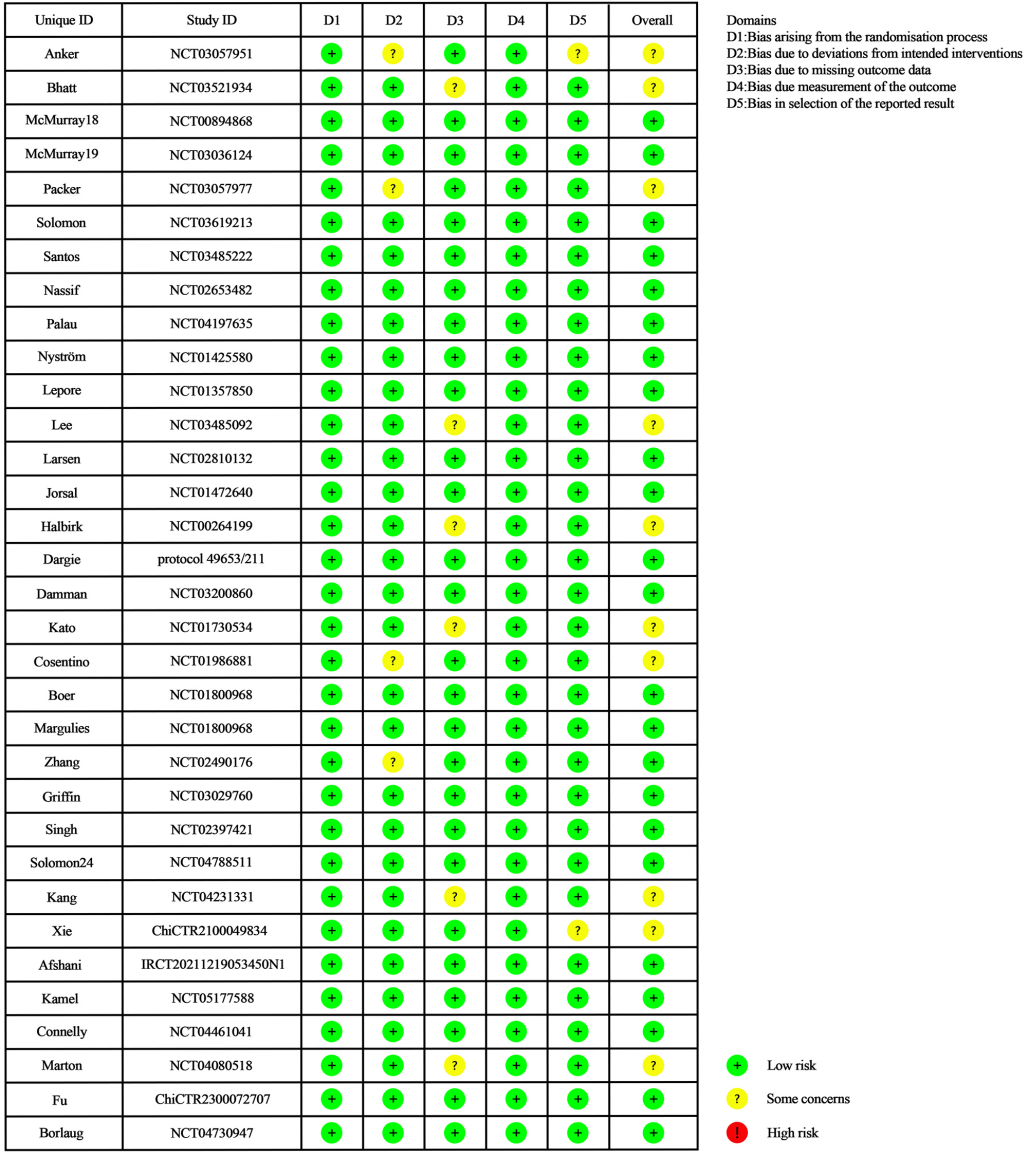
**Traffic light plot for the risk-of-bias assessment of included trials**.

Fig. 2 displays the quality assessment of the included studies. Overall, the risk of bias is notably low across all included studies. Additionally, the risk of bias in allocation concealment was low for all studies, except for Anker *et al*. [66] and Zhang *et al*. [83], which did not provide detailed descriptions of the allocation concealment process. Regarding participant blinding, all studies demonstrated a low risk, except those by Packer *et al*. [25] and Cosentino *et al*. [68], which lacked detailed descriptions of the blinding methods. In terms of reporting result data, all studies adequately reported their findings, except the studies by Bhatt *et al*. [67], Lee *et al*. [79], Kato *et al*. [71], Halbirk *et al*. [24] and Marton *et al*. [90], which exhibited some missing data. Moreover, all studies showed no potential bias risk regarding selective reporting, except for Anker *et al*. [66], Kang *et al*. [88], and Xie *et al*. [89].

### 3.2 Re-admission due to HF

**Fig. 3.**
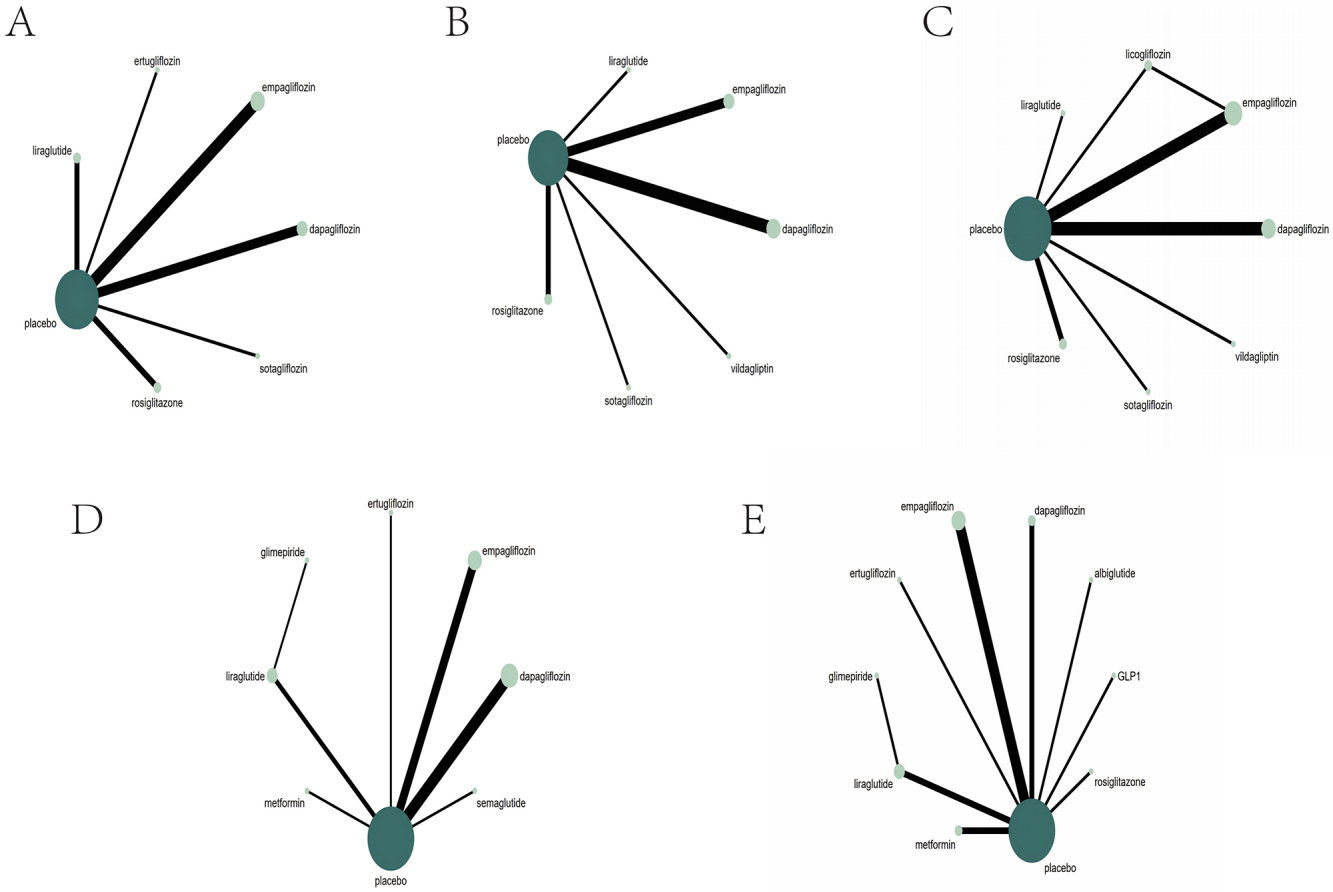
**The network graph for all outcomes**. (A) Re-admission due to HF; (B) all-cause death; (C) cardiovascular death; (D) NTpro-BNP; (E) LVEF. GLP1, glucagon-like peptide1; HF, heart failure; NTpro-BNP, N-terminal pro-brain natriuretic peptide; LVEF, left ventricular ejection fraction.

A total of 13 studies [23, 25, 26, 31, 48, 66, 67, 68, 69, 71, 74, 82, 83] involving 26,688 patients reported patient re-admissions due to HF. These studies encompassed six types of anti-diabetic drugs: dapagliflozin, empagliflozin, ertugliflozin, liraglutide, rosiglitazone, and sotagliflozin. All 13 studies were placebo-controlled trials. The network graph for re-admission due to HF is provided in Fig. 3A. In the diagram, the size of the dots represents the number of patients, and the thickness of the lines between interventions indicates the strength of the correlation between the two interventions.

According to the league table, in comparison to patients using liraglutide, those treated with dapagliflozin (RR = 0.62, 95% CrI (credible interval): (0.43, 0.94)), empagliflozin (RR = 0.61, 95% CrI: (0.40, 0.89)), ertugliflozin (RR = 0.56, 95% CrI: (0.33, 0.98)), and sotagliflozin (RR = 0.56, 95% CrI: (0.37, 0.89)) exhibited significantly lower re-admission rates.

Similarly, in comparison to patients using rosiglitazone, those treated with dapagliflozin (RR = 0.50, 95% CrI: (0.31, 0.81)), empagliflozin (RR = 0.49, 95% CrI: (0.29, 0.79)), ertugliflozin (RR = 0.45, 95% CrI: (0.25, 0.82)), and sotagliflozin (RR = 0.45, 95% CrI: (0.27, 0.76)) demonstrated significantly lower re-admission rates.

**Fig. 4.**
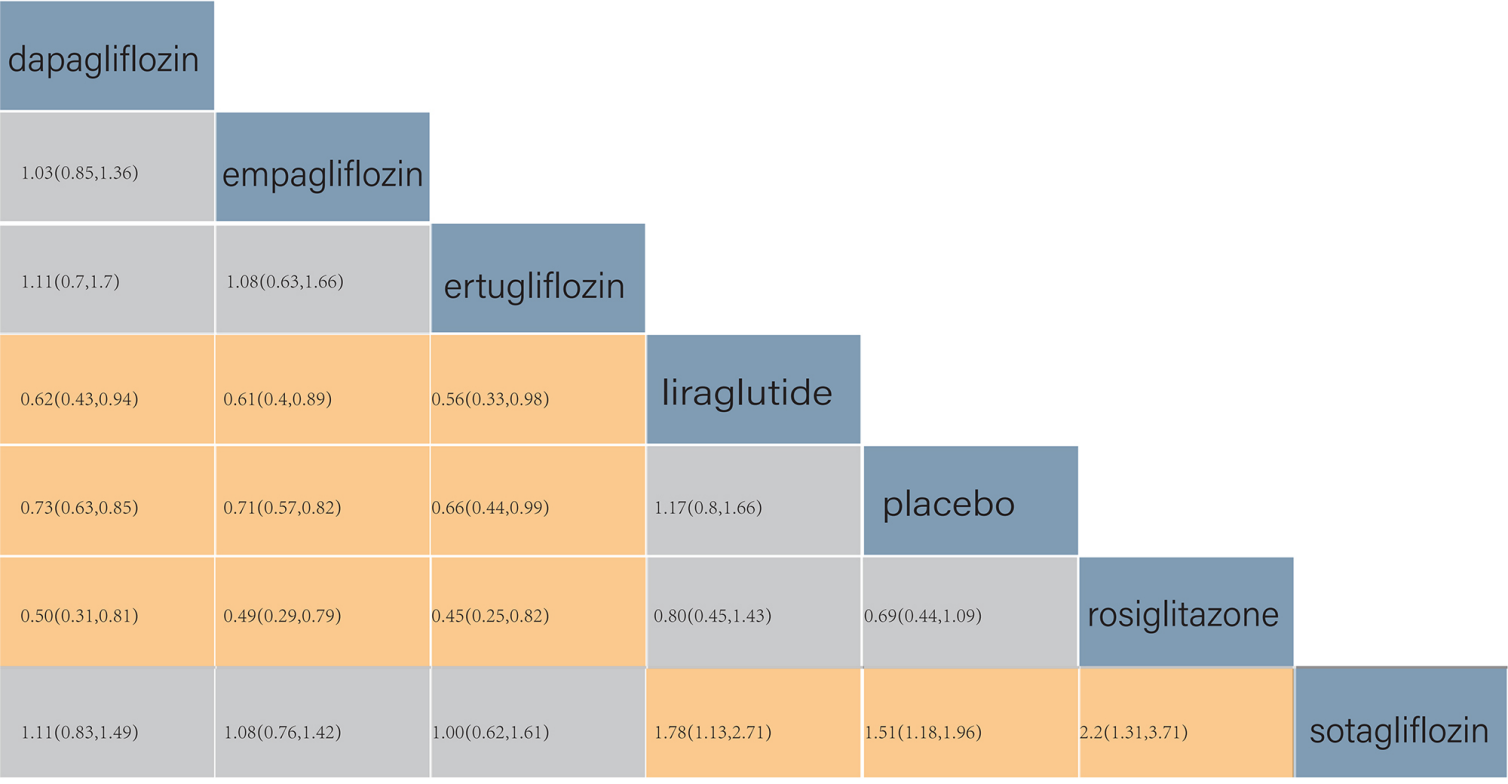
**Comparison of re-admission due to heart failure (HF)**. The values in each cell represent the relative treatment effect (95% credible intervals (CrI)) of the treatment on the right compared with the treatment on the top.

In comparison to patients using a placebo, those treated with dapagliflozin (RR = 0.73, 95% CrI: (0.63, 0.85)), empagliflozin (RR = 0.71, 95% CrI: (0.57, 0.82)), ertugliflozin (RR = 0.66, 95% CrI: (0.44, 0.99)), and sotagliflozin (RR = 0.66, 95% CrI: (0.51, 0.85)) exhibited significantly lower re-admission rates. A pairwise comparison of liraglutide, rosiglitazone, and placebo revealed no statistical difference in patient re-admission rates (Fig. 4).

**Fig. 5.**
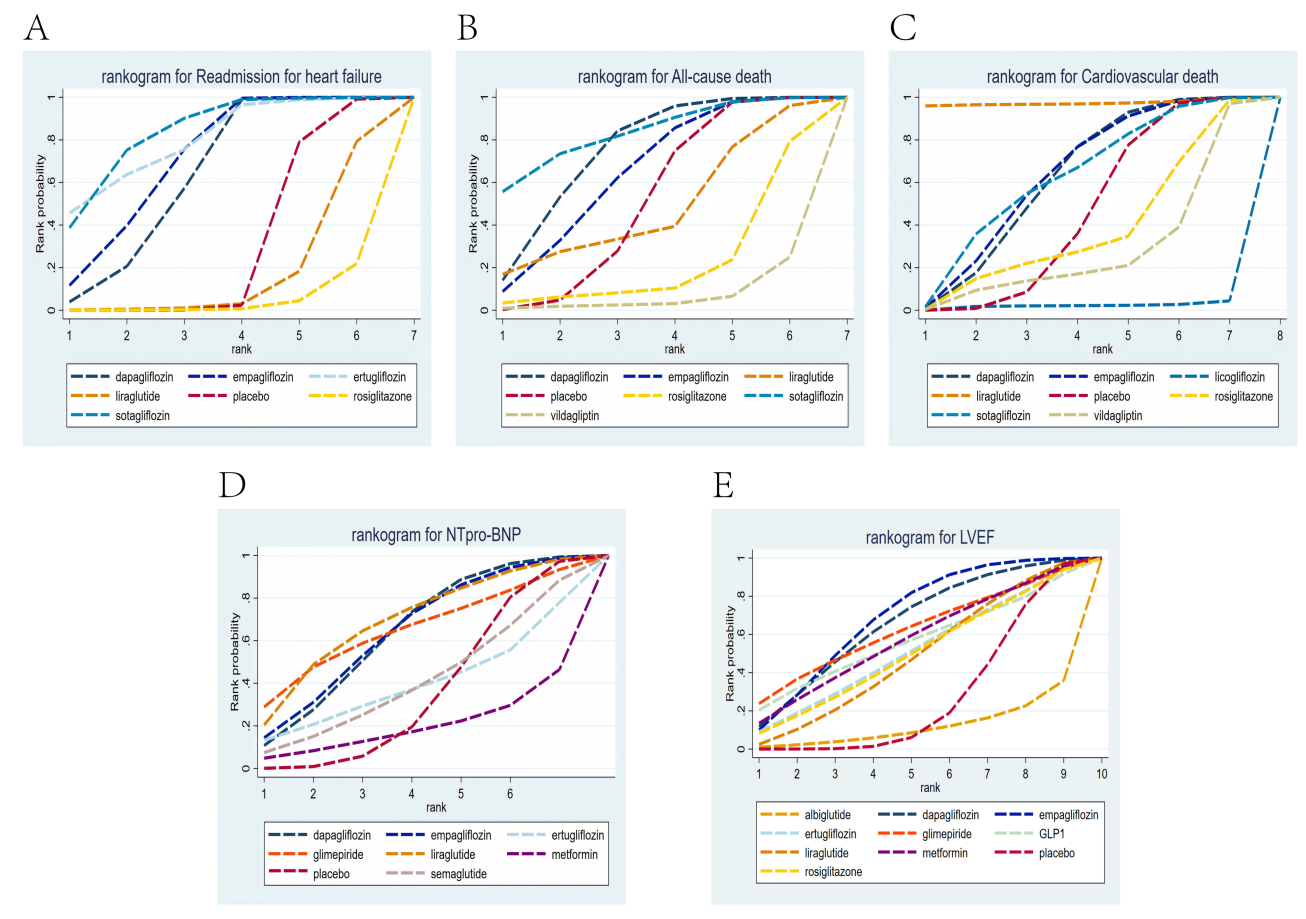
**Rankogram for all outcomes**. Each line segment represents a treatment. The area enclosed by the line segment and the coordinate axis represents the cumulative probability of treatment. (A) Readmission due to HF; (B) all-cause death; (C) cardiovascular death; (D) NTpro-BNP; (E) LVEF.

The ranking results revealed that sotagliflozin had the highest cumulative probability of efficacy in reducing patients’ re-admission due to HF, with a cumulative probability of 0.84. Ertugliflozin was second, with a cumulative probability of 0.79, followed by empagliflozin, with a cumulative probability of 0.71 (Fig. 5A).

### 3.3 All-cause Death

A total of 12 studies [25, 26, 31, 48, 66, 67, 69, 71, 73, 74, 75, 82] involving 25,115 patients reported all-cause deaths. These studies included six types of anti-diabetic drugs: dapagliflozin, empagliflozin, liraglutide, rosiglitazone, sotagliflozin, and vildagliptin. All 12 studies were placebo-controlled trials, and the network graph for all-cause death is provided in Fig. 3B.

**Fig. 6.**
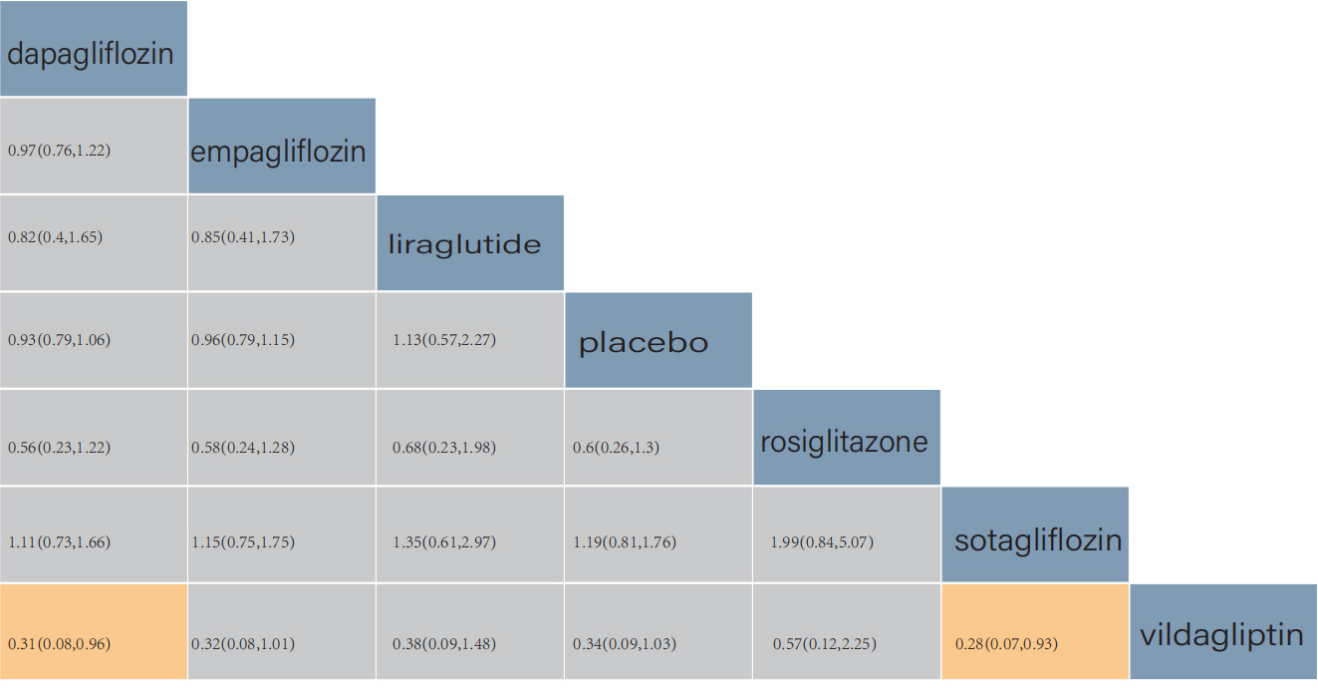
**Comparison of all-cause death**. The values in each cell of the league table heatmap represent the relative treatment effect (95% CrI) of the treatment on the right compared with the treatment on the top.

According to the league table, in comparison with patients using vildagliptin, those treated with dapagliflozin (RR = 0.32, 95% CrI: (0.09, 0.98)) or sotagliflozin (RR = 0.29, 95% CrI: (0.07, 0.93)) exhibited a lower all-cause mortality rate. The pairwise comparison of other drugs showed no statistical difference in patient cardiovascular death (Fig. 6).

The ranking results revealed that sotagliflozin ranked first in the cumulative probability of efficacy for reducing all-cause patient death, with a cumulative probability of 0.83. Dapagliflozin ranked second, with a cumulative probability of 0.74, followed by empagliflozin, with a cumulative probability of 0.64 (Fig. 5B).

### 3.4 Cardiovascular Death

A total of 13 studies [25, 26, 31, 48, 66, 67, 69, 70, 71, 73, 74, 75, 83] involving 24,992 patients reported cardiovascular death. These studies included seven types of anti-diabetic drugs: dapagliflozin, empagliflozin, licogliflozin, liraglutide, rosiglitazone, sotagliflozin, and vildagliptin. All 13 studies were placebo-controlled trials. The network graph for cardiovascular death is provided in Fig. 3C.

**Fig. 7.**
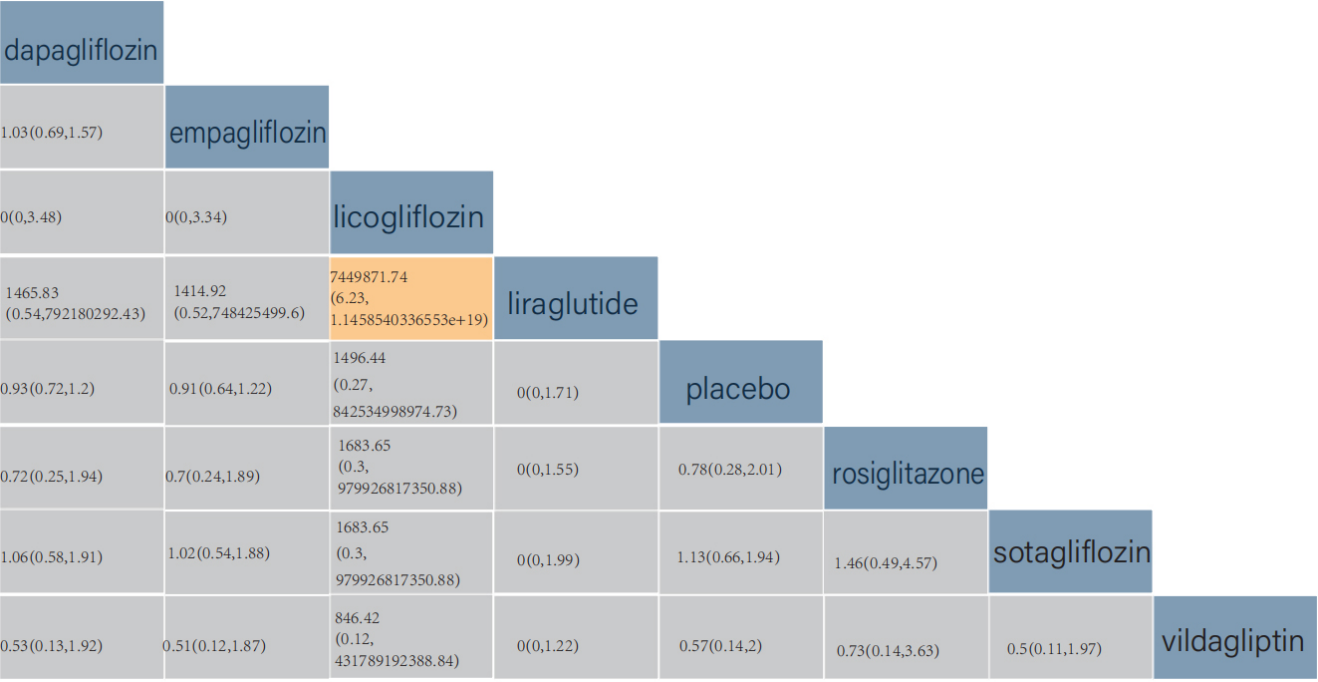
**Comparison of cardiovascular death**. The values in each cell of the league table heatmap represent the relative treatment effect (95% CrI) of the treatment on the right compared with the treatment on the top.

According to the league table, when compared with licogliflozin, liraglutide (RR = 7,449,871.74, 95% CrI: (6.23, 1.1458540336553 × 10^19^)) was more effective in reducing cardiovascular mortality in patients. The pairwise comparison of other drugs revealed no statistical difference in cardiovascular death (Fig. 7).

The ranking results revealed that liraglutide had the highest cumulative probability of efficacy for reducing cardiovascular death, at 0.97. Empagliflozin was second, with a cumulative probability of 0.63, followed by sotagliflozin, with a cumulative probability of 0.62 (Fig. 5C).

### 3.5 NTpro-BNP

The changes in NTpro-BNP were discussed in 15 studies [23, 25, 72, 73, 75, 77, 79, 80, 81, 82, 87, 88, 89, 90, 94] involving 10,374 patients. These studies encompassed dapagliflozin, empagliflozin, glimepiride, liraglutide, ertugliflozin, semaglutide and metformin. All 15 studies were placebo-controlled trials. The network graph for the changes in NTpro-BNP is provided in Fig. 3D.

**Fig. 8.**
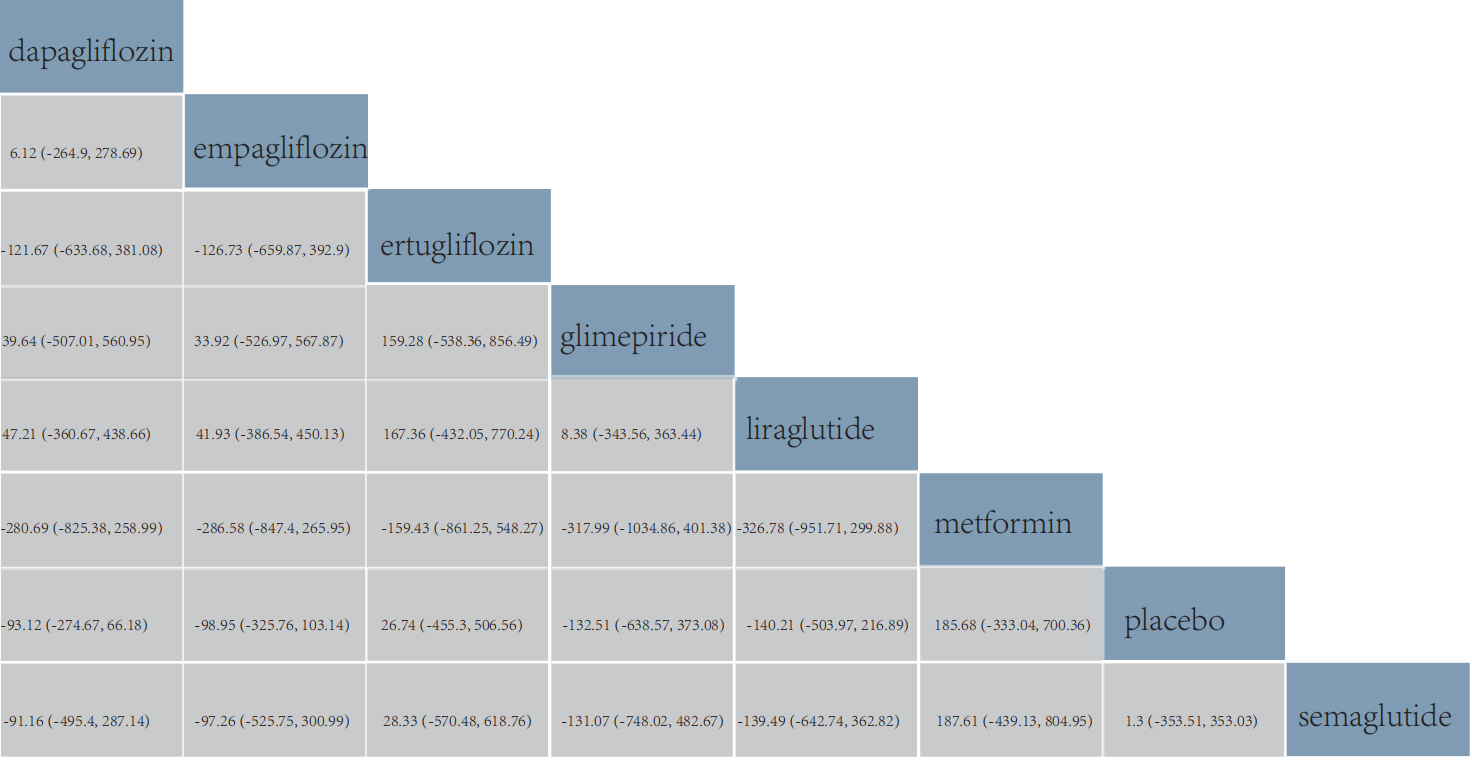
**Comparison of changes in serum NTpro-BNP levels**. The values in each cell of the league table heatmap represent the relative treatment effect (95% CrI) of the treatment on the right compared with the treatment on the top.

According to the league table, the pairwise comparison of all six drugs revealed no significant differences in the NTpro-BNP levels (Fig. 8).

The ranking results revealed that liraglutide ranked first in the cumulative probability of changes in NTpro-BNP levels, with a cumulative probability of 0.69. Glimepiride was second, with a cumulative probability of 0.65, followed by empagliflozin, with a cumulative probability of 0.64 (Fig. 5D).

### 3.6 LVEF

**Fig. 9.**
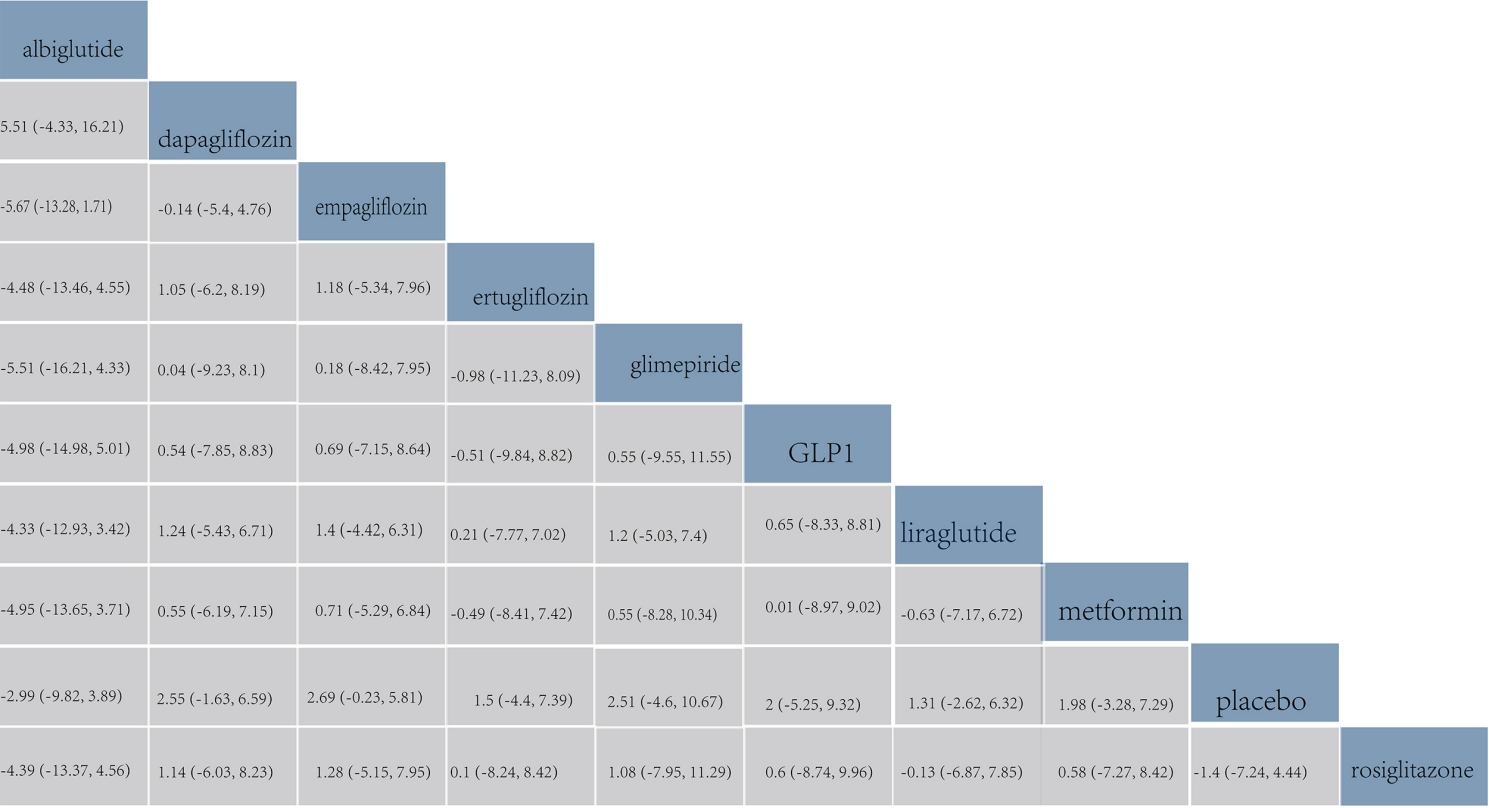
**Comparison of changes in left ventricular ejection fraction**. The values in each cell of the league table heatmap represent the relative treatment effect (95% CrI) of the treatment on the right compared with the treatment on the top.

A total of 15 studies [24, 26, 36, 69, 76, 77, 78, 79, 80, 81, 83, 88, 91, 92, 93] involving 1736 patients reported changes in LVEF. These studies included nine anti-diabetic drugs: albiglutide, dapagliflozin, empagliflozin, ertugliflozin, glimepiride, native GLP1, liraglutide, metformin, and rosiglitazone. All 15 studies were placebo-controlled trials. The network graph illustrating changes in LVEF is shown in Fig. 3E. Furthermore, according to the league table, the pairwise comparison of all eight drugs showed no statistical differences in changes in LVEF (Fig. 9).

The ranking results showed that empagliflozin ranked first in the cumulative probability of efficacy for changes in LVEF, with a cumulative probability of 0.69; dapagliflozin was second, with a cumulative probability of 0.65, and glimepiride was third, with a cumulative probability of 0.62 (Fig. 5E).

### 3.7 Subgroup Analysis

To minimize the effect of heterogeneity, we conducted subgroup analyses of heart failure patients with or without HFrEF at baseline status, with or without T2DM, and with a follow-up time of greater than or less than one year to observe the treatment effect and performed sequencing for the different subgroups.

#### 3.7.1 HErEF Subgroup

3.7.1.1 Re-admission due to HF

**Fig. 10.**
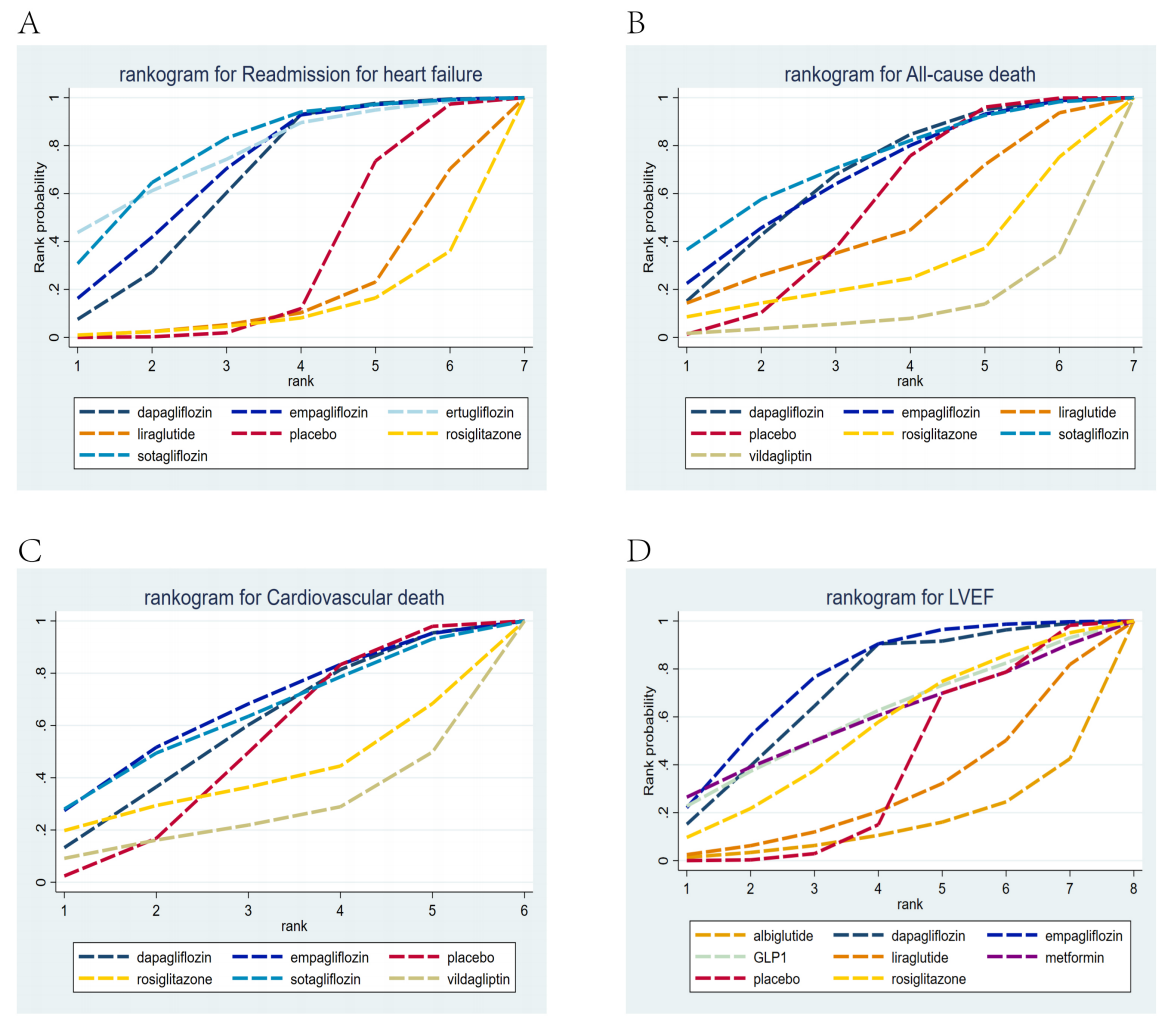
**Rankogram for all outcomes of the subgroup with HErEF**. Each line segment represents a treatment. The area enclosed by the line segment and the coordinate axis represents the cumulative probability of treatment. (A) Readmission due to HF; (B) all-cause death; (C) cardiovascular death; (D) LVEF. HErEF, HF with reduced ejection fraction.

A subgroup analysis was conducted based on the baseline LVEF. A total of eight studies [25, 26, 48, 67, 68, 69, 71, 82] involving 12,769 patients reported re-admission rates due to HF in patients with HErEF. These studies involved six anti-diabetic drugs: dapagliflozin, empagliflozin, ertugliflozin, liraglutide, rosiglitazone, and sotagliflozin. All eight studies were placebo-controlled trials. The network graph for the re-admission of HErEF patients due to HF is provided in **Supplementary Fig. 1**. According to the league table, no significant differences were observed in re-admission rates in HErEF patients after administering any of the six anti-diabetic drugs. The details are presented in **Supplementary Table 3**. Sotagliflozin ranked first in the cumulative probability of efficacy for reducing re-admission due to HF in patients, with a cumulative probability of 0.78. Empagliflozin ranked second, with a cumulative probability of 0.69, followed by dapagliflozin, with a cumulative probability of 0.64 (Fig. 10A).

3.7.1.2 All-cause Death

A subgroup analysis was conducted based on the baseline LVEF data. A total of nine studies [25, 26, 48, 67, 69, 71, 73, 75, 82] involving 12,808 patients reported all-cause mortality in HErEF patients. These studies involved six anti-diabetic drugs: dapagliflozin, empagliflozin, liraglutide, rosiglitazone, sotagliflozin, and vildagliptin. All nine studies were RCTs. The network graph illustrating all-cause mortality in HErEF patients is provided in **Supplementary Fig. 2**. According to the league table, no statistical differences were found in all-cause mortality in HErEF patients among the six drugs. Details are illustrated in **Supplementary Table 4**.

The drug ranking indicated that sotagliflozin ranked first in the cumulative probability of efficacy for reducing all-cause mortality in HErEF patients, with a cumulative probability of 0.73. Dapagliflozin ranked second, with a cumulative probability of 0.68, followed by empagliflozin, with a cumulative probability of 0.67 (Fig. 10B).

3.7.1.3 Cardiovascular Death

Similarly, a subgroup analysis was conducted based on the baseline LVEF data. A total of eight studies [25, 26, 48, 67, 69, 71, 73, 75] involving 12,508 patients reported cardiovascular mortality in HErEF patients. These studies involved five anti-diabetic drugs: dapagliflozin, empagliflozin, rosiglitazone, sotagliflozin, and vildagliptin. All eight studies were RCTs. The network graph illustrating cardiovascular mortality in HErEF patients is provided in **Supplementary Fig. 3**. According to the league table, the effects of all five drugs and placebo on all-cause mortality of HErEF patients showed no statistical differences. The details are illustrated in **Supplementary Table 5**. The drug ranking revealed that empagliflozin ranked first in the cumulative probability of efficacy for reducing cardiovascular mortality, with a cumulative probability of 0.65. Sotagliflozin ranked second, with a cumulative probability of 0.62, followed by dapagliflozin (Fig. 10C).

3.7.1.4 LVEF

Subgroup analysis was conducted based on the baseline LVEF. A total of ten studies [24, 26, 69, 76, 78, 79, 80, 81, 91, 93] involving 1046 patients reported changes in LVEF in HErEF patients. These studies involved seven anti-diabetic drugs: albiglutide, dapagliflozin, empagliflozin, GLP1, liraglutide, metformin, and rosiglitazone. All ten studies were RCTs. The network graph illustrating the changes in LVEF for HErEF patients is depicted in **Supplementary Fig. 4**. According to the league table, the effects of all seven drugs and placebo on the changes in LVEF of HErEF patients showed no statistical differences. Details are illustrated in **Supplementary Table 6**. The drug ranking showed that empagliflozin ranked first in the cumulative probability of efficacy for changes in LVEF, with a cumulative probability of 0.76. Dapagliflozin ranked second, with a cumulative probability of 0.69, followed by GLP1, with a cumulative probability of 0.60 (Fig. 10D).

#### 3.7.2 T2DM Subgroup

3.7.2.1 Re-admission due to HF

**Fig. 11.**
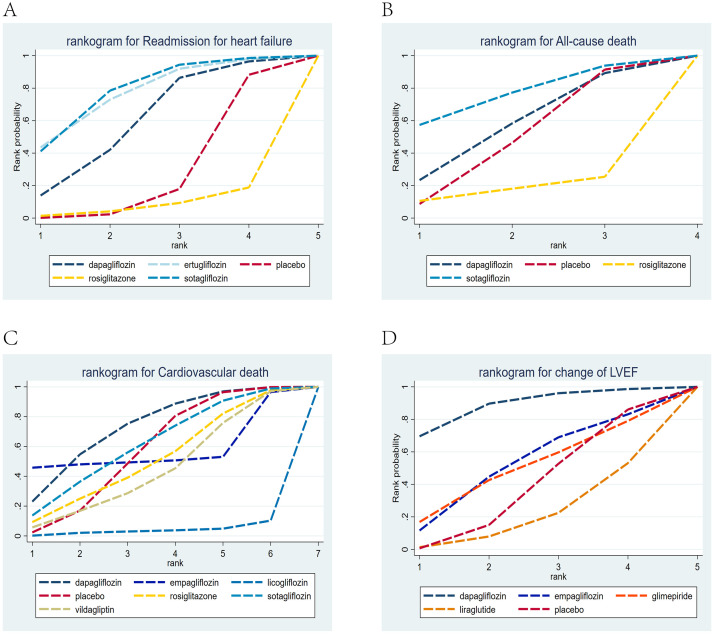
**Rankogram for all subgroup outcomes with type 2 diabetes mellitus (T2DM)**. Each line segment represents a treatment. The area enclosed by the line segment and the coordinate axis represents the cumulative probability of treatment. (A) Readmission due to HF; (B) all-cause death; (C) cardiovascular death; (D) LVEF.

Subgroup analysis was conducted for re-admission due to HF based on whether patients had T2DM. A total of five studies [31, 67, 68, 69, 71] involving 5447 patients reported re-admission rates due to HF in patients with HF and T2DM. These studies involved four anti-diabetic drugs: sotagliflozin, rosiglitazone, dapagliflozin, and ertugliflozin. All five studies were RCTs. The network graph of the re-admission rates for HF patients with T2DM is specified in **Supplementary Fig. 5**. According to the league table, the effects of all four drugs and placebo on the re-admission rates due to HF in patients with HF and T2DM showed no statistical differences. Details are illustrated in **Supplementary Table 7**. The drug ranking indicated that sotagliflozin ranked first in reducing the HF-related re-admission rates of patients, with a cumulative probability of 0.78. Ertugliflozin ranked second, with a cumulative probability of 0.76, followed by dapagliflozin, with a cumulative probability of 0.60 (Fig. 11A).

3.7.2.2 All-cause Death

A subgroup analysis was conducted for all-cause death based on whether patients had T2DM. A total of four studies [31, 67, 69, 71] involving 3489 patients reported all-cause mortality in HF patients with T2DM. These studies involved three anti-diabetic drugs: dapagliflozin, rosiglitazone, and sotagliflozin. All four studies were RCTs, and the network graph of all-cause mortality of HF patients with T2DM is specified in **Supplementary Fig. 6**. According to the league table, all three drugs and placebo showed no statistical differences in the all-cause mortality of HF patients with T2DM. Details are illustrated in **Supplementary Table 8**.

The drug ranking revealed that sotagliflozin ranked first in reducing all-cause mortality, with a cumulative probability of 0.76. Dapagliflozin ranked second, with a cumulative probability of 0.57, followed by placebo, with a cumulative probability of 0.49, as shown in Fig. 11B.

3.7.2.3 Cardiovascular Death

A subgroup analysis was conducted for cardiovascular death based on whether patients had T2DM. A total of six studies [31, 67, 69, 70, 71, 73] involving 3868 patients reported re-admission rates due to HF in HF patients with T2DM. These studies involved six anti-diabetic drugs: dapagliflozin, empagliflozin, licogliflozin, rosiglitazone, sotagliflozin, and vildagliptin. All six studies were RCTs. The network graph of cardiovascular mortality of HF patients with T2DM is specified in **Supplementary Fig. 7**.

According to the league table, the effects of all six drugs and placebo on the cardiovascular mortality for HF patients with T2DM were comparable. Details are illustrated in **Supplementary Table 9**. The drug ranking revealed that dapagliflozin ranked first in reducing cardiovascular mortality, with a cumulative probability of 0.73. Sotagliflozin ranked second, with a cumulative probability of 0.61, followed by empagliflozin, with a cumulative probability of 0.54 (Fig. 11C).

3.7.2.4 LVEF

A subgroup analysis was conducted based on the baseline LVEF in patients with T2DM. A total of five studies [31, 77, 79, 81, 93] involving 524 patients reported changes in LVEF in HF patients with T2DM. These studies involved four anti-diabetic drugs: dapagliflozin, empagliflozin, glimepiride, and liraglutide. All five studies were RCTs. The network graph of changes in LVEF in patients with HF and T2DM is specified in **Supplementary Fig. 8**. According to the league table, the effects of all four drugs and placebo on the changes in LVEF for HF patients with T2DM had no statistical differences. Details are illustrated in **Supplementary Table 10**.

The drug ranking showed that dapagliflozin ranked first in the cumulative probability of efficacy for changes in LVEF, with a cumulative probability of 0.88. Empagliflozin ranked second, with a cumulative probability of 0.52, followed by glimepiride, with a cumulative probability of 0.49 (Fig. 11D).

#### 3.7.3 Non-T2DM Subgroup

LVEF

A subgroup analysis was conducted based on the baseline LVEF of patients. A total of four studies [24, 26, 78, 80] involving 222 patients reported changes in LVEF in HF patients without T2DM. These studies involved four anti-diabetic drugs: albiglutide, empagliflozin, GLP1, and metformin. All four studies were RCTs. The network graph of changes in LVEF in HF patients without T2DM is specified in **Supplementary Fig. 9**. According to the league table, the effects of all four drugs and placebo on the changes in LVEF for HF patients without T2DM exhibited no statistical differences. Details are illustrated in **Supplementary Table 11**. The drug ranking suggested that empagliflozin ranked first in the cumulative probability of efficacy for changes in LVEF, with a cumulative probability of 0.86. GLP1 ranked second, with a cumulative probability of 0.56, followed by metformin, with a cumulative probability of 0.55, as specified in **Supplementary Fig. 10**.

#### 3.7.4 More than One Year Subgroup

Due to significant differences in primary outcome indicators in follow-up times, we conducted subgroup analyses based on whether the follow-up was over one year. Two secondary outcomes were used for subgroup analyses: all included trials had less than one year of follow-up, and no long-term follow-up was allowed.

3.7.4.1 Re-admission due to HF

A total of nine studies [25, 31, 48, 66, 67, 68, 69, 71, 74] were conducted, involving 26,172 patients, to assess the impact of five different anti-diabetic drugs (dapagliflozin, empagliflozin, ertugliflozin, rosiglitazone, sotagliflozin) on re-admission due to HF with a follow-up time of more than one year. These studies were all placebo-controlled trials. The network graph depicting re-admission due to HF with a follow-up time exceeding one year can be found in **Supplementary Fig. 11**. Based on the league table, those treated with dapagliflozin (RR = 0.73, 95% CrI: 0.464, 0.84), empagliflozin (RR = 0.73, 95% CrI: 61, 0.86), ertugliflozin (RR = 0.66, 95% CrI: 0.45, 0.98), and sotagliflozin (RR = 0.66, 95% CrI: 0.52, 0.83) exhibited significantly lower re-admission rates than patients using the placebo.

Similarly, in comparison to patients using rosiglitazone, those treated with dapagliflozin (RR = 0.50, 95% CrI: (0.25, 0.96), empagliflozin (RR = 0.50, 95% CrI: (0.25, 0.96)), ertugliflozin (RR = 0.45, 95% CrI: (0.21, 0.96)), and sotagliflozin (RR = 0.45, 95% CrI: (0.22, 0.89)) demonstrated significantly lower re-admission rates. Further details can be found in **Supplementary Table 12**. In evaluating these five drugs, sotagliflozin emerged as the top-ranking drug in terms of cumulative probability of efficacy for cardiovascular mortality, with a cumulative probability of 0.82. Ertugliflozin followed closely behind with a cumulative probability of 0.76, and empagliflozin ranked third with a cumulative probability of 0.60. More information can be found in **Supplementary Fig. 12**.

3.7.4.2 All-cause Death

A total of ten studies [25, 26, 31, 48, 66, 67, 69, 71, 73, 74] involving 24,552 patients assessed the impact of five different anti-diabetic drugs (dapagliflozin, empagliflozin, vildagliptin, rosiglitazone, sotagliflozin) on all-cause death with a follow-up time of more than one year. All ten studies were RCTs, and the network graph of all-cause mortality with a follow-up time exceeding one year is specified in **Supplementary Fig. 13**. According to the league table, all five drugs and placebo showed no statistical differences in the all-cause mortality in HF patients with T2DM. Details are illustrated in **Supplementary Table 13**.

The drug ranking revealed that sotagliflozin ranked first in reducing all-cause mortality, with a cumulative probability of 0.83. Dapagliflozin ranked second, with a cumulative probability of 0.73, followed by placebo, with a cumulative probability of 0.62; more information can be found in **Supplementary Fig. 14**.

3.7.4.3 Cardiovascular Death

A total of nine studies [25, 31, 48, 66, 67, 69, 71, 73, 74] were conducted, involving 24,468 patients, to assess the impact of six different anti-diabetic drugs (dapagliflozin, empagliflozin, rosiglitazone, sotagliflozin, vildagliptin, and placebo) on cardiovascular death with a follow-up time of more than one year. These studies were all placebo-controlled trials. The network graph depicting cardiovascular deaths with a follow-up time exceeding one year can be found in **Supplementary Fig. 15**. Based on the league table, there was no statistically significant difference in the effects of all five drugs and placebo on cardiovascular mortality for HF patients after pairwise comparison. Further details can be found in **Supplementary Table 14**. In evaluating these six drugs, dapagliflozin emerged as the top-ranking drug in terms of cumulative probability of efficacy for cardiovascular mortality, with a cumulative probability of 0.67. Empagliflozin followed closely behind with a cumulative probability of 0.66, and sotagliflozin ranked third with a cumulative probability of 0.65. More information can be found in **Supplementary Fig. 16**.

#### 3.7.5 Less than One Year

Cardiovascular Death

A total of four studies [26, 70, 75, 83] were conducted involving 524 patients to evaluate the outcome indicator of cardiovascular death over a follow-up period of more than one year. The studies included four different types of anti-diabetic drugs: dapagliflozin, empagliflozin, licogliflozin, and liraglutide. All four studies were placebo-controlled trials. The network graph illustrating cardiovascular deaths with a follow-up time of less than one year can be found in **Supplementary Fig. 17**. According to the league table, empagliflozin demonstrated a more significant decrease in cardiovascular mortality among patients (RR = 0.00, 95% CrI: (0.00, 0.27)) compared to licogliflozin. However, no statistically significant effects were observed on cardiovascular mortality for HF patients in the pairwise comparison of other drugs and placebo. Further details can be found in **Supplementary Table 15**. In the evaluation of five drugs, dapagliflozin, empagliflozin, licogliflozin, liraglutide, and placebo, the drug ranking first in terms of cumulative probability of efficacy for cardiovascular mortality was liraglutide, with a cumulative probability of 0.86. Empagliflozin ranks second with a cumulative probability of 0.80, followed by dapagliflozin in third place with a cumulative probability of 0.39. Additional information can be found in **Supplementary Fig. 18**.

### 3.8 Publication Bias and Diagnosis of Convergence

Funnel plots were employed to assess publication bias for all outcome indicators. The funnel plot is viewed through a scatter distribution. Ideally, if no publication bias exists, the funnel plot should be symmetrical, with studies with small sample sizes scattered at the bottom and studies with large samples concentrated at the top. The funnel plots for five outcome indicators were asymmetrical, implying the potential presence of publication bias (**Supplementary Figs. 19–36**).

### 3.9 Diagnosis of Convergence and Heterogeneity Analysis

We ensured adequate convergence of the model by running multiple Markov chains and checking the Gelman-Rubin statistic (R^R^); a potential scale reduction factor (PSRF) close to 1 indicates model convergence. Detailed convergence diagnostic results, including chain trace plots to demonstrate the stability of the model parameter estimates, are presented in the **Supplementary Material (Supplementary Figs. 37–54)**.

We used a random effects model to assess the heterogeneity between studies and reported the I^2^ statistic to quantify the degree of heterogeneity. All results can be found in the **Supplementary Material (Supplementary Figs. 55–78)**.

## 4. Discussion

To our knowledge, this represents the first NMA comparing the effectiveness and safety of various anti-diabetic drugs in treating patients with HF. This NMA scrutinized the most recent data from 33 eligible RCTs. Our findings indicate that administering sotagliflozin is the most productive strategy for reducing re-admission rates and all-cause mortality in patients with HF. Liraglutide treatment emerged as the most effective approach for mitigating cardiovascular mortality. Administering liraglutide also allows patients to optimize their NTpro-BNP levels, while empagliflozin is the optimal choice for enhancing LVEF in patients. Notably, there were no significant differences among the various anti-diabetic drugs.

GLP1RAs and SGLT-2 inhibitors stand out as primary choices for treating T2DM, and their benefits in cardiovascular diseases have been progressively demonstrated in recent years [95]. GLP1 constitutes a principal physiological incretin effect within the body. Incretin, an intestine-derived peptide of the glucagon superfamily, is secreted by the small intestine and increases the insulin secretion response to nutrients by binding to specific receptors expressed in pancreatic β-cells [96]. The primary function of GLP1 involves regulating systemic blood glucose levels [97]. Additionally, GLP1 enhances the expression of antioxidant enzymes and activates the nuclear factor erythroid 2-related factor 2–antioxidant response element (Nrf2–ARE) signaling pathway [98], thereby reducing reactive oxygen species (ROS) levels and protecting the heart from oxidative stress. Furthermore, GLP1 inhibits the mitogen-activated protein kinase kinase 4 (MKK 4)/mitogen-activated protein kinase kinase 7 (MKK 7)/c-Jun N-terminal kinase (JNK) cascade in the signal transduction pathway, mediating cell apoptosis by enhancing PKA activity. This inhibition results in a reduction in oxidative stress-induced apoptosis in cardiac progenitor cells [99]. GLP1 also regulates cardiac function through various indirect mechanisms, such as inhibiting the renin–angiotensin system to lower blood pressure and regulate myocardial mitochondrial function [100], altering substrate delivery of fatty acids and glucose to the heart [11], suppressing inflammation by regulating the expression of inflammatory genes in peripheral blood mononuclear cells [101], and reducing body weight by regulating adipocyte development and stimulating thermogenesis in brown adipose tissue (BAT) [100, 102]. In our study, liraglutide emerged as the most effective intervention for improving cardiovascular mortality and reducing the NTpro-BNP levels in patients. Liraglutide, a long-acting GLP1RA, is widely employed in clinical practice [103]. Furthermore, the potential impact of liraglutide on weight control [104, 105] may significantly contribute to the observed improvement in cardiovascular mortality. Previous studies have demonstrated that liraglutide is significantly more effective than a placebo in reducing fasting weight and waist circumference and enhancing certain biomarkers of cardiovascular risk [106, 107, 108]. Recent studies have shown that liraglutide improves BNP levels and cardiac diastolic function in obese patients but has a negligible effect on LVEF [82, 109, 110], which may explain the ineffectiveness of liraglutide in improving re-admission and all-cause mortality rates in patients. A decrease in LVEF further increases the cardiac burden of the patient and increases the risk of complications. For overweight patients with heart failure with preserved ejection fraction, liraglutide can be used to enhance their quality of life. However, it is important to exercise caution when interpreting this conclusion due to the limited number of patients with follow-up times of less than one year and the infrequent occurrence of the outcome indicator of cardiovascular mortality. The overall low incidence of cardiovascular mortality events may have resulted in a confidence interval that is too wide. To address this, we have categorized the articles incorporating this indicator into two subgroups based on the follow-up duration. One subgroup consists of articles with a follow-up time exceeding one year, while the other comprises articles with less than one year. These distinct subgroups yielded significant outcome variations due to studies and medication discrepancies. In the subgroup with a substantial sample size of follow-up times exceeding one year, dapagliflozin emerged as the most effective drug in reducing cardiovascular mortality in HF patients. In contrast, in the subgroup with a limited sample size and follow-up times of less than one year, liraglutide exhibited superiority in improving cardiovascular mortality in HF patients. In addition, there are some limitations in reducing NTpro-BNP, another indicator that showed the greatest efficiency. For example, it is well known that age and the method of measurement can greatly influence the measurement of NTpro-BNP [111, 112], and the variability of the sensitivity and specificity of the assay may also influence the diagnosis and treatment of clinically relevant cardiac disorders [113, 114]. However, the measurement method for NTpro-BNP was not accurately described since NTpro-BNP appears as a secondary outcome indicator in most studies. Therefore, we could not perform subgroup analyses to obtain more reliable results. Unfortunately, there is a dearth of studies that specifically investigate the impact of using liraglutide on HF patients with varying ejection fractions or diabetes. Further, it is important to note that the advantages of SGLT-2 inhibitors, such as empagliflozin and dapagliflozin, have already been established [115, 116, 117]. Therefore, conducting more extensive clinical trials with extended periods of observation is required to examine the effects of liraglutide on patients thoroughly. By doing so, we can provide healthcare professionals with additional treatment options for their patients.

Empagliflozin and dapagliflozin, both commonly employed SGLT-2 inhibitors in clinical practice, presented a positive performance in all five metrics of the outcome of this meta-analysis and share similar mechanisms of action in patients. SGLT-2, a protein present in the luminal side of the proximal tubule epithelial cells in the S1 and S2 segments of the proximal tubule is responsible for reabsorbing glucose and sodium from urine and returning it to the circulatory system [118]. SGLT-2 inhibitors reduce the reabsorption of glucose and sodium by the kidneys by inhibiting the activity of SGLT-2. This inhibition leads to an increase in glucose reaching the distal end of the renal unit, creating an osmotic diuresis [119], whereas blocking sodium reabsorption in the proximal tubule increases sodium and chloride concentrations in the dense maculae, activates glomerular feedback, and subsequently promotes vasoactive adaptation and restoration of glomerular filtration [120]. At the systemic level, SGLT-2 inhibitors elevate plasma osmolality [121] and promote interstitial drainage, which leads to a reduction in total fluid volume, while SGLT-2 inhibitors can also induce hemoconcentration by promoting plasma volume concentration [122, 123]. Thus, SGLT-2 inhibitors accomplished the treatment of fluid retention in heart failure patients and reduced the degree of cardiac congestion. In addition to lowering workloads on the heart, SGLT-2 inhibitors can directly improve cardiac remodeling by altering the myocardial dependence on the energy supplied by glucose, instead switching to the consumption of free fatty acids, ketone bodies, and branched-chain amino acids, which results in improved myocardial energy, and left ventricular remodeling [101, 124, 125, 126]. These changes in energy metabolism contribute to improving cardiac function and alleviating symptoms in HF patients [127]. Moreover, SGLT-2 inhibitors decrease uric acid excretion in urine, reducing the deposition of uric acid crystals in the kidneys and heart. This regulation of fibroblast myolysis helps decrease inflammatory responses and damage in the heart, thereby improving the prognosis of HF [128, 129]. Due to the direct effect of empagliflozin and dapagliflozin in reducing extracellular fluid volume, these drugs can directly alleviate cardiac load, particularly by improving left ventricular hemodynamics and metabolism [124, 130]. Particularly regarding LVEF, this crucial indicator in assessing left ventricular and overall cardiac status was significantly improved. Importantly, this improvement is unrelated to the diabetic status of the patient. Previous meta-analyses have demonstrated the surprising effects of SGLT-2 inhibitors in increasing LVEF in non-diabetic HF patients [54]. The subgroup analyses in this study further revealed that SGLT-2 inhibitors are the optimal choice for increasing LVEF in HF patients with increased or decreased ejection fraction or in HF patients with comorbid T2DM. In addition to these indicators, the benefits of SGLT-2 inhibitors for cardiovascular events in patients have been thoroughly confirmed in several large-scale clinical trials [25, 48, 66, 74], solidifying their position as a first-line drug for treating HF.

In this study, irrespective of patients with or without T2DM and regardless of changes in ejection fraction, sotagliflozin was the most effective in reducing re-admission rates due to HF and all-cause mortality and was second only to liraglutide in reducing cardiovascular mortality. Unlike dapagliflozin and empagliflozin, sotagliflozin is a non-selective SGLT inhibitor with all the advantages of the existing selective SGLT-2 inhibitors mentioned earlier. SGLT-1 is also found in the human small intestine, liver, heart, and lungs [131]. Due to the high expression of SGLT-1 in the intestine or the higher concentration of sotagliflozin in the intestinal lumen than in the systemic circulation, sotagliflozin can effectively inhibit intestinal SGLT-1, thereby delaying glucose absorption in the distal small intestine and colon. This leads to reduced postprandial glucose (PPG) and improved blood glucose in individuals with T2DM, thereby reducing the incidence of cardiovascular events [132, 133]. Furthermore, since SGLT-1 is highly expressed in the sarcolemma of myocardial cells in human autopsy hearts and mouse-perfused hearts [134], sotagliflozin may offer more direct cardiovascular benefits to patients and has the potential to replace dapagliflozin and empagliflozin as the first-line option for clinicians in the treatment of heart failure. Future research focusing on this aspect is necessary, and additional large-scale RCTs are required to validate whether sotagliflozin is a more favorable choice for HF patients than other SGLT-2 inhibitors. More RCTs are also needed to evaluate further the efficacy of different anti-diabetic agents in HF patients with or without T2DM and in HF patients with reduced or preserved baseline ejection fraction.

This study has certain limitations. Due to insufficient relevant studies, we need to be cautious about the rankings this study received, according to SUCRA. For example, regarding the outcome of NTpro-BNP, only one study included the effect of liraglutide on the reduction of NTpro-BNP levels in patients with HF. As a result, the utility of liraglutide was ranked first; thus, more clinical trials are needed to validate the efficiency of liraglutide. In the included studies, there was an insufficient number of HF patients without T2DM. Additionally, in subgroup analyses, the types of drugs included in each subgroup were not comprehensive, preventing a thorough comparison of the effects of each drug on different types of HF patients. Meanwhile, it is highly probable that sotagliflozin may offer similar additional benefits to HF patients without T2DM. In HF patients with T2DM, dapagliflozin emerged as the most effective drug for reducing cardiovascular mortality. However, if a study explored the effectiveness of liraglutide on cardiovascular mortality in HF patients with T2DM, the results of this study may change. Finally, this NMA exclusively discussed the use of each drug alone. The situation of HF patients is complex, often requiring combination therapy. The effect of each drug may vary for each patient, and the combination of two drugs may have a synergistic effect. For instance, sotagliflozin can increase the secretion of GLP1 in patients, and DPP-4 can prolong the half-life of GLP1 [135]. Therefore, there may be benefits to the combined use of these two drugs. However, more studies are needed in the future to analyze the advantages of combining different anti-diabetic drugs for patients.

## 5. Conclusions

This Bayesian NMA demonstrates that sotagliflozin may be the optimal choice of drug for HF patients with or without T2DM. Sotagliflozin significantly reduced the probability of re-admission due to HF and all-cause death in HF patients. Larger-scale prospective studies are required to assess whether sotagliflozin is more effective than other SGLT-2 inhibitors.

## Availability of Data and Materials

The original contributions presented in the study are included in the article. Further inquiries can be directed to the corresponding author.

## Supplementary Material

Supplementary material associated with this article can be found, in the online version, at https://doi.org/10.31083/RCM26154.
