# Supplementary material for: Effect of Hypoglycemic Drugs on Patients with Heart Failure with or without T2DM: A Bayesian Network Meta-analysis
Source: Rev Cardiovasc Med. 2025 Mar 21;26(3):26154. doi: 10.31083/RCM26154 (PMC11951290; doi:10.31083/RCM26154)
Supplement: Supplementary file 1 [file 2153-8174-26-3-26154-s1.zip › Supplementary figures--R1.docx]

**
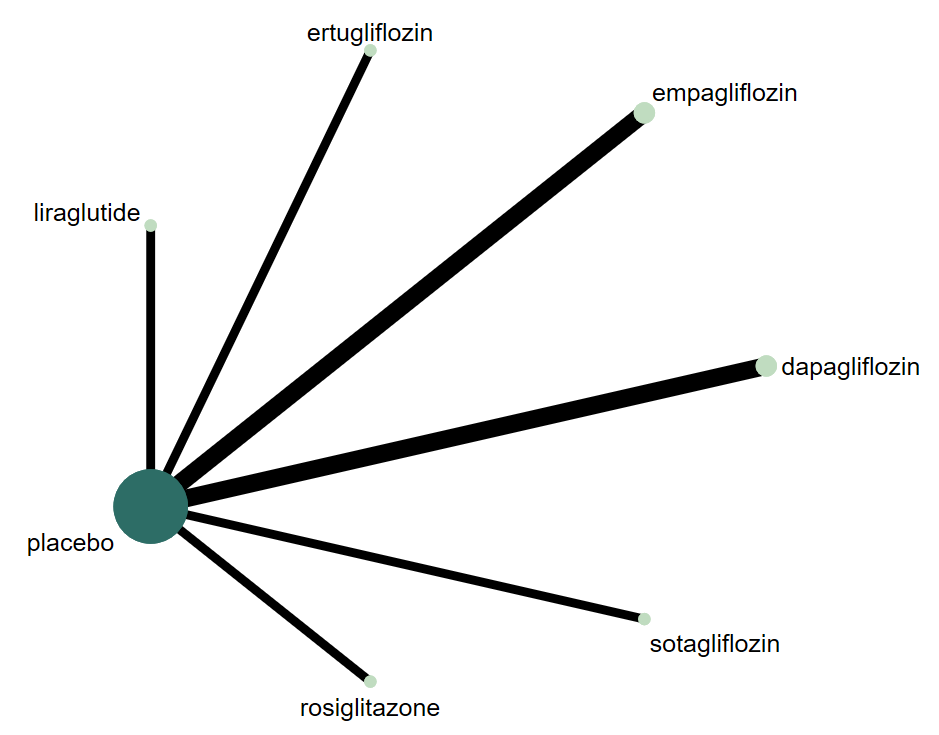
**

**Supplementary Fig. 1.** The network graph for HErEF patients' readmission due to HF.


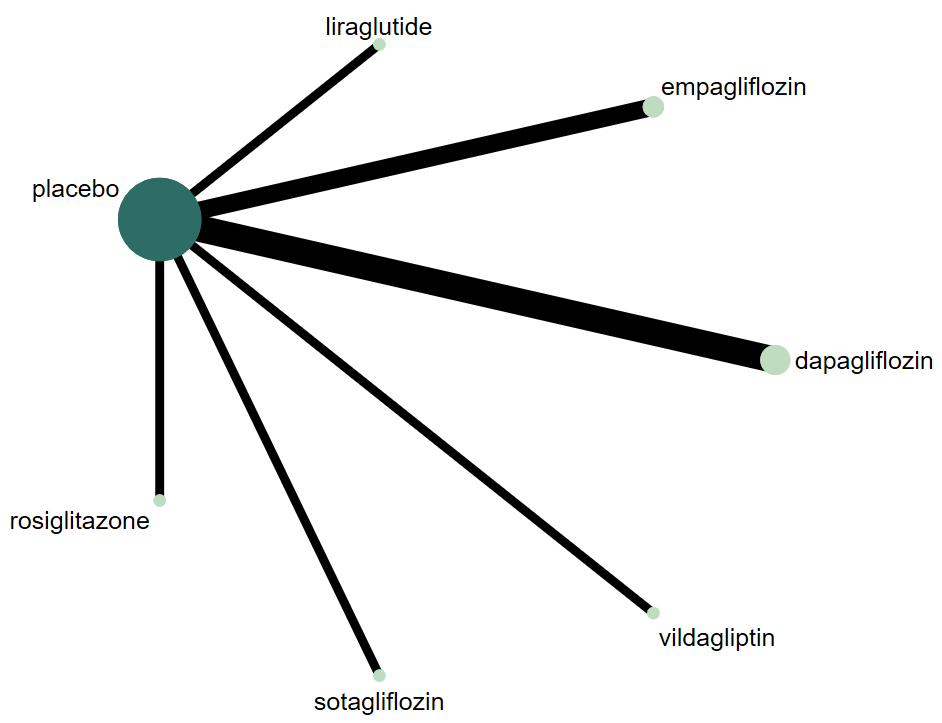


**Supplementary Fig. 2.** The network graph for HErEF patients' all-cause death.

**
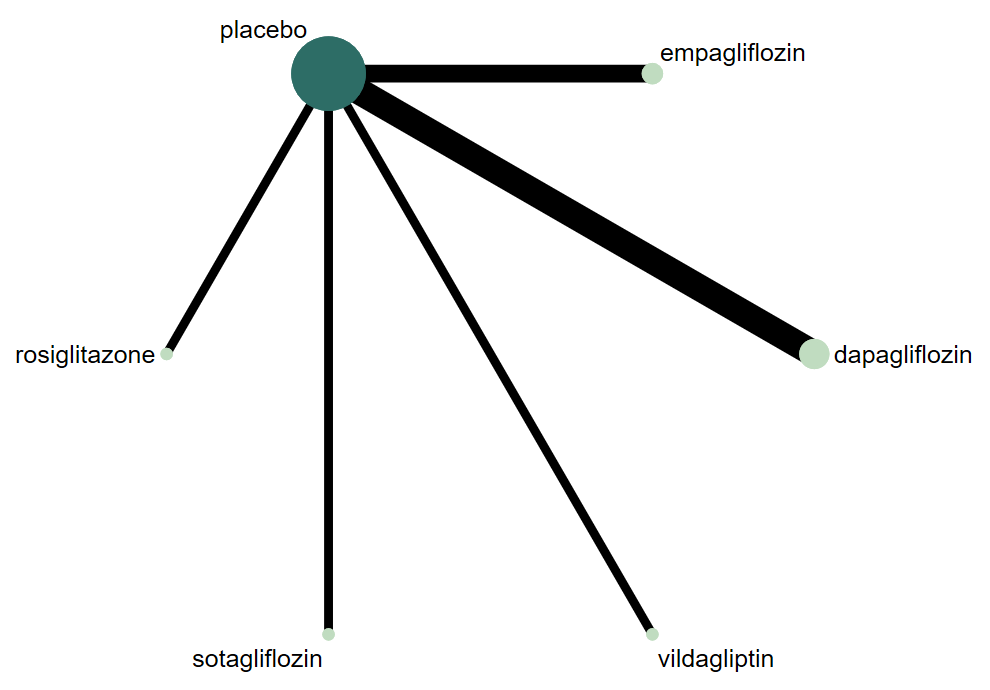
**

**Supplementary Fig. 3.** The network graph for HErEF patients' cardiovascular death.


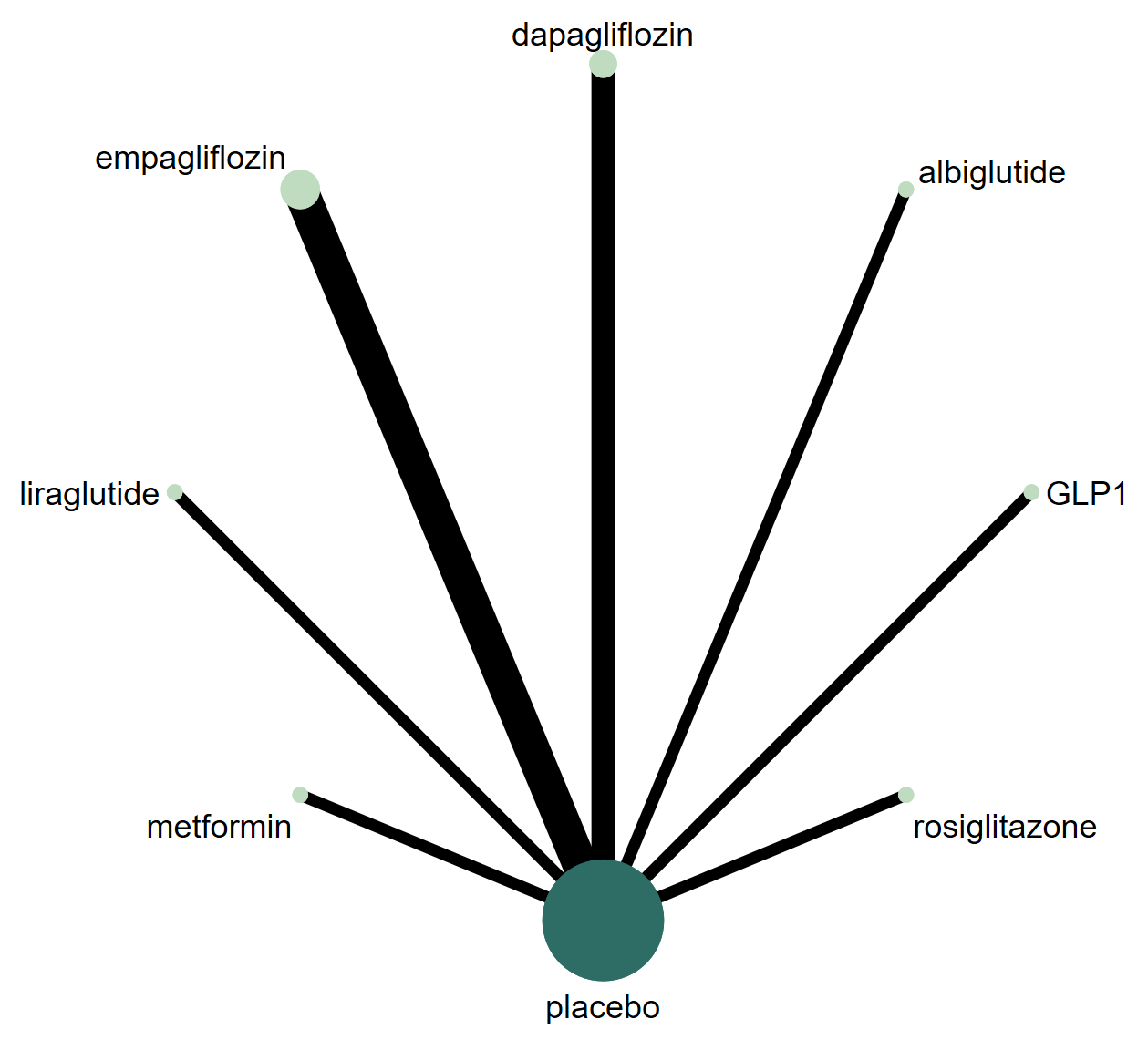


**Supplementary Fig. 4.** The network graph for HErEF patients' LVEF.


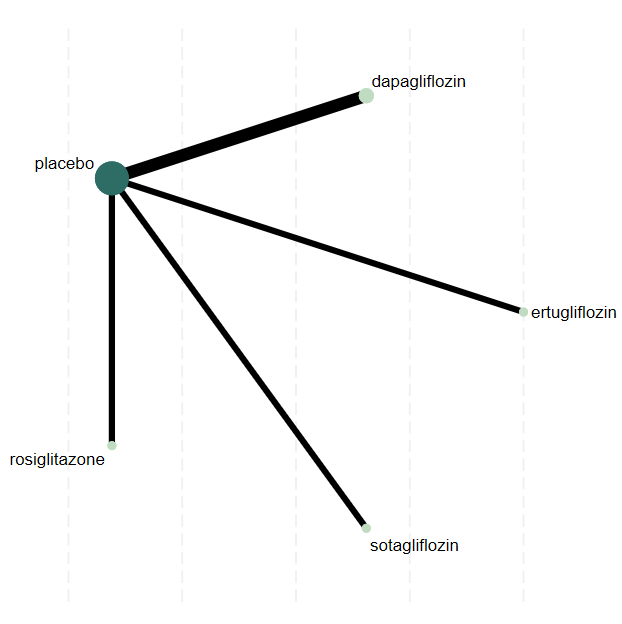


**Supplementary Fig. 5.** The network graph for readmission due to HF in HF patients with T2DM.


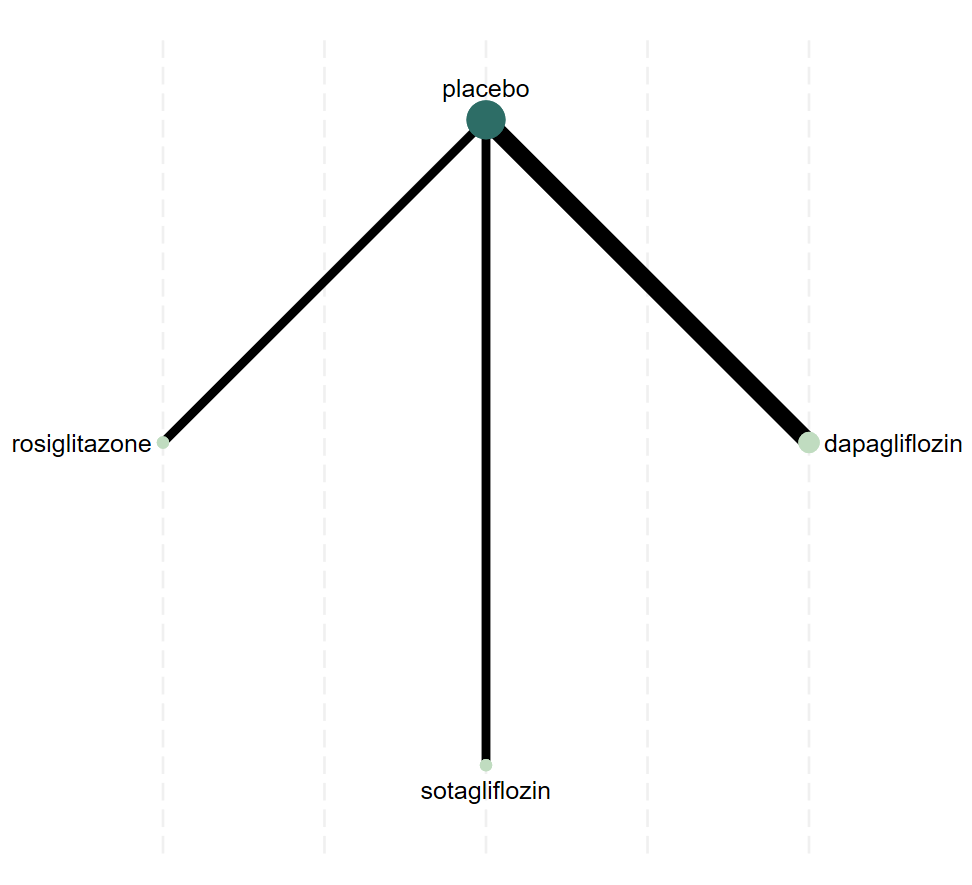


**Supplementary Fig. 6.** The network graph for all-cause death in HF patients with T2DM.


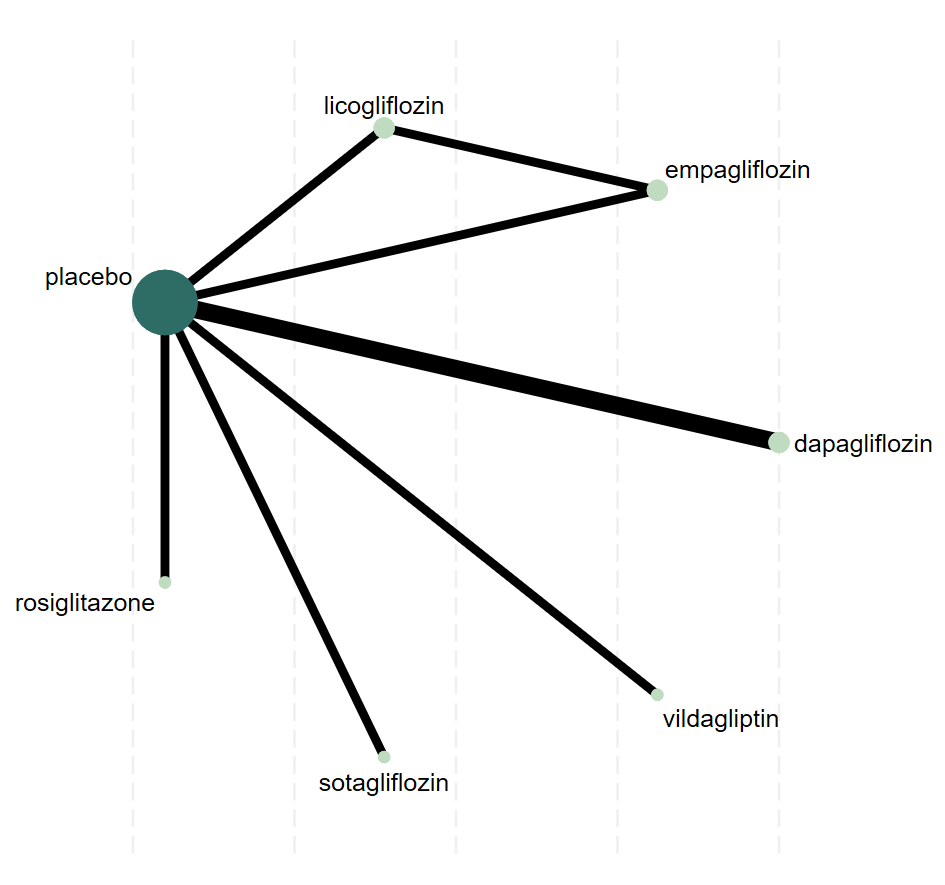


**Supplementary Fig. 7.** The network graph for cardiovascular death in HF patients with T2DM.


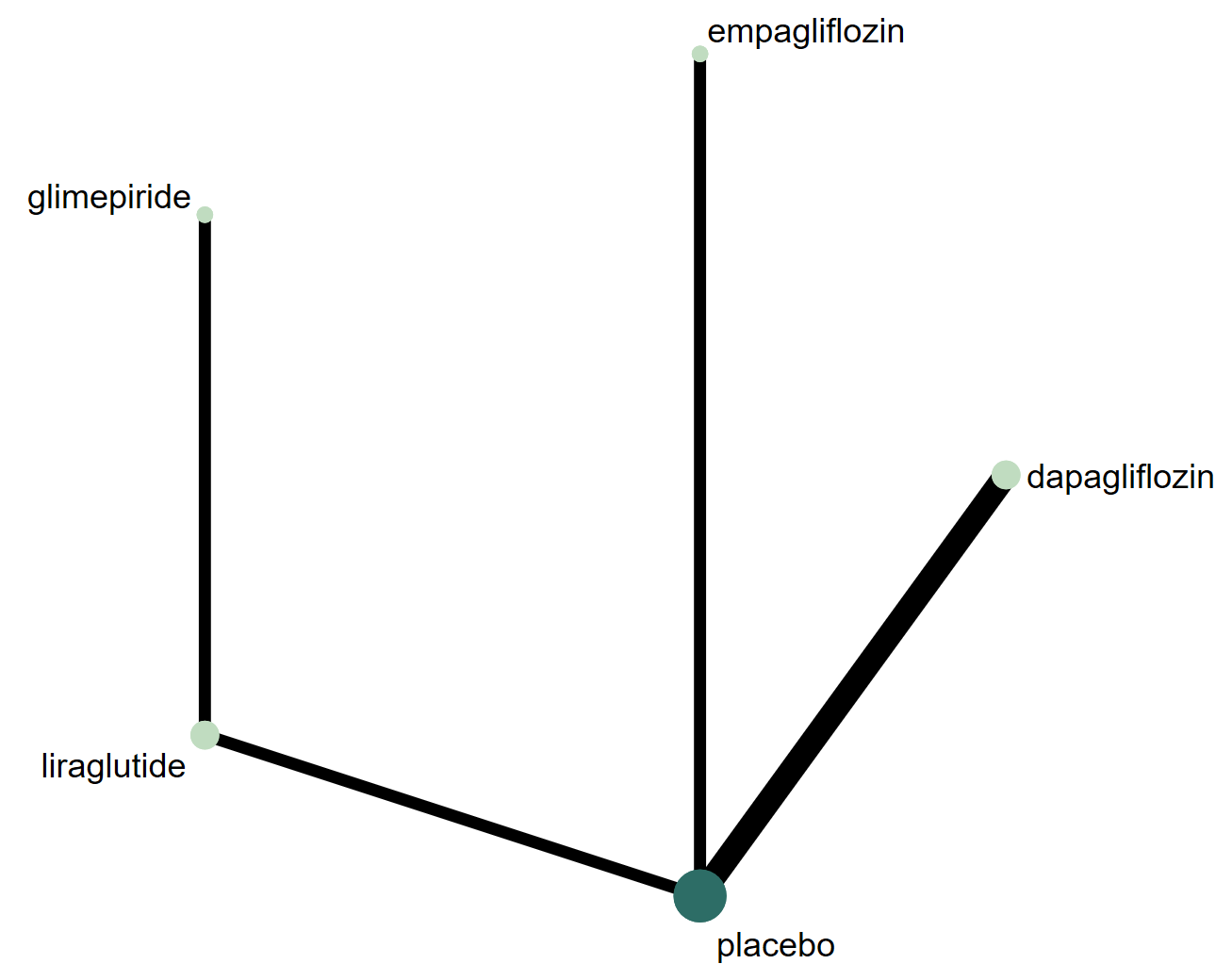


**Supplementary Fig. 8.** The network graph for changes in LVEF in HF patients with T2DM.


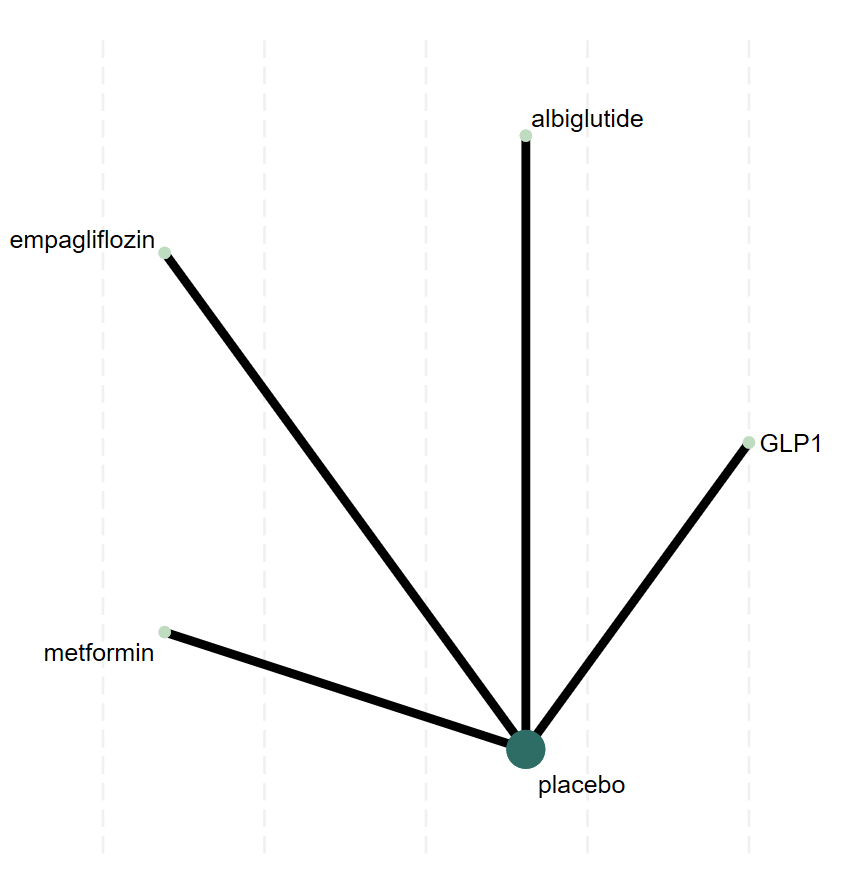


**Supplementary Fig. 9.** The network graph of changes in LVEF in HF patients without T2DM.


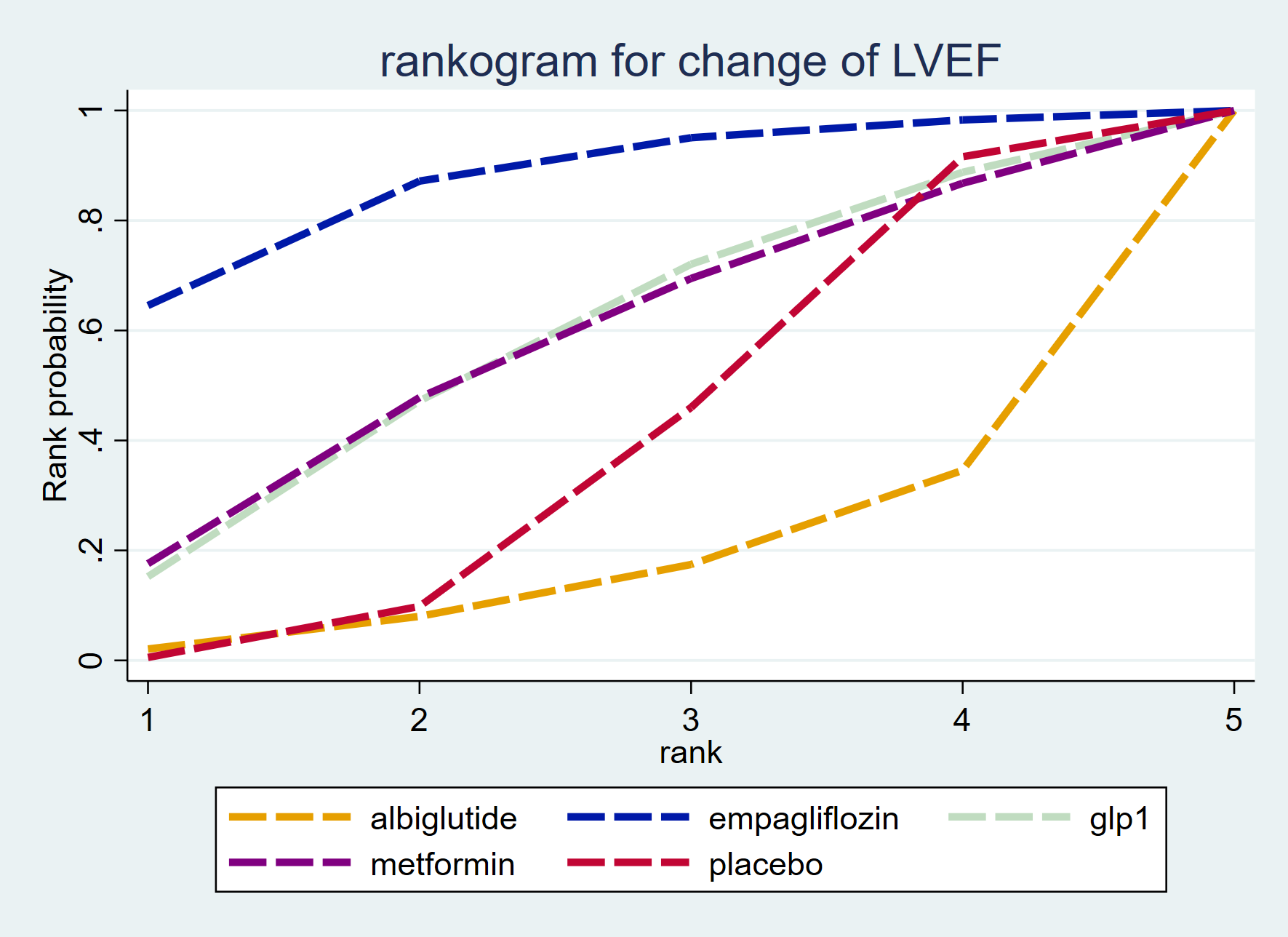


**Supplementary Fig. 10.** Rankogram for change of LVEF in HF patients without T2DM.


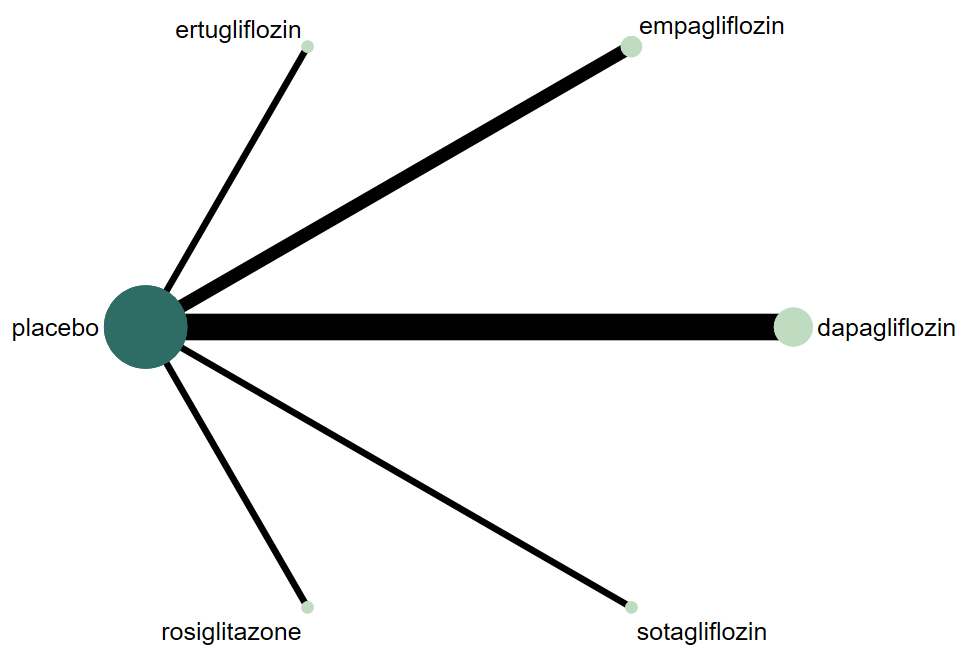


**Supplementary Fig. 11.** The network graph of follow-up more than one year patients' readmission due to HF.


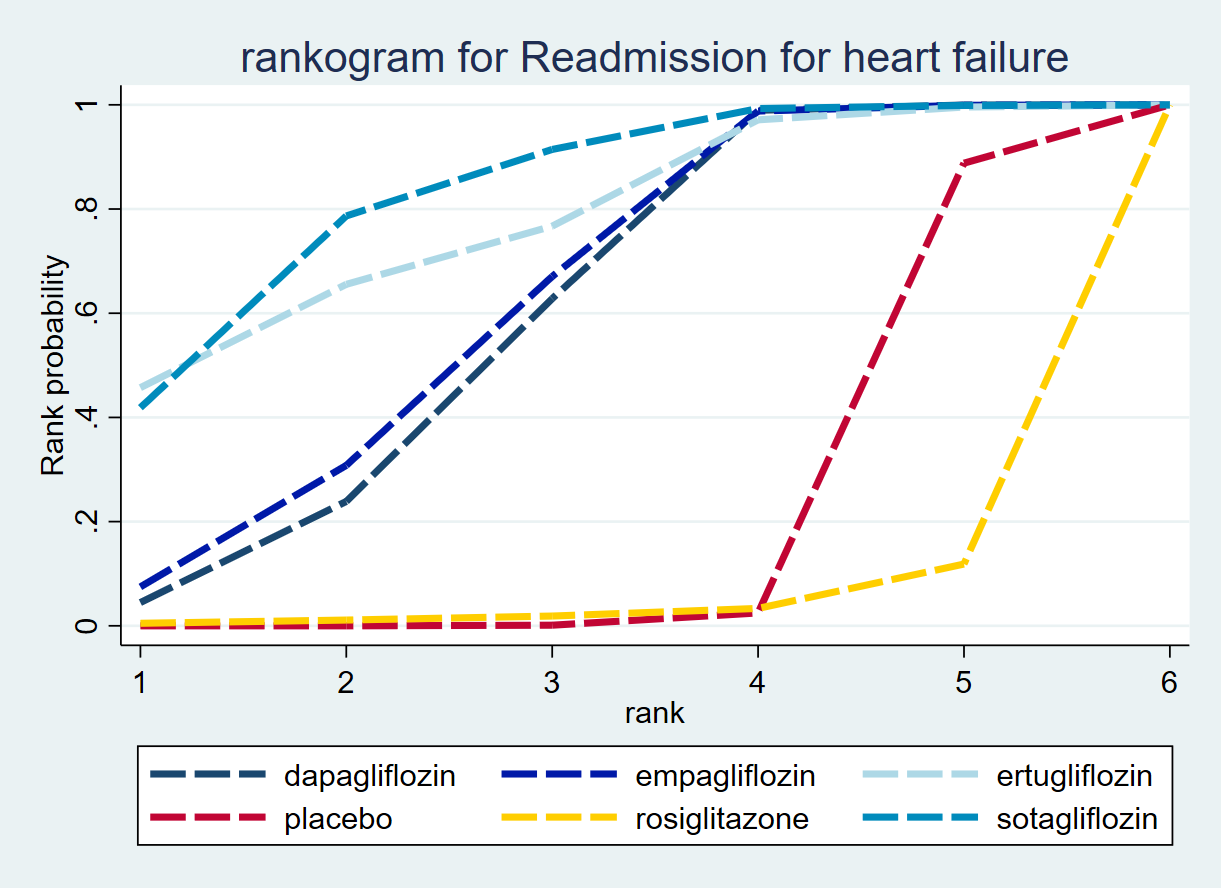


**Supplementary Fig. 12.** Rankogram for follow-up more than one year patients' readmission due to HF.


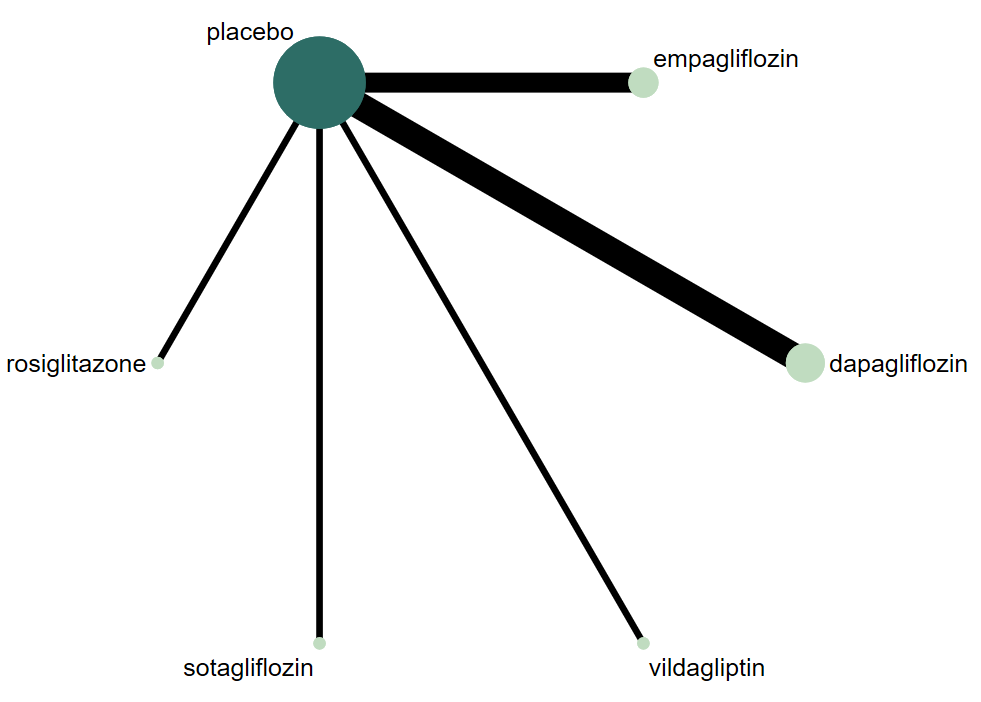


**Supplementary Fig. 13.** The network graph of follow-up more than one year patients' all-cause death.


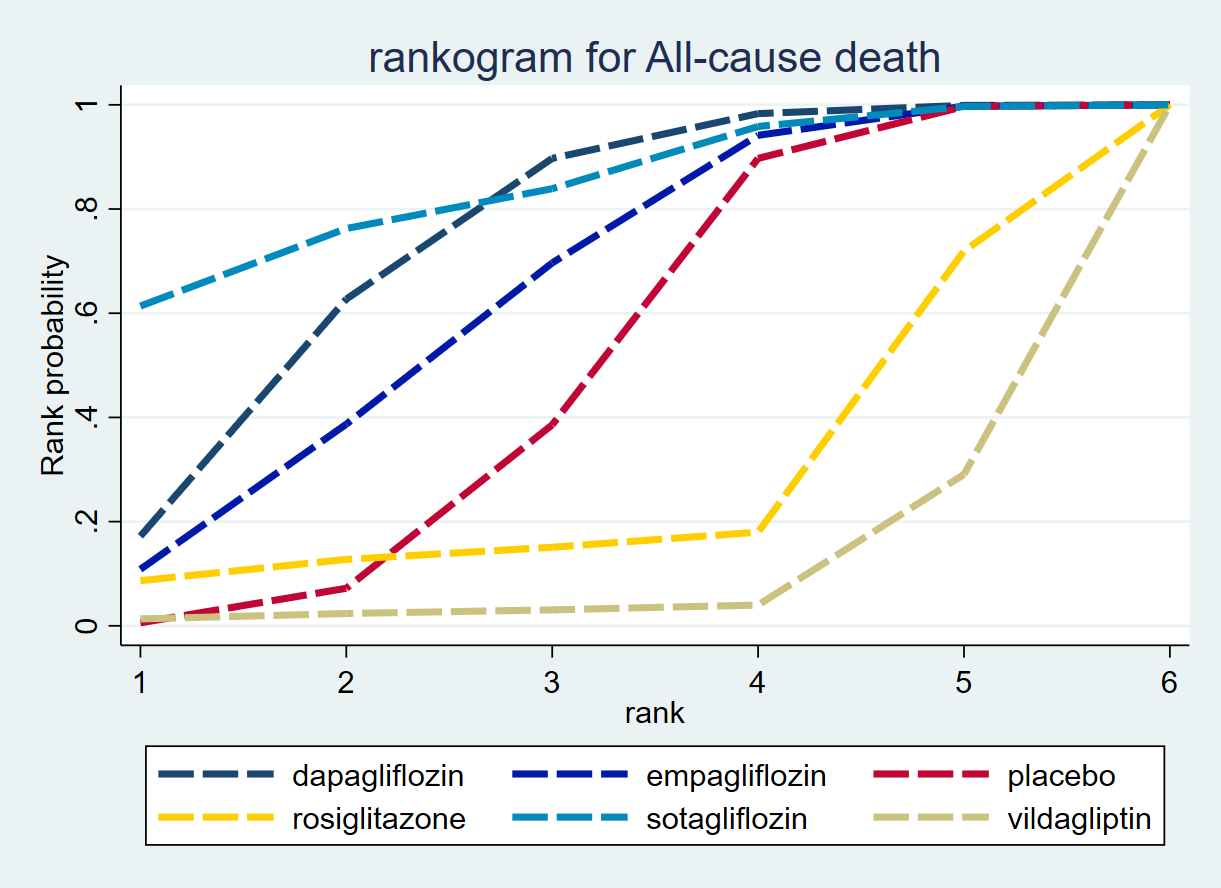


**Supplementary Fig. 14.** Rankogram for follow-up more than one year patients' all-cause death.


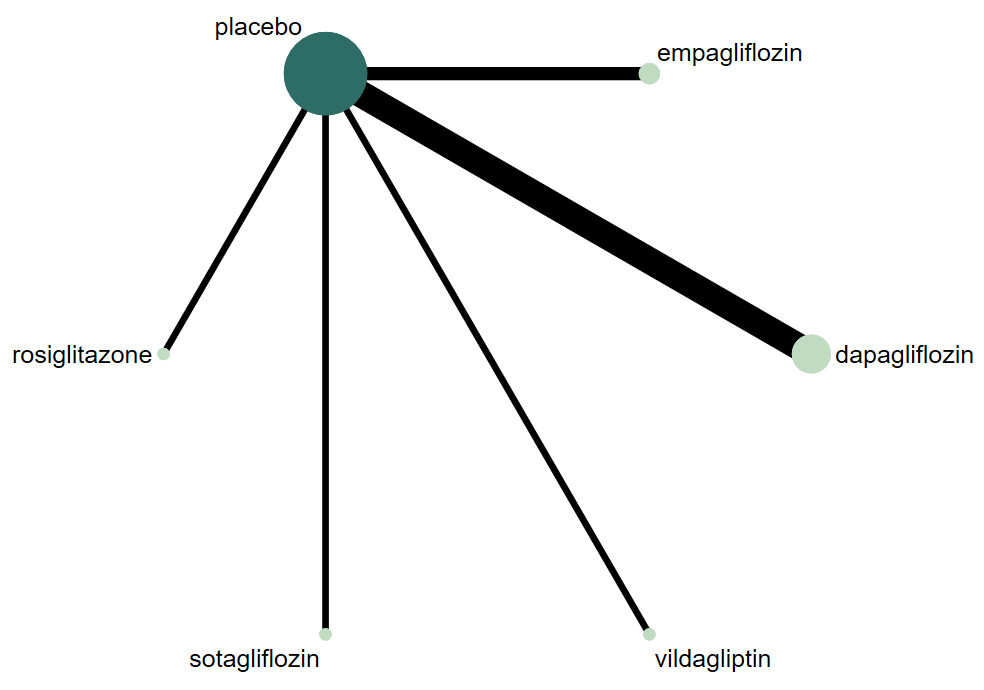


**Supplementary Fig. 15.** The network graph of follow-up more than one year patients' cardiovascular death.


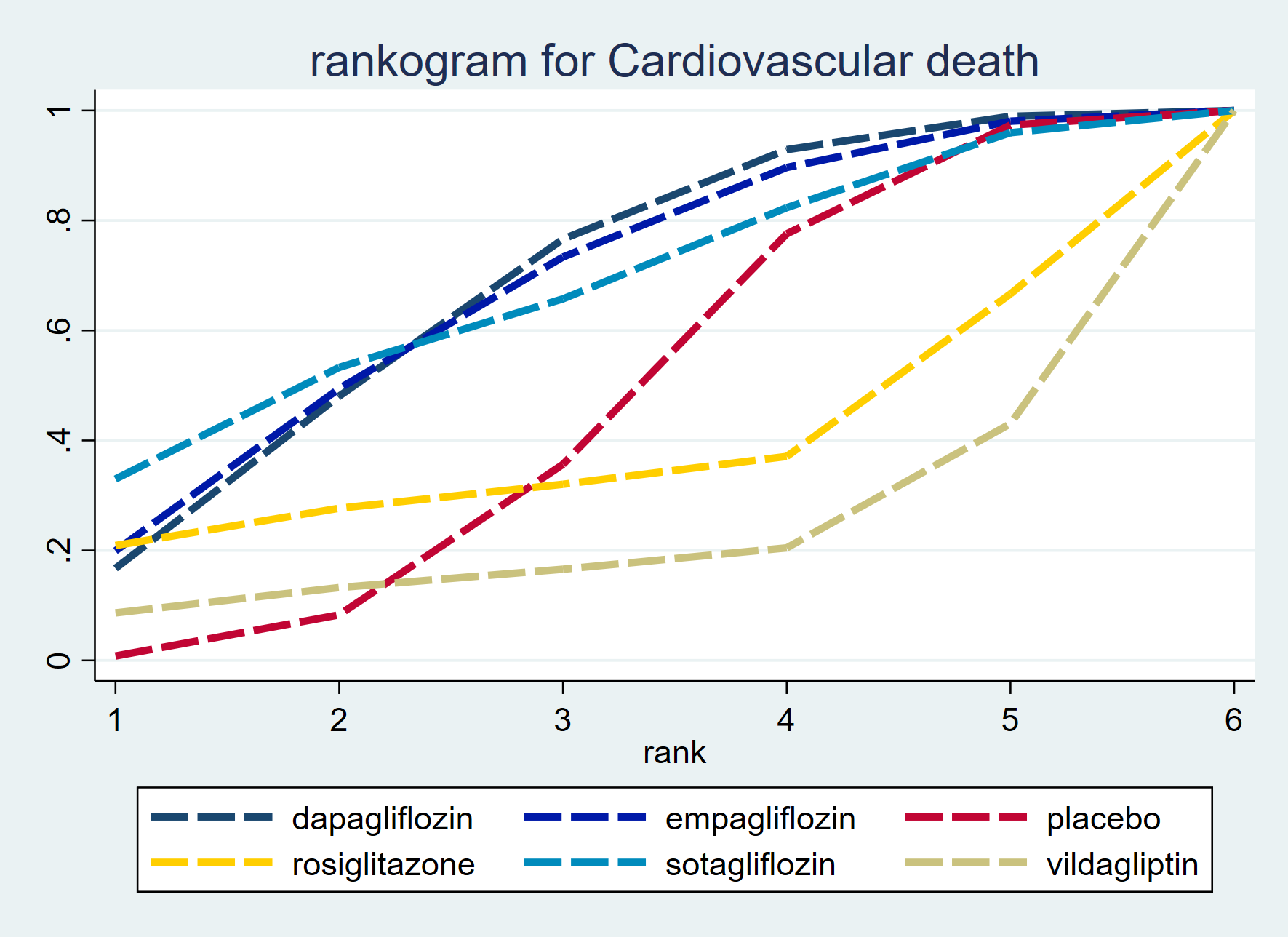


**Supplementary Fig. 16.** Rankogram for follow-up more than one year patients' cardiovascular death.


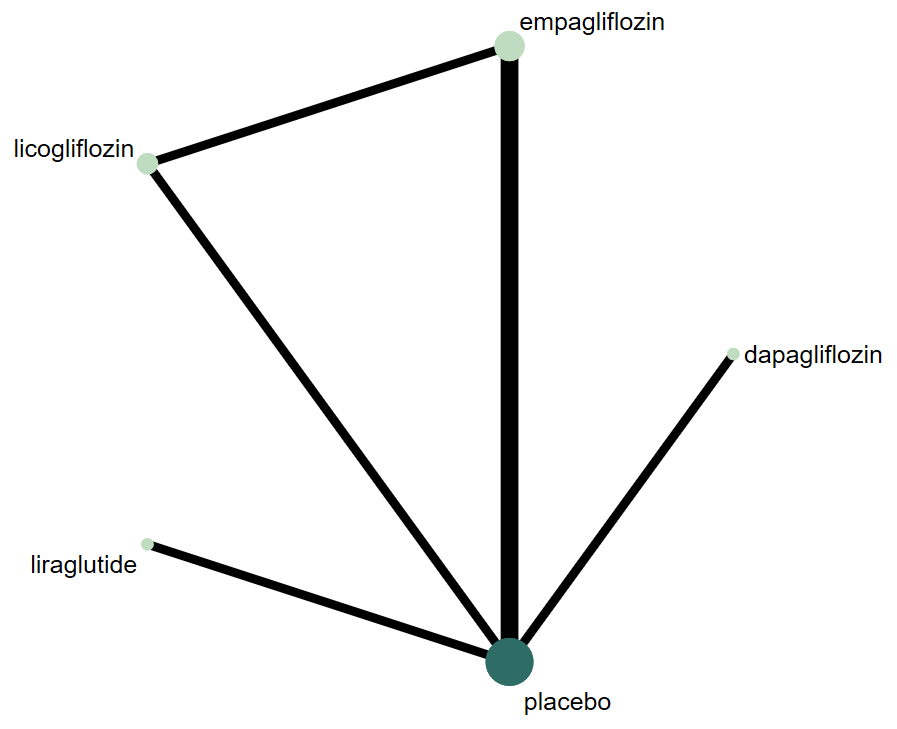


**Supplementary Fig. 17.** The network graph of follow-up less than one year patients' cardiovascular death.


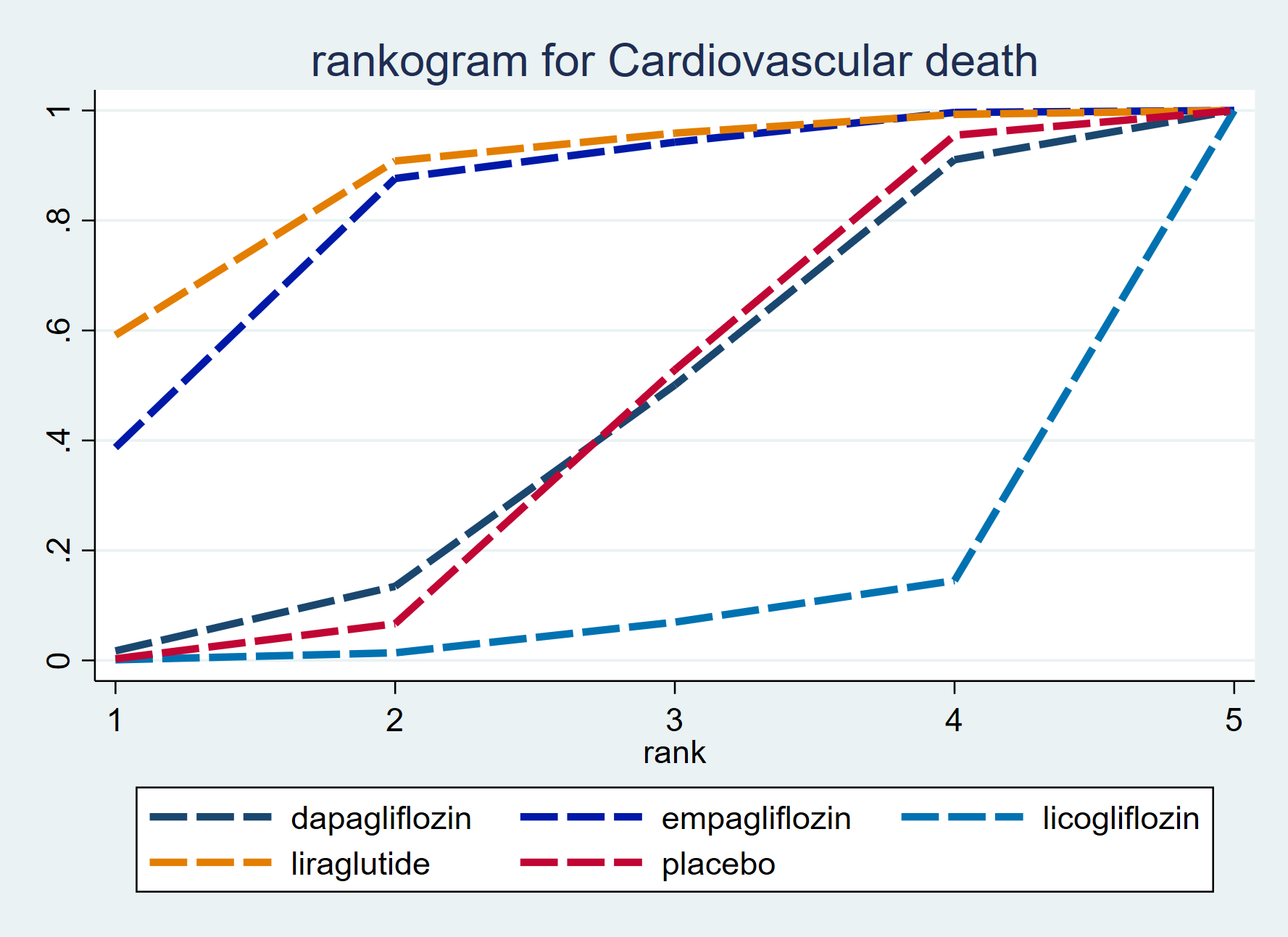


**Supplementary Fig. 18.** Rankogram for follow-up less than one year patients' cardiovascular death.


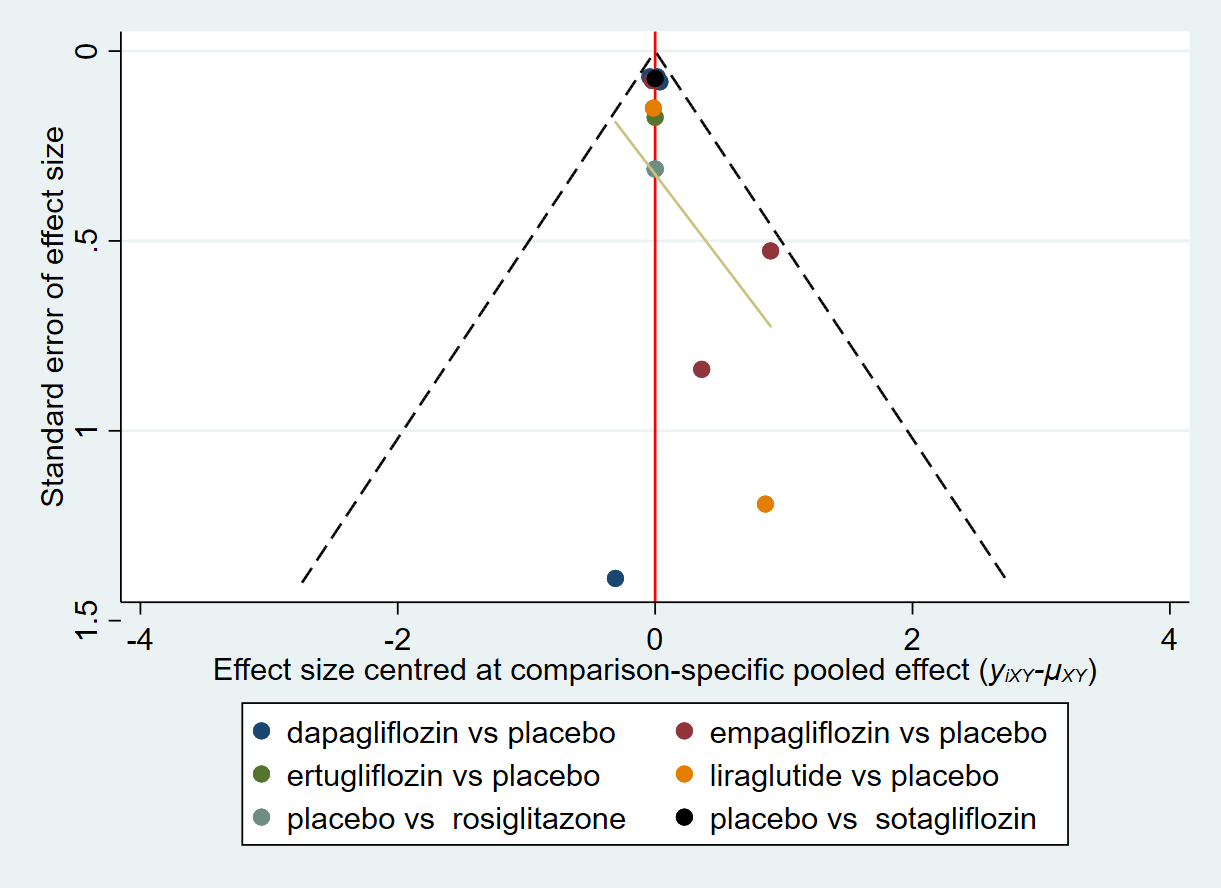


**Supplementary Fig. 19.** Publication bias of readmission due to HF.

**
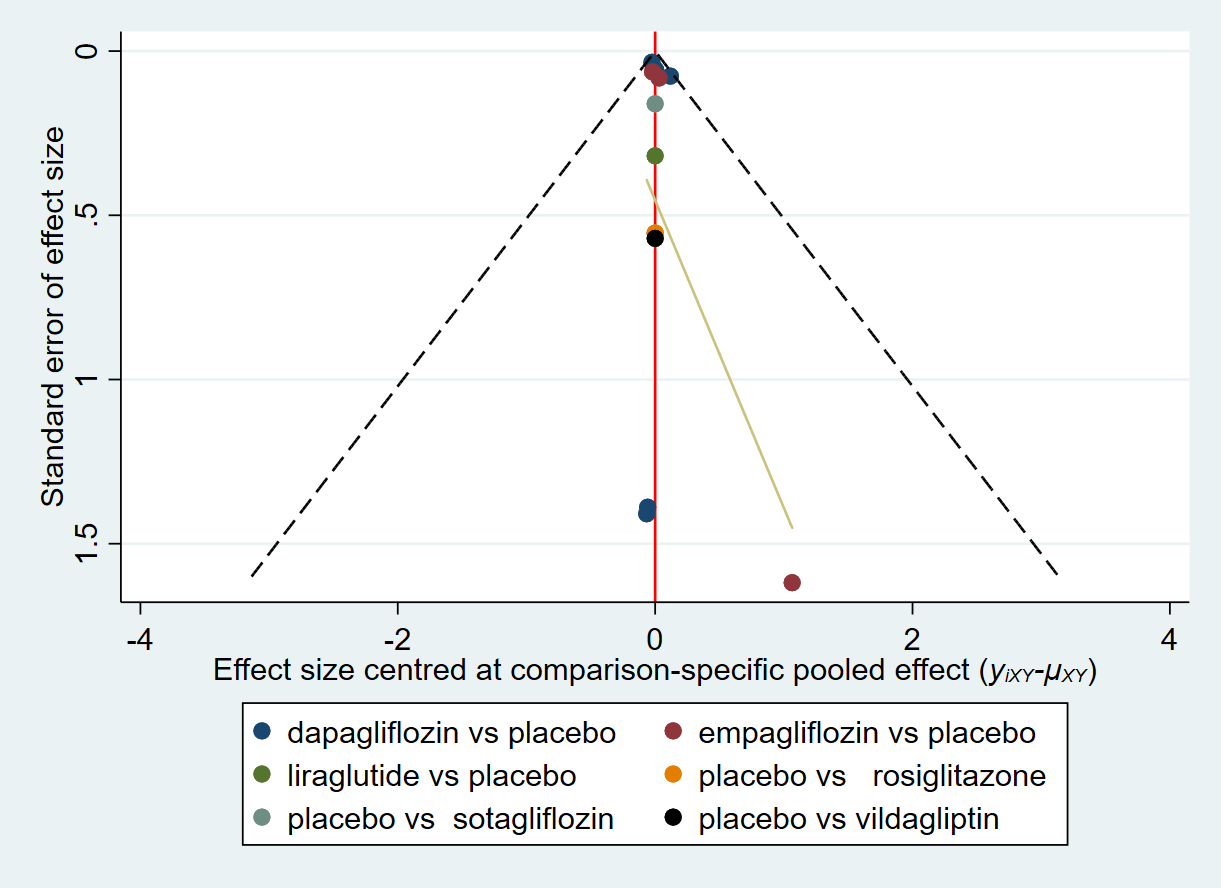
**

**Supplementary Fig. 20.** Publication bias of all-cause death.

**
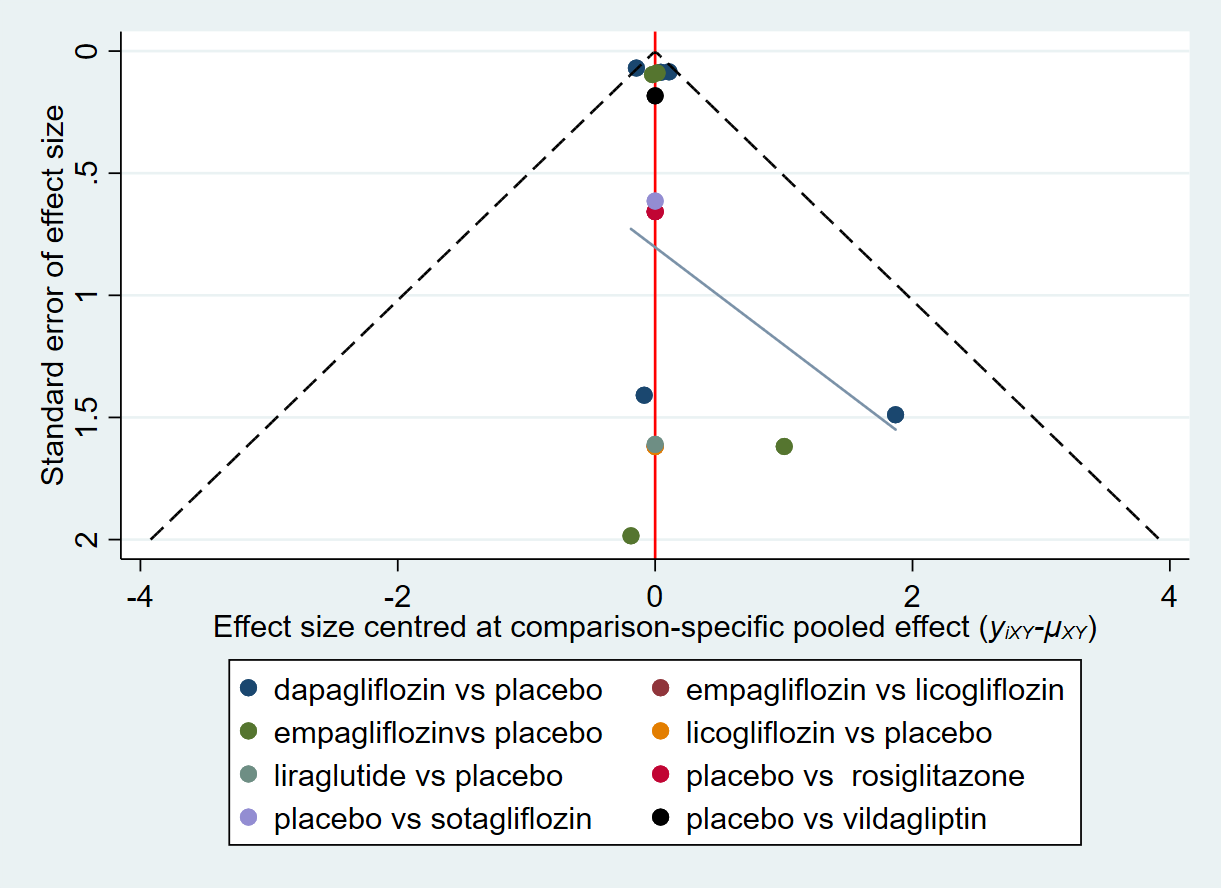
**

**Supplementary Fig. 21.** Publication bias of cardiovascular death.

**
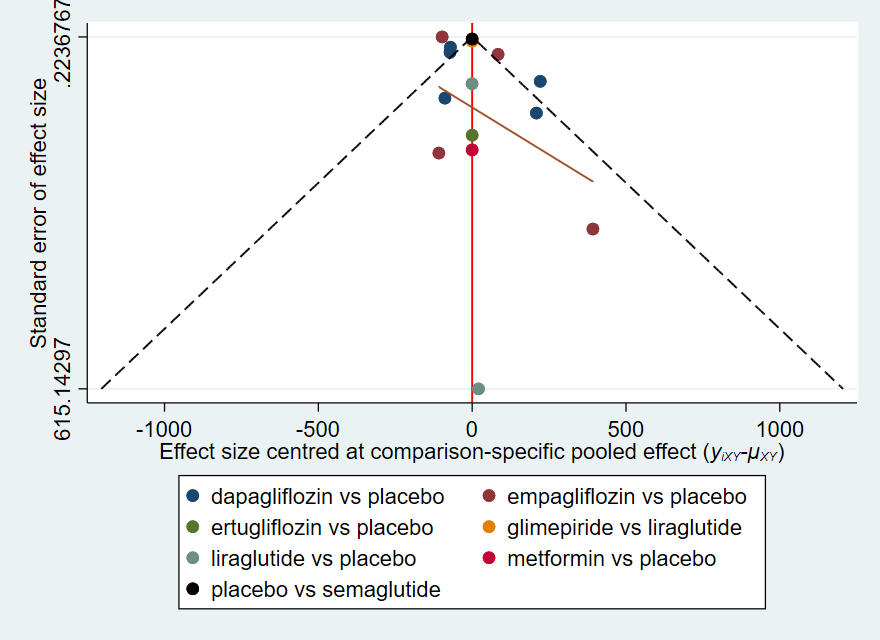
**

**Supplementary Fig. 22.** Publication bias of NTpro-BNP.

**
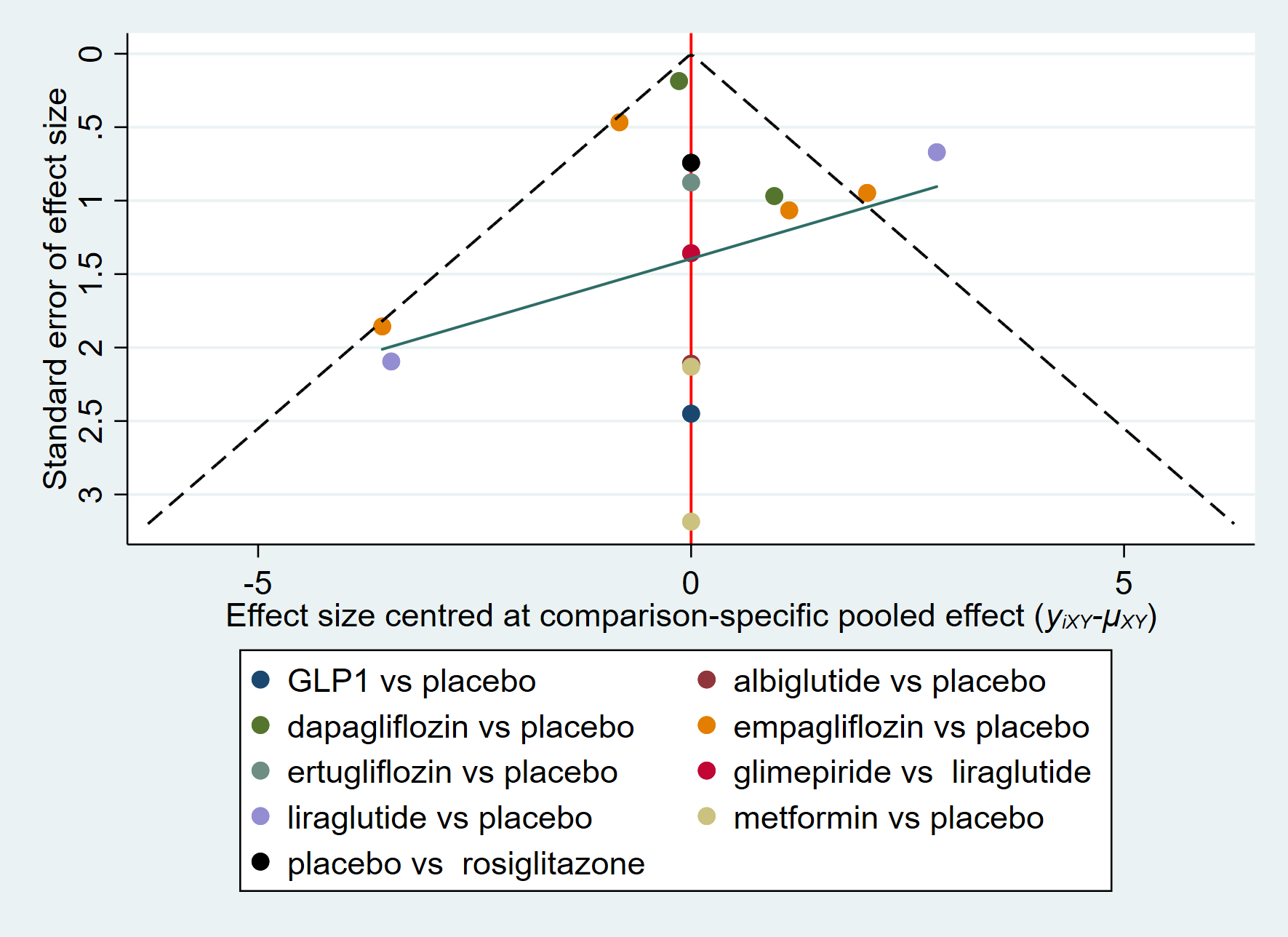
**

**Supplementary Fig. 23.** Publication bias of LVEF.

**
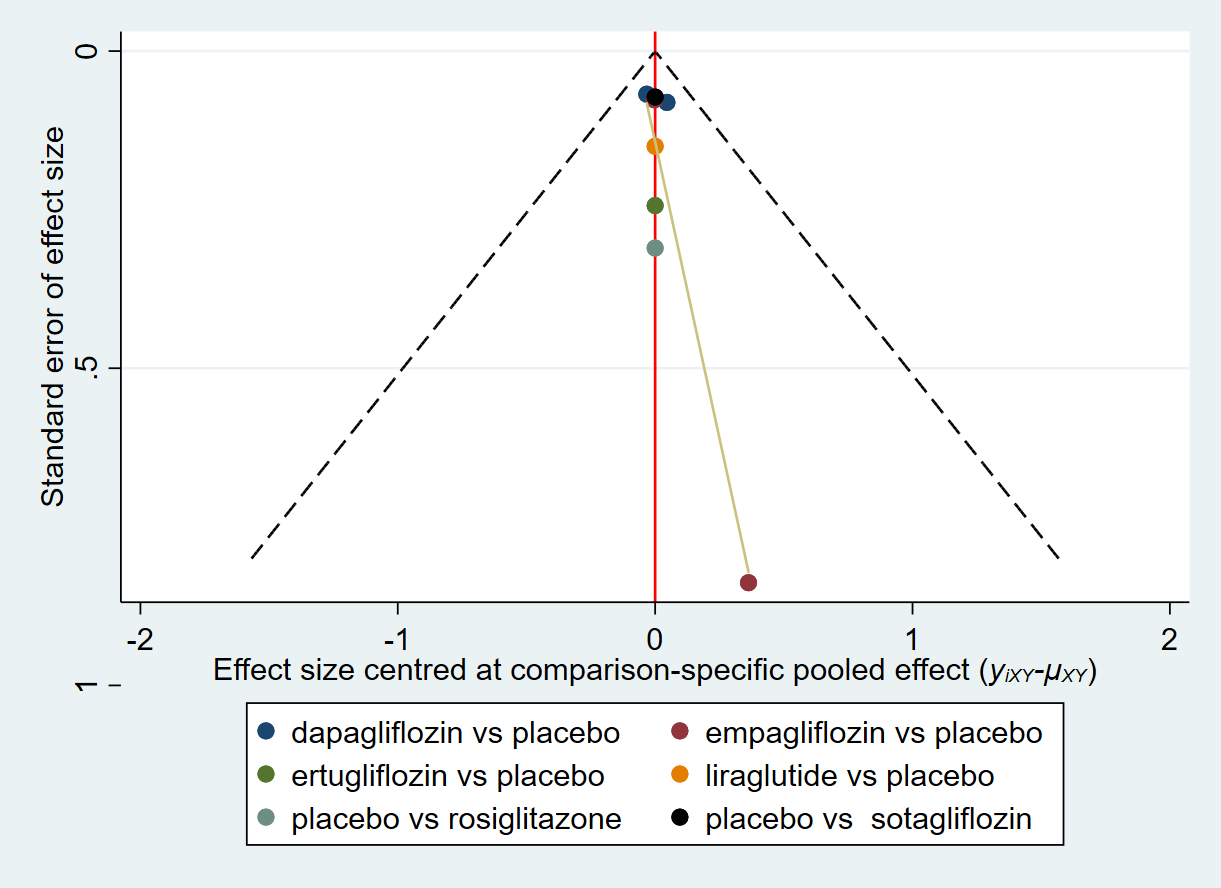
**

**Supplementary Fig. 24.** Publication bias of HErEF patients' readmission due to HF.

**
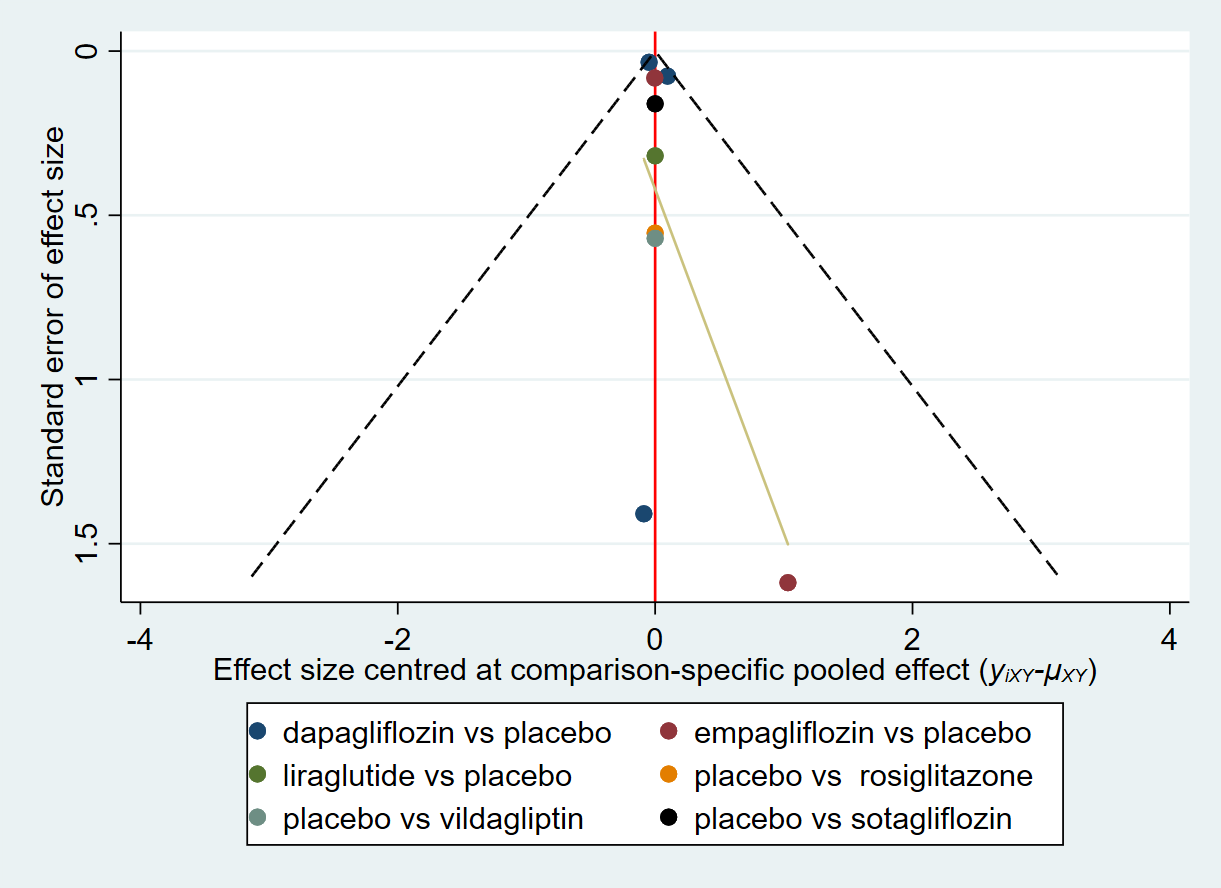
**

**Supplementary Fig. 25.** Publication bias of HErEF patients' all-cause death.

**
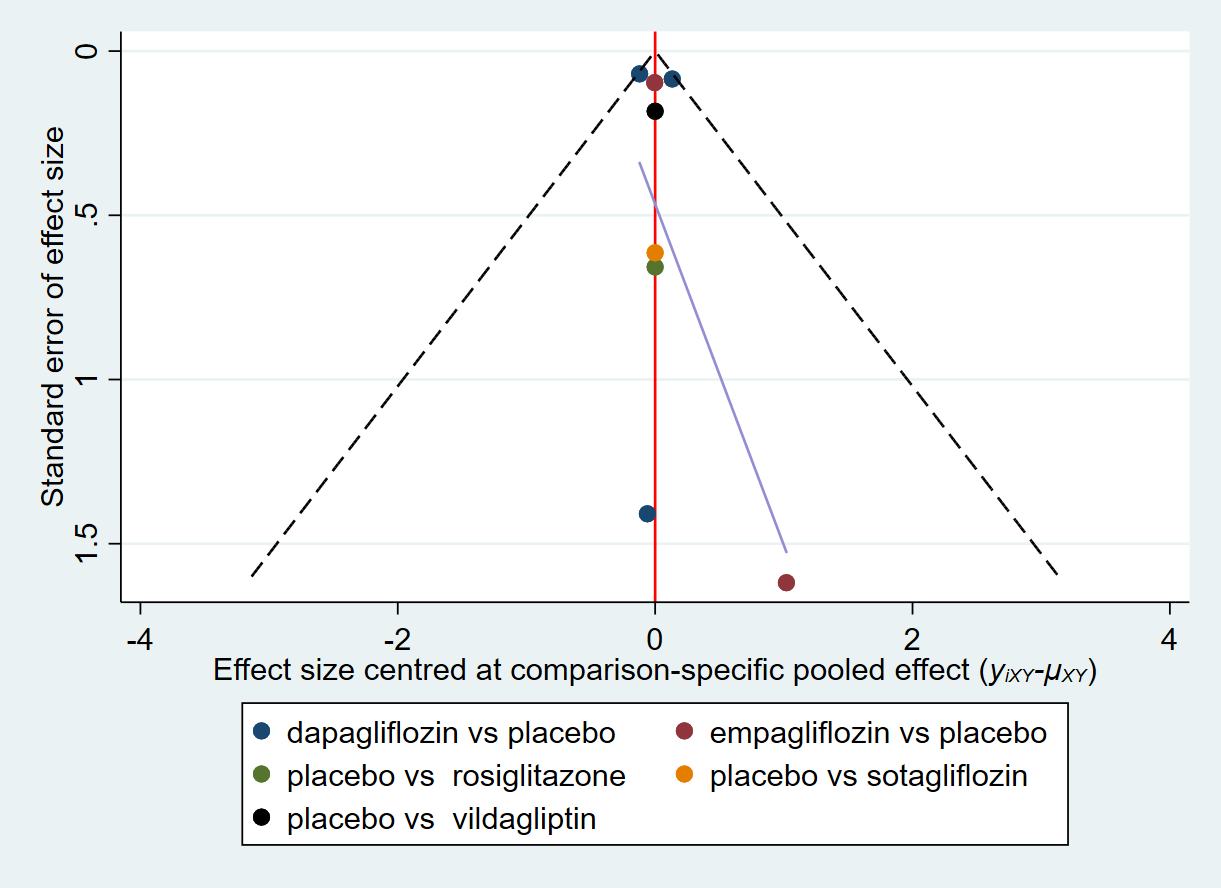
**

**Supplementary Fig. 26.** Publication bias of HErEF patients' cardiovascular death.

**
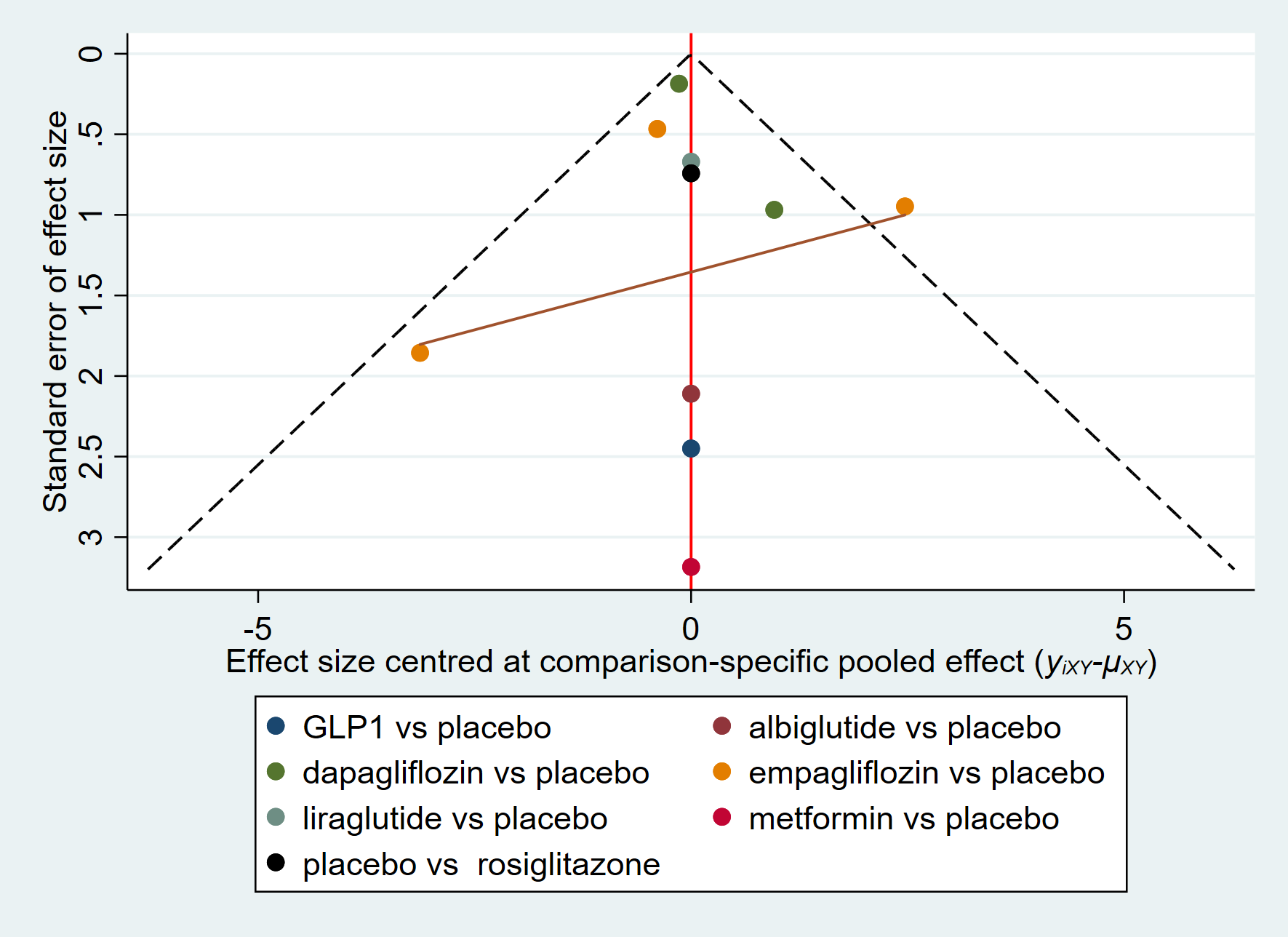
**

**Supplementary Fig. 27.** Publication bias of HErEF patients' LVEF.


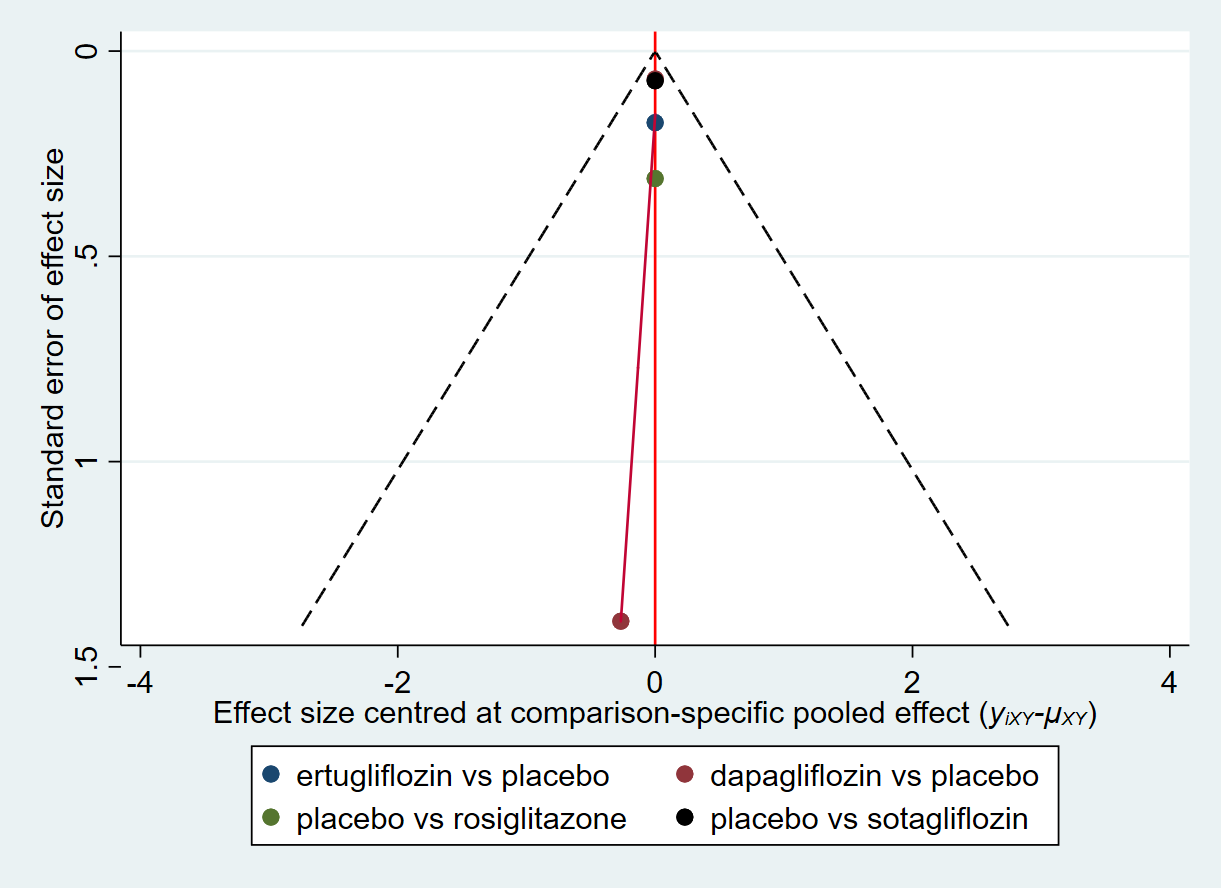


**Supplementary Fig. 28.** Publication bias of readmission due to HF in HF patients with T2DM.

**
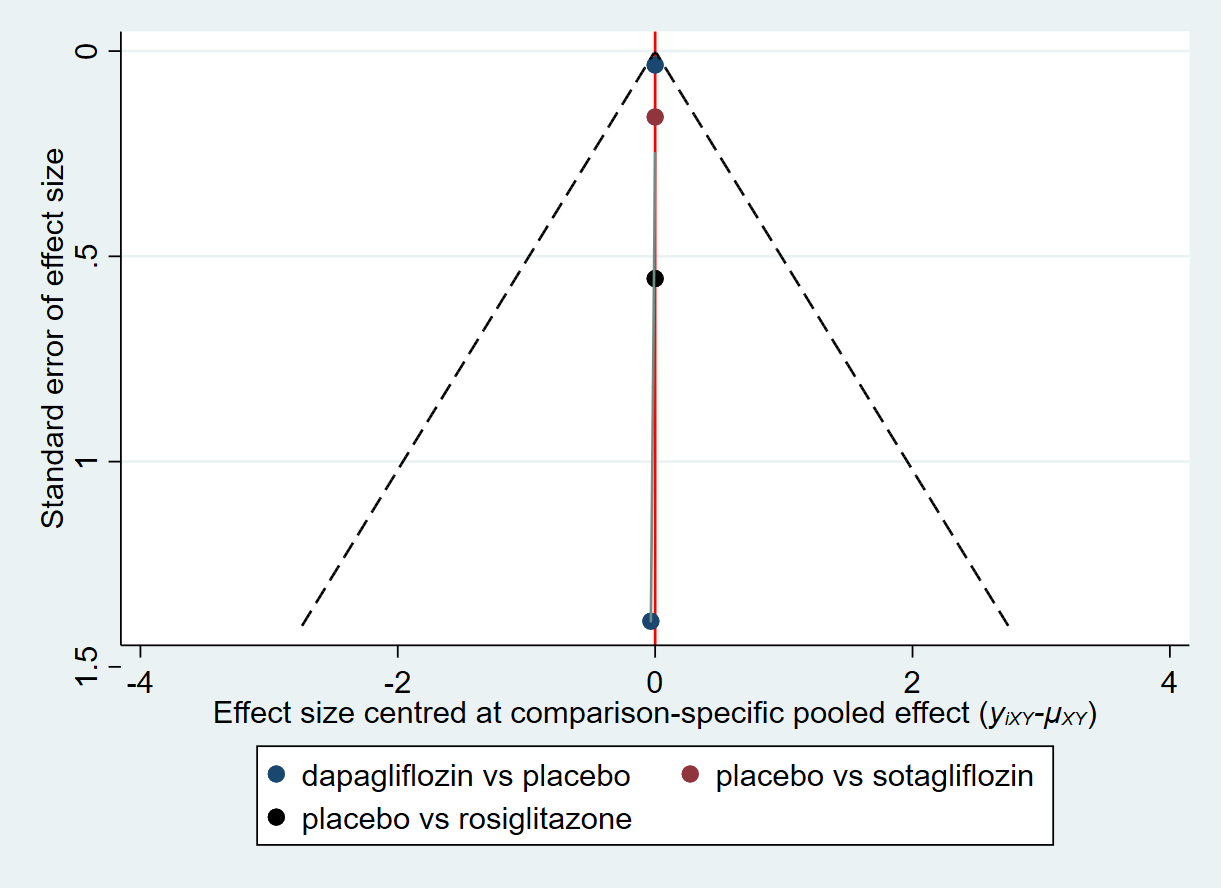
**

**Supplementary Fig. 29.** Publication bias of all-cause death in HF patients with T2DM.

**
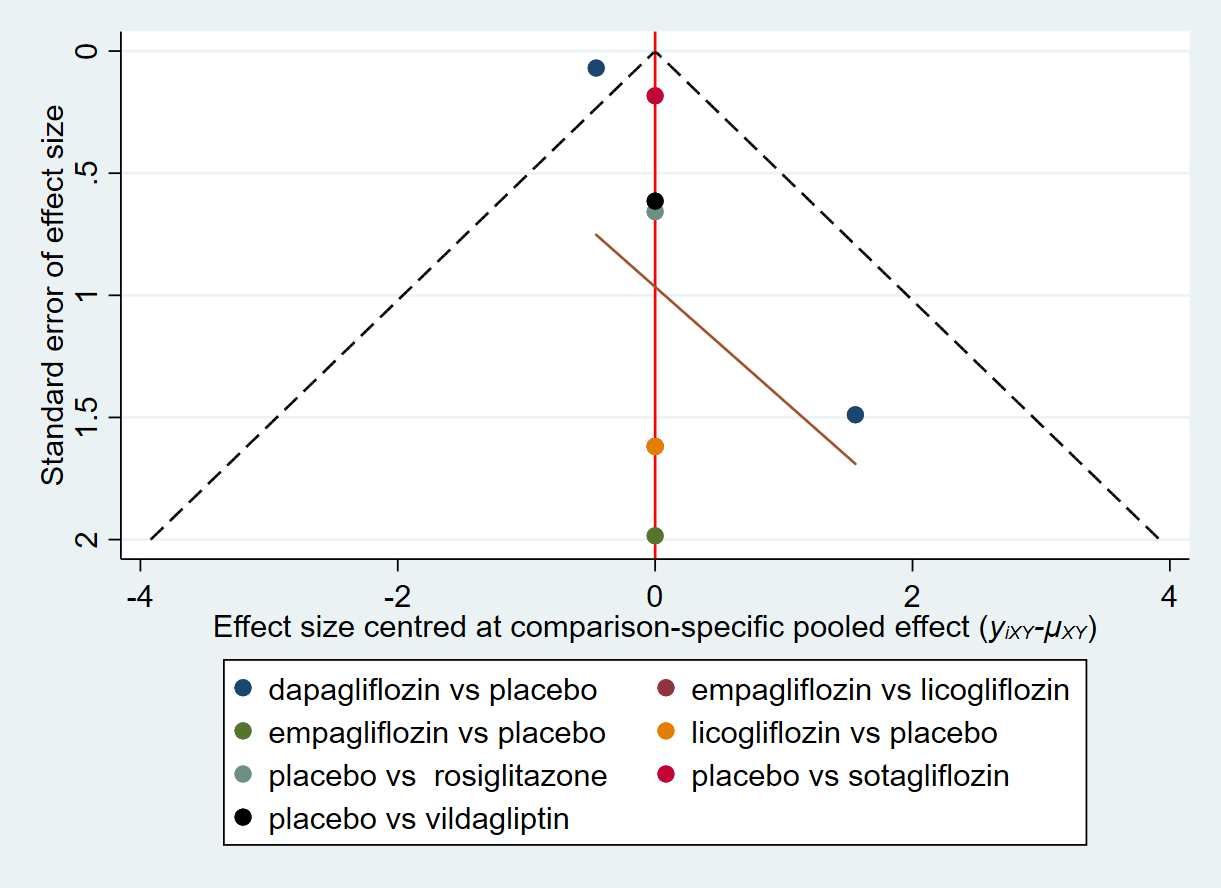
**

**Supplementary Fig. 30.** Publication bias of cardiovascular death in HF patients with T2DM.

**
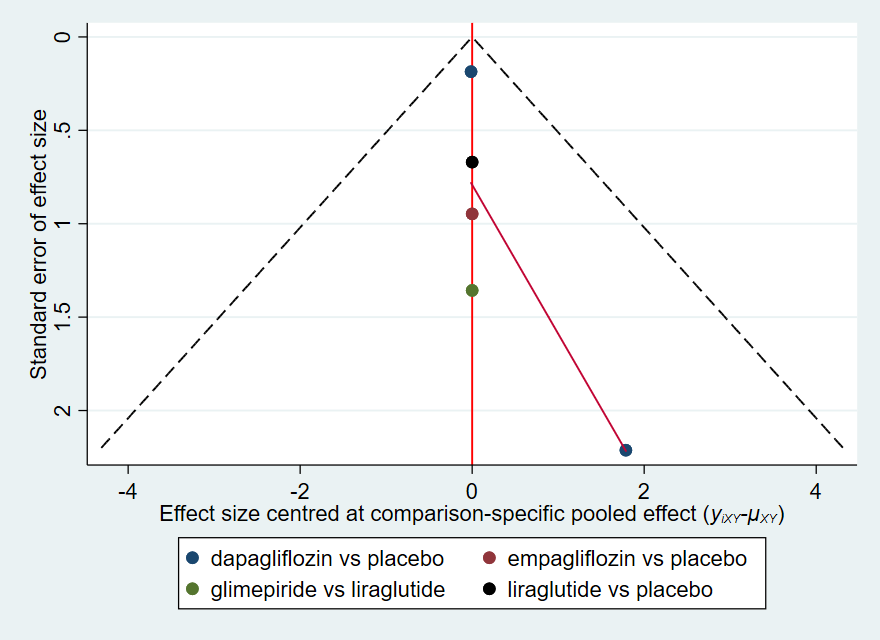
**

**Supplementary Fig. 31.** Publication bias of changes in LVEF in HF patients without T2DM.


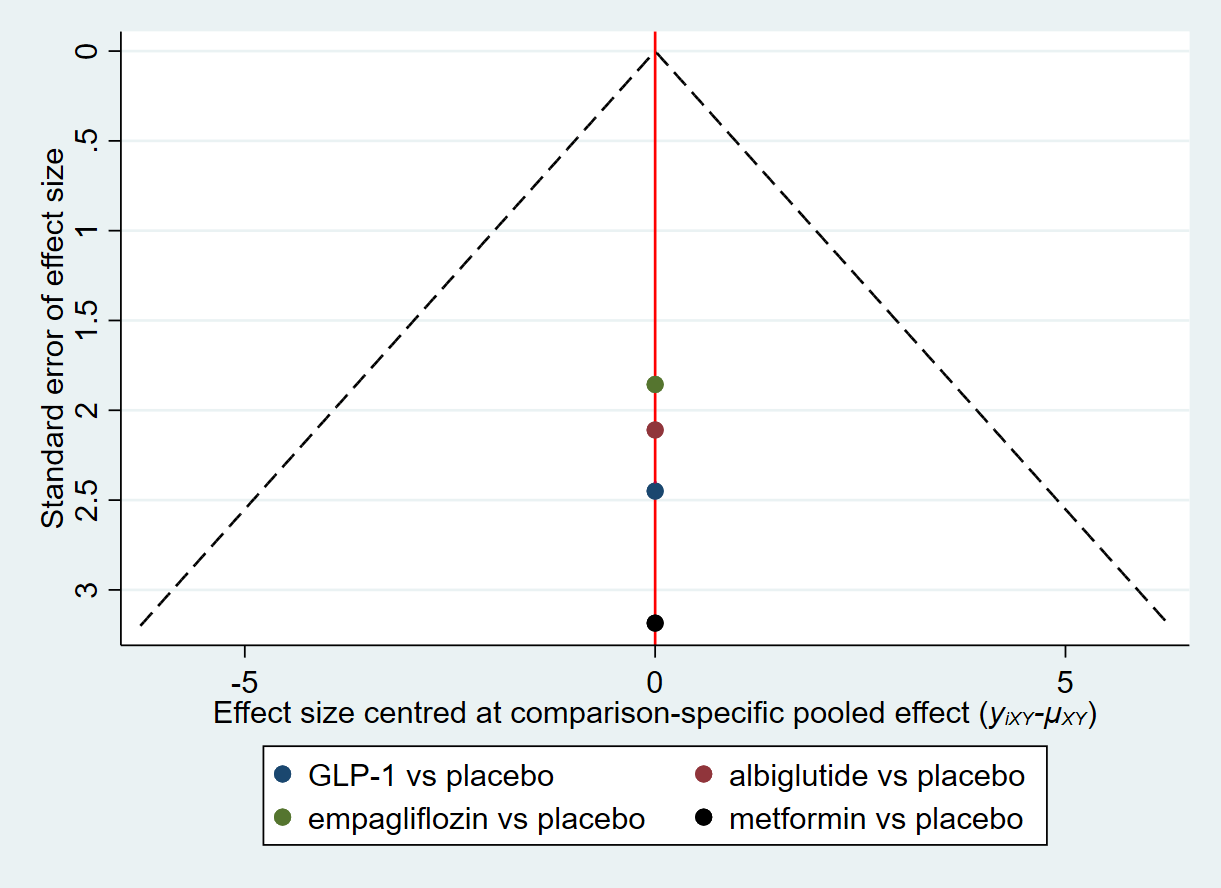


**Supplementary Fig. 32.** Publication bias of changes in LVEF in HF patients without T2DM.

**
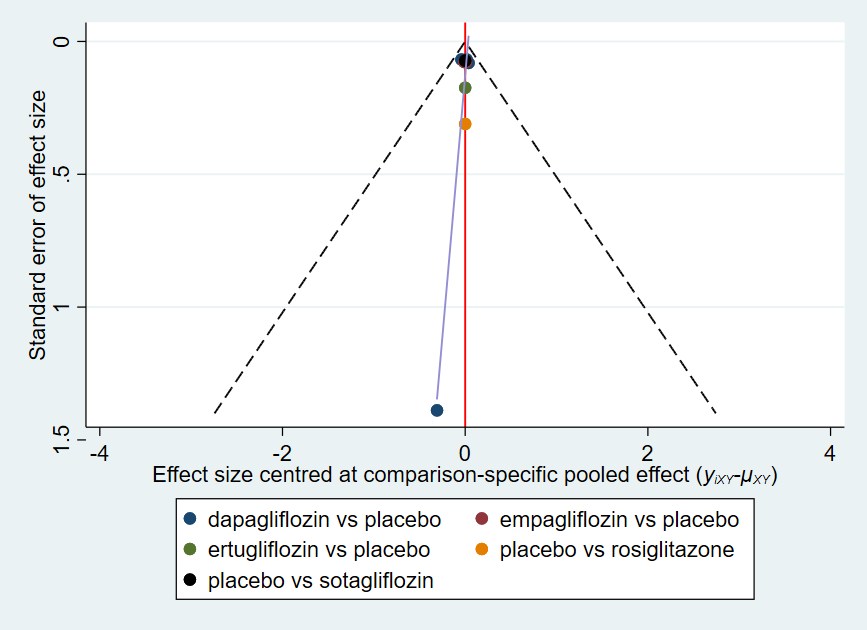
**

**Supplementary Fig. 33.** Publication bias of changes in follow-up more than one year patients' readmission due to HF.

**
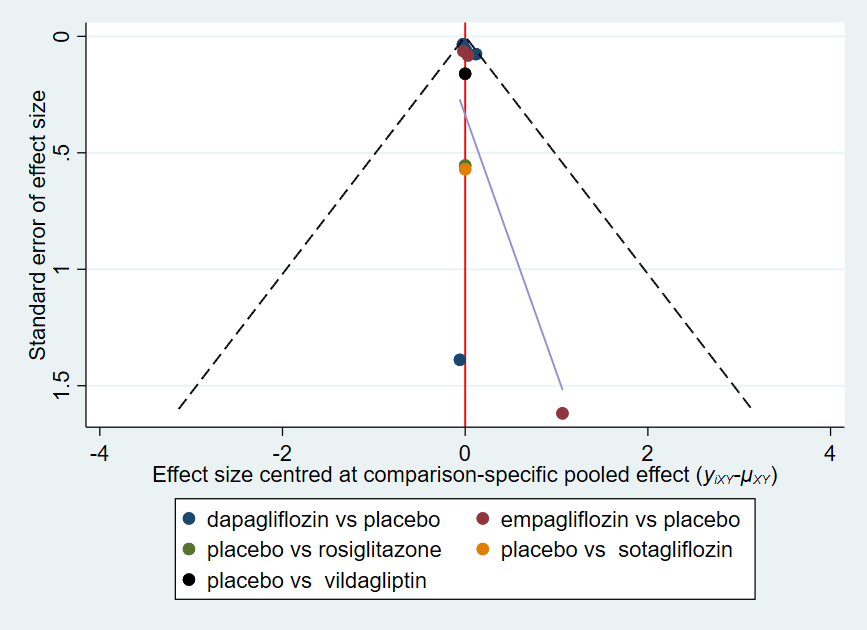
**

**Supplementary Fig. 34.** Publication bias of changes in follow-up more than one year patients' all-cause death.

**
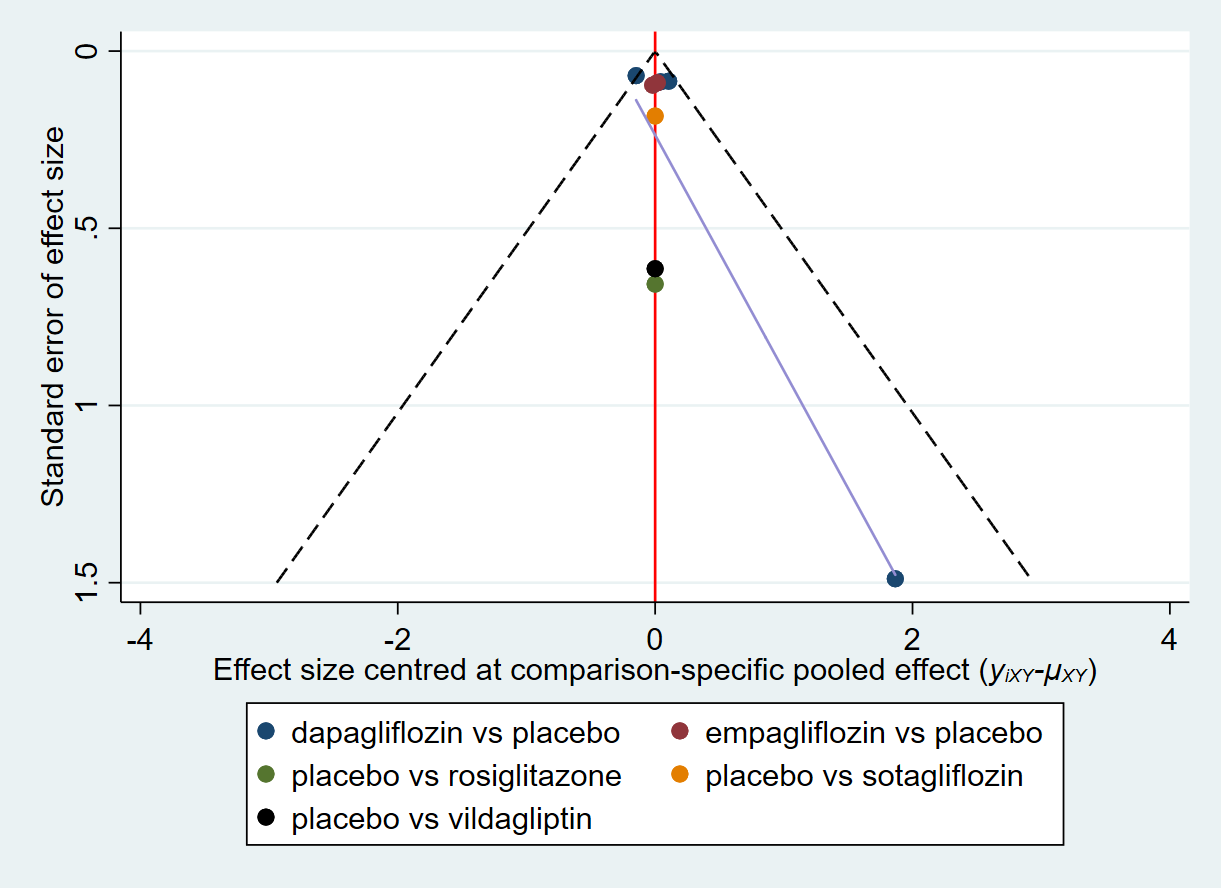
**

**Supplementary Fig. 35.** Publication bias of changes in follow-up more than one year patients' cardiovascular death.

**
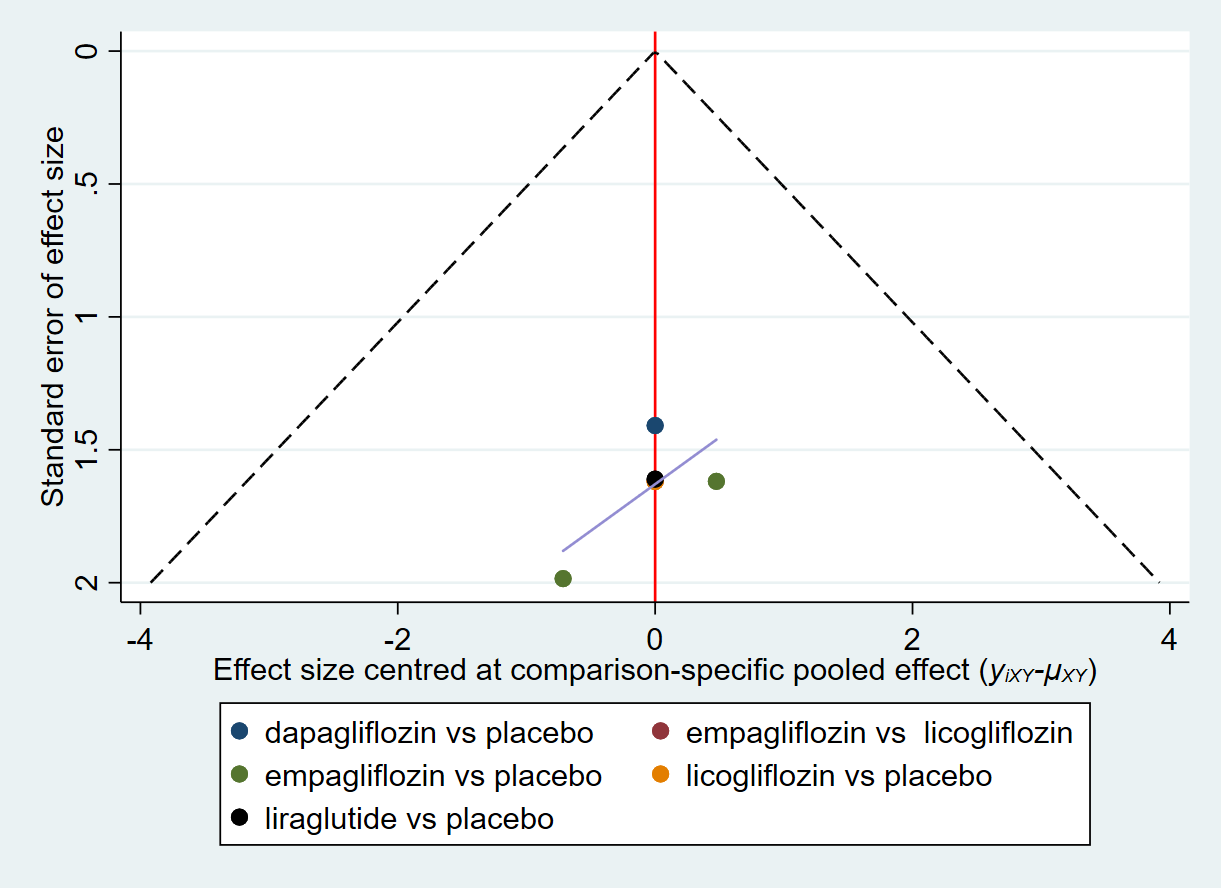
**

**Supplementary Fig. 36.** Publication bias of changes in follow-up less than one year patients' cardiovascular death.

**
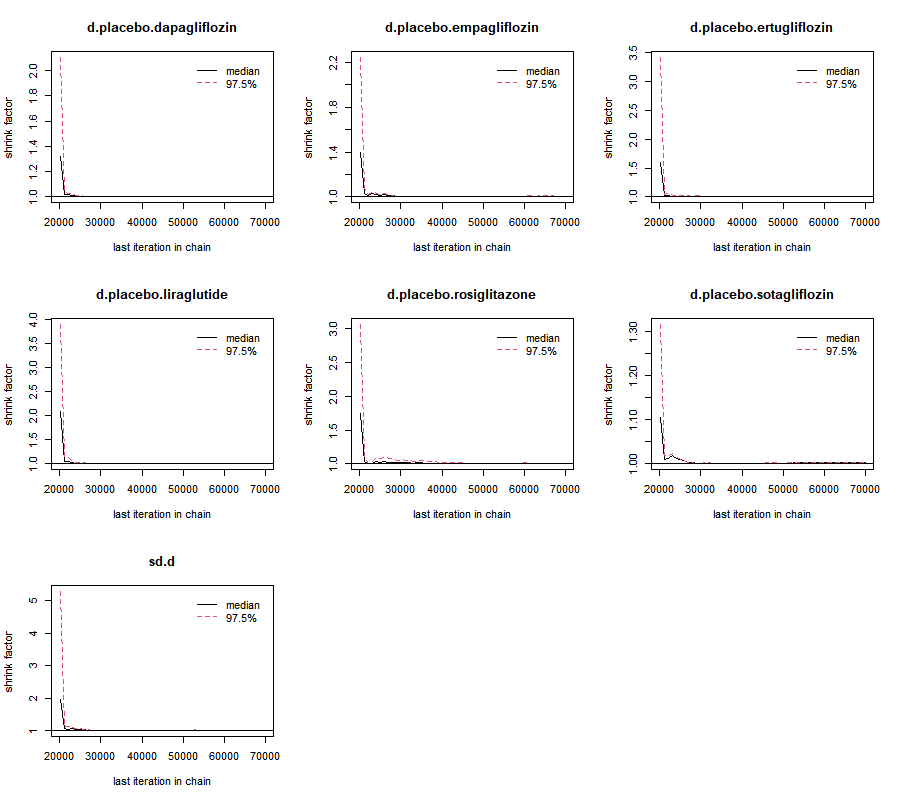
**

**Supplementary Fig. 37.** Diagnosis of convergence of readmission due to HF.

**
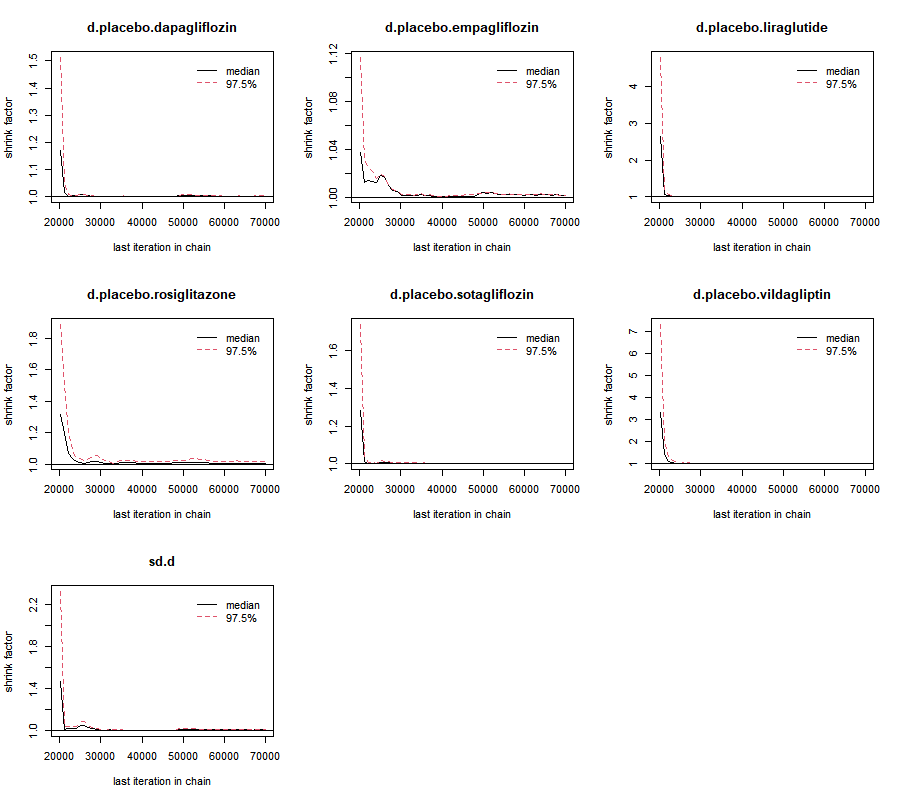
**

**Supplementary Fig. 38.** Diagnosis of convergence of all-cause death.

**
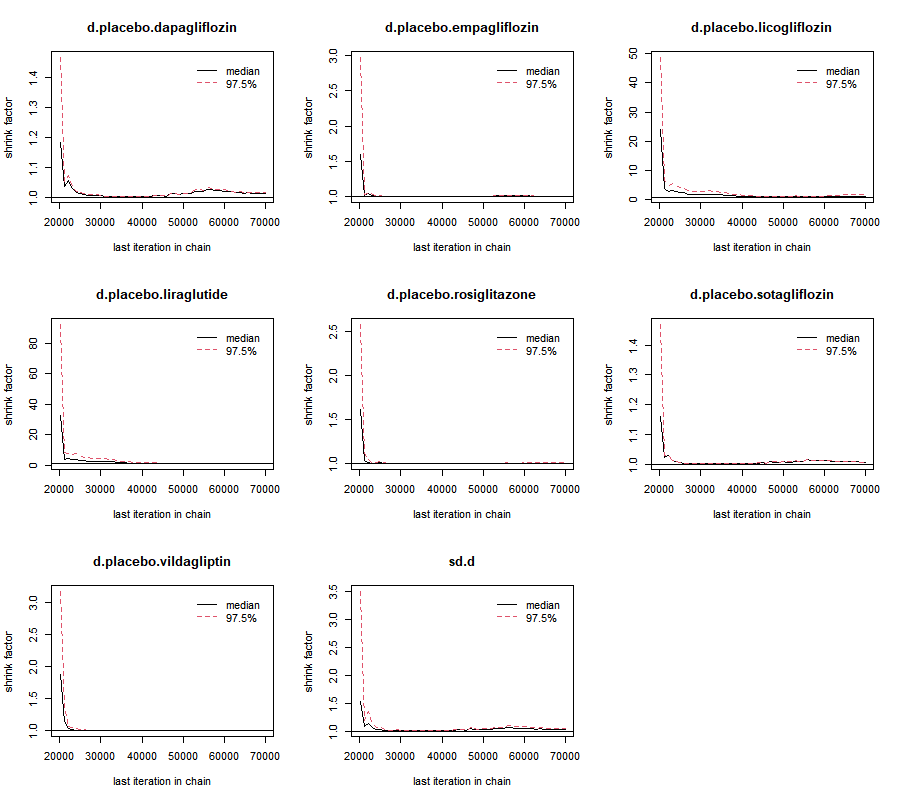
**

**Supplementary Fig. 39.** Diagnosis of convergence of cardiovascular death.

**
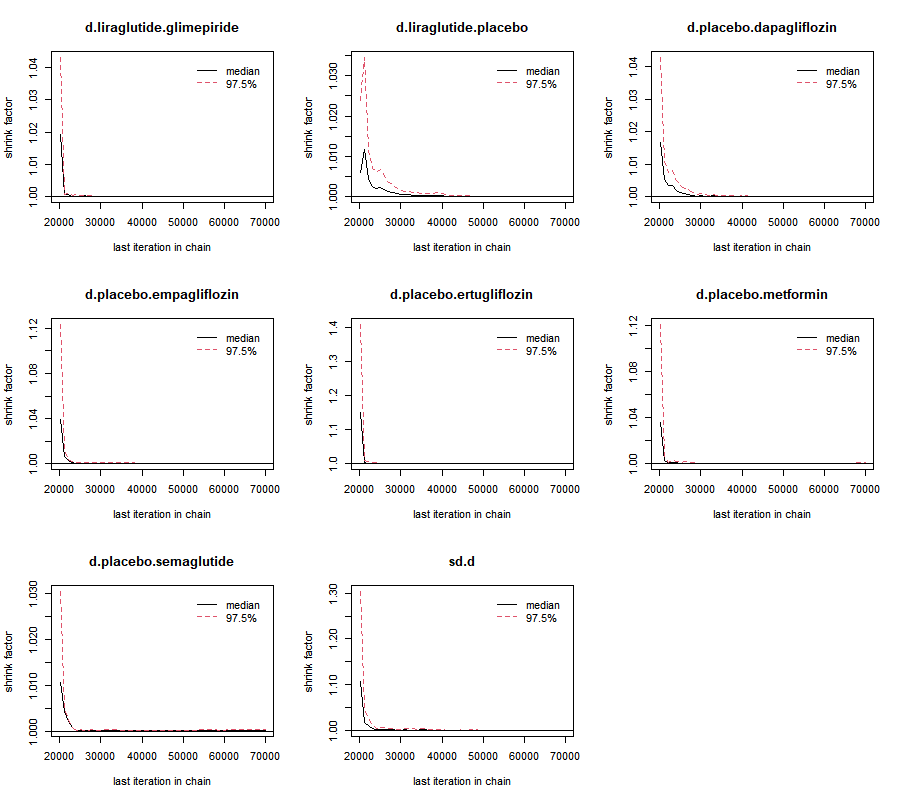
**

**Supplementary Fig. 40.** Diagnosis of convergence of NTpro-BNP.

**
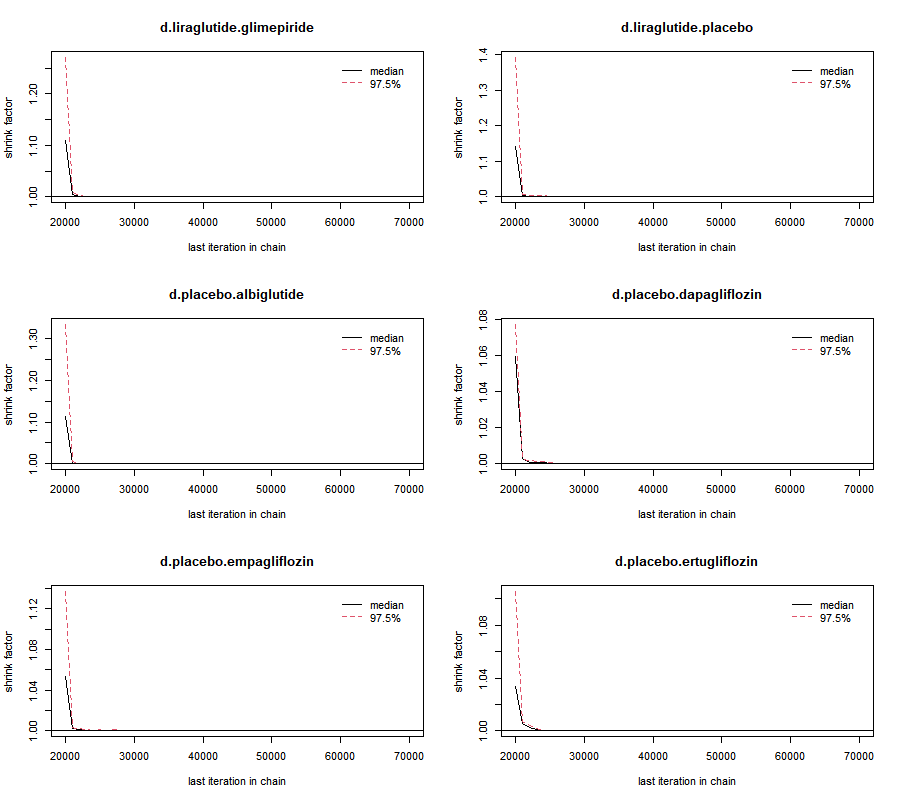
**

**Supplementary Fig. 41.** Diagnosis of convergence of LVEF.

**
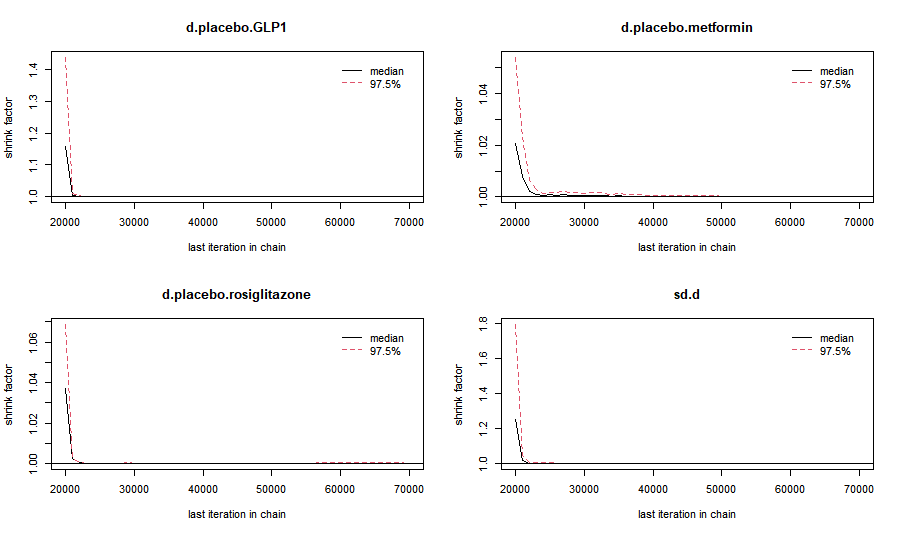
**

**Supplementary Fig. 42.** Diagnosis of convergence of LVEF 2.

**
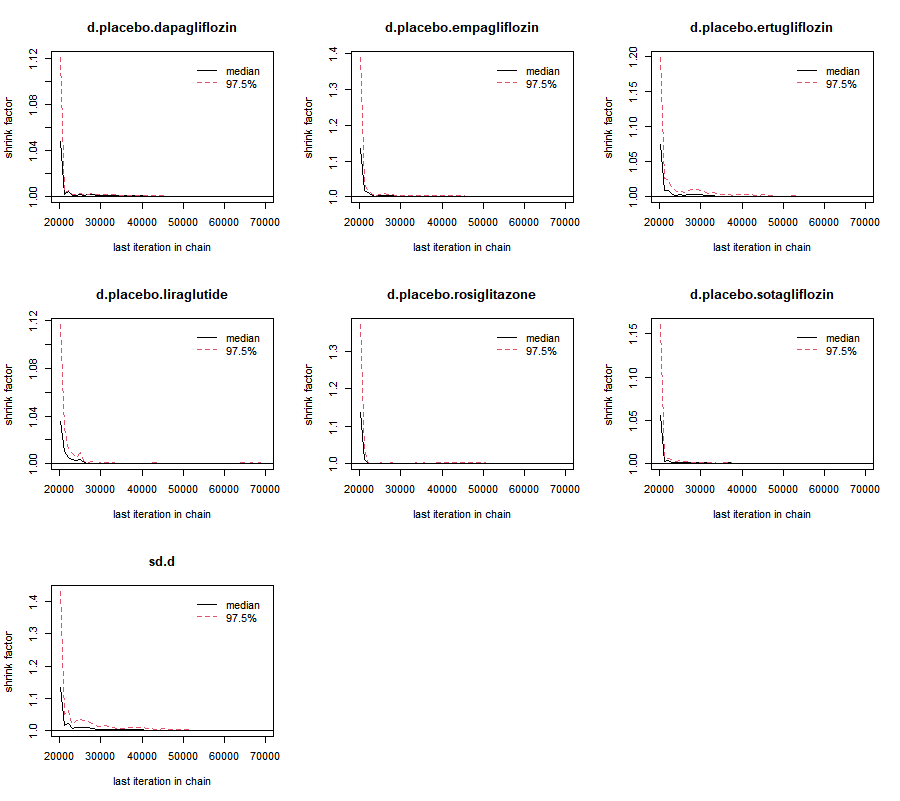
**

**Supplementary Fig. 43.** Diagnosis of convergence of HErEF patients' readmission due to HF.

**
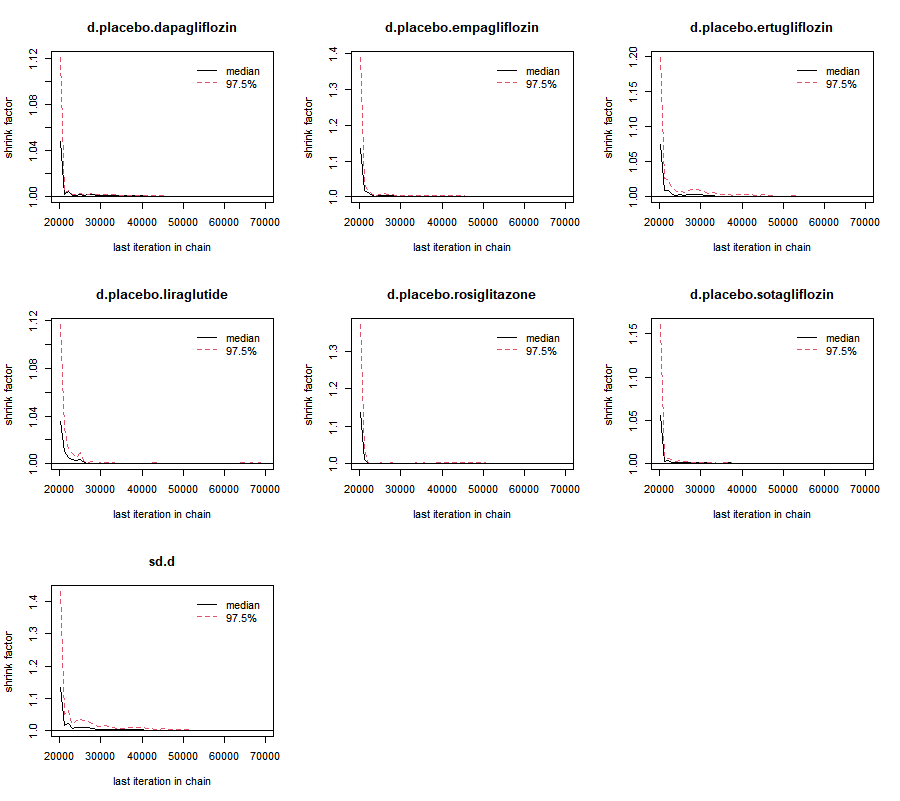
**

**Supplementary Fig. 44.** Diagnosis of convergence of HErEF patients' all-cause death.

**
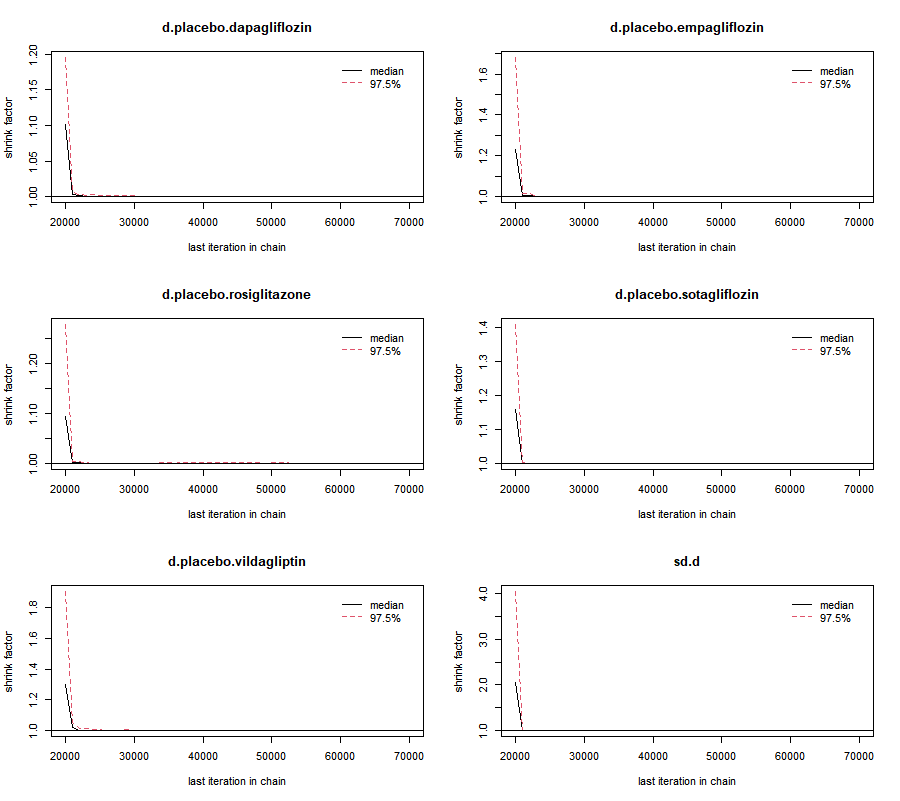
**

**Supplementary Fig. 45.** Diagnosis of convergence of HErEF patients' cardiovascular death.

**
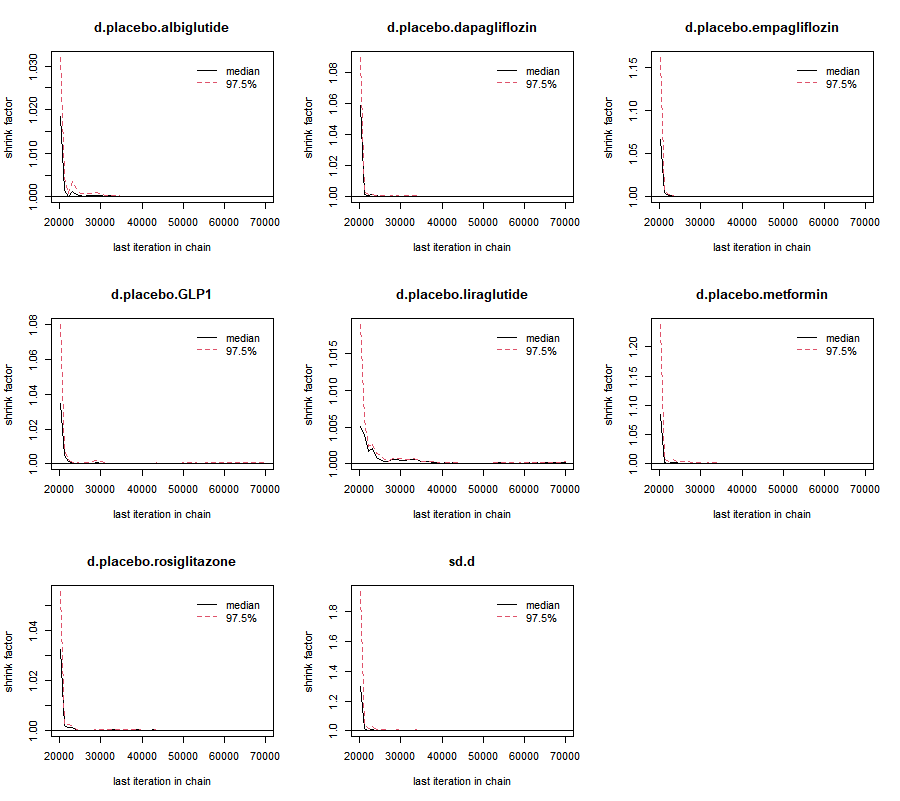
**

**Supplementary Fig. 46.** Diagnosis of convergence of HErEF patients' LVEF.

**
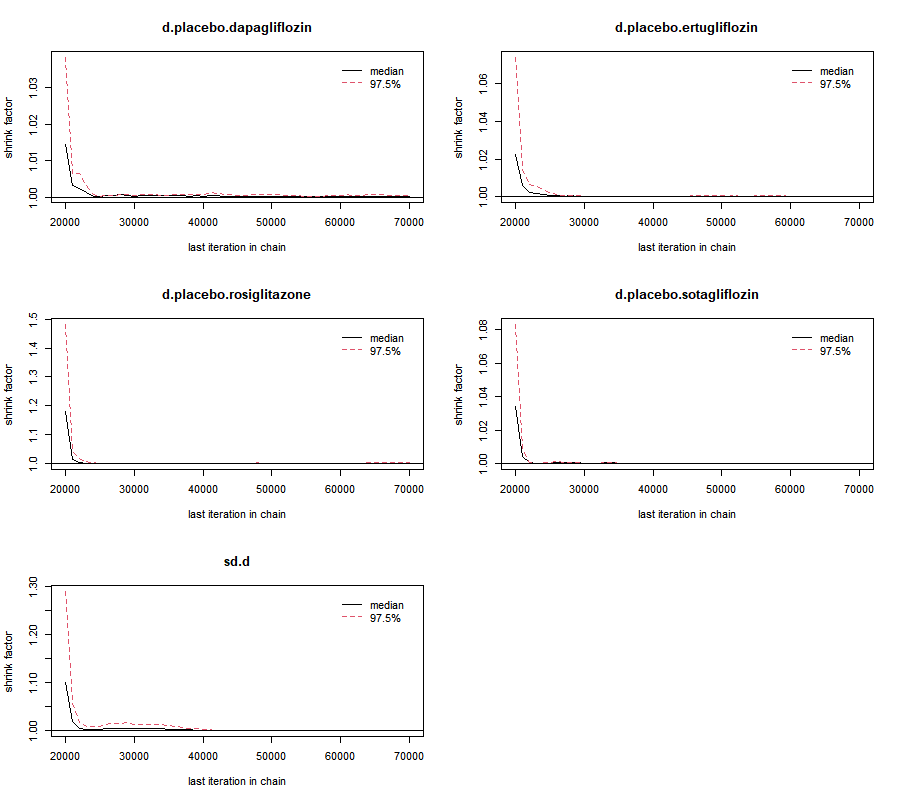
**

**Supplementary Fig. 47.** Diagnosis of convergence readmission due to HF in HF patients with T2DM.

**
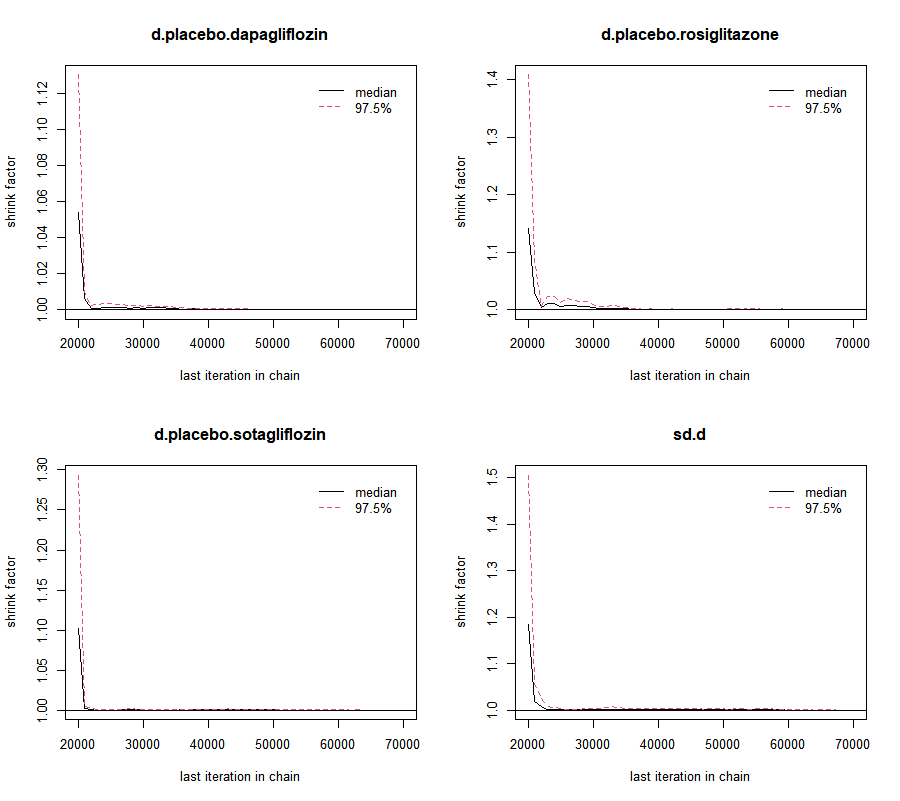
**

**Supplementary Fig. 48.** Diagnosis of convergence of all-cause death in HF patients with T2DM.

**
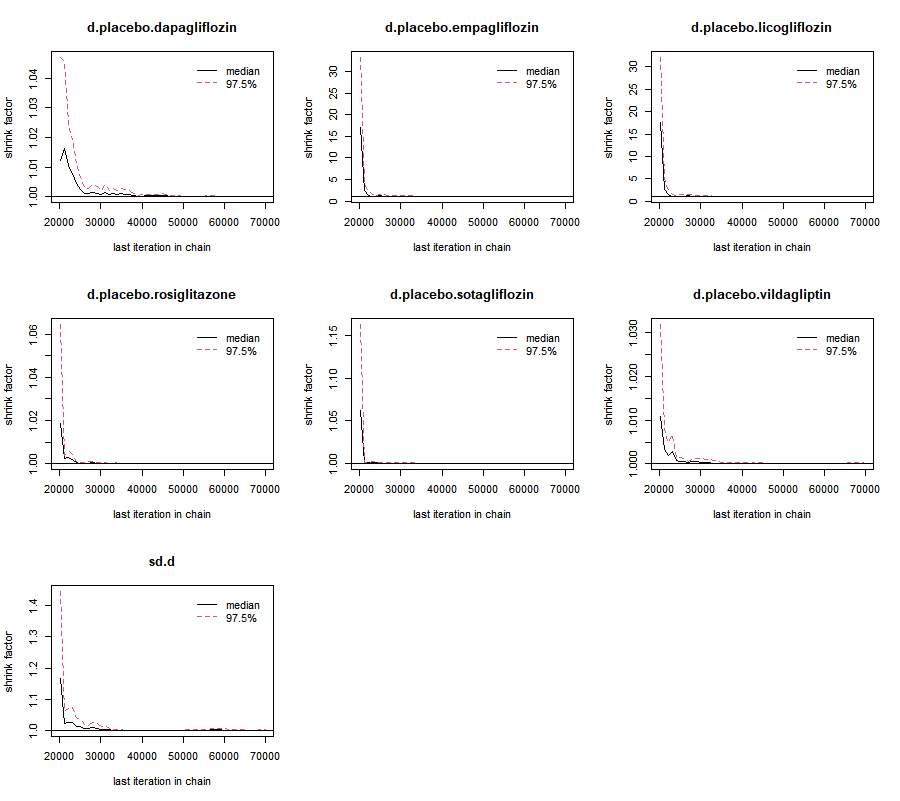
**

**Supplementary Fig. 49.** Diagnosis of convergence of cardiovascular death in HF patients with T2DM.

**
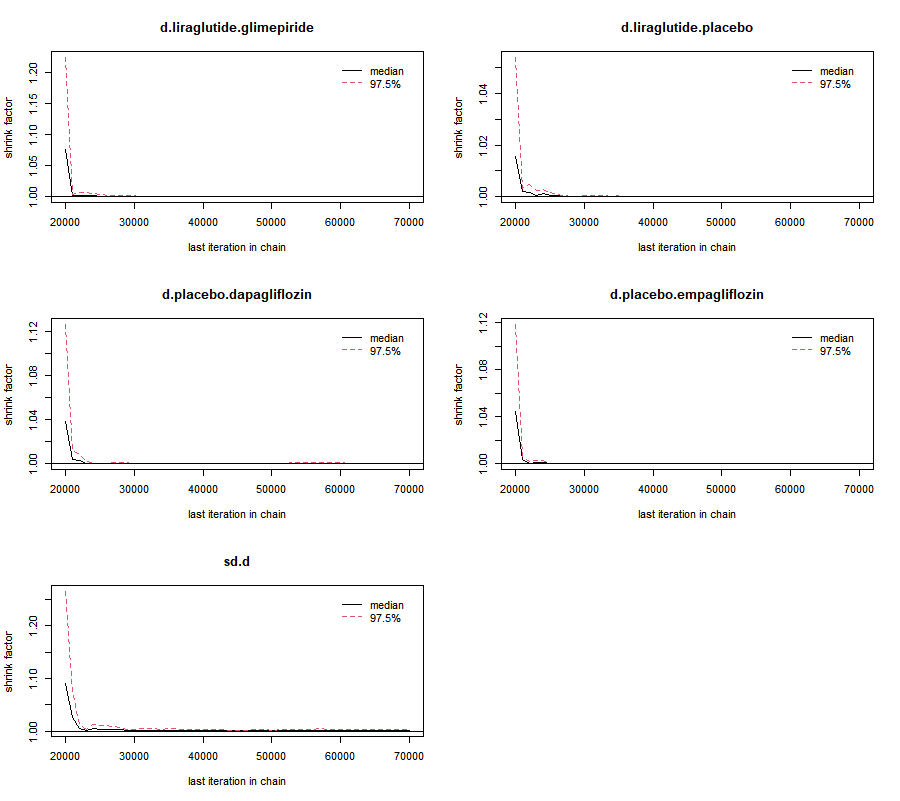
**

**Supplementary Fig. 50.** Diagnosis of convergence of changes in LVEF in HF patients without T2DM.

**
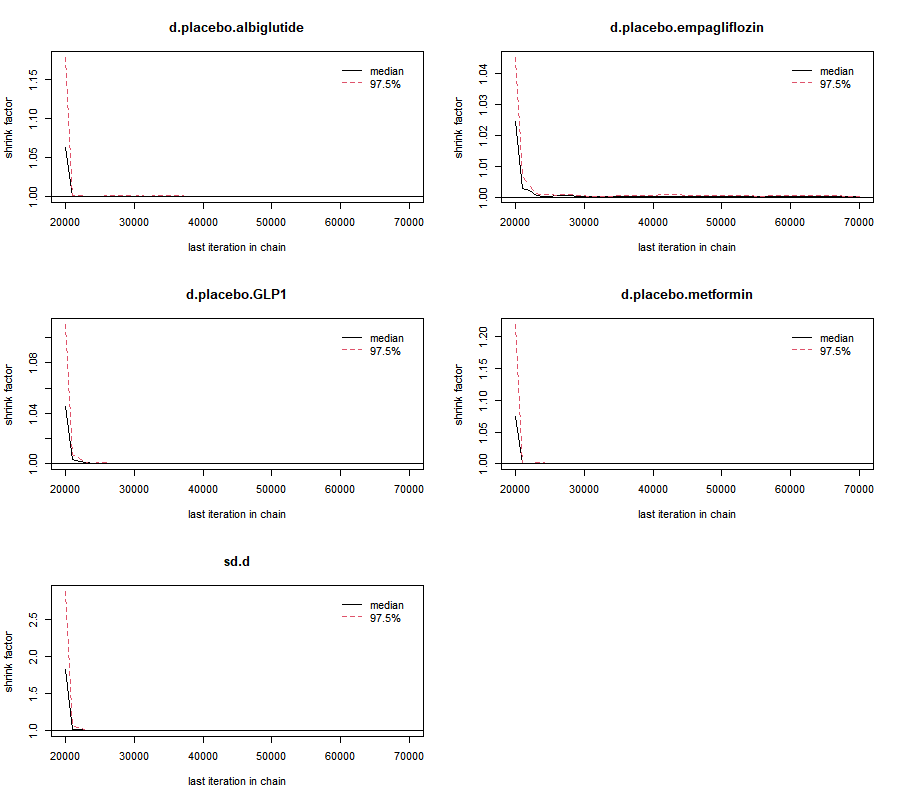
**

**Supplementary Fig. 51.** Diagnosis of convergence of changes in follow-up more than one year patients' readmission due to HF.


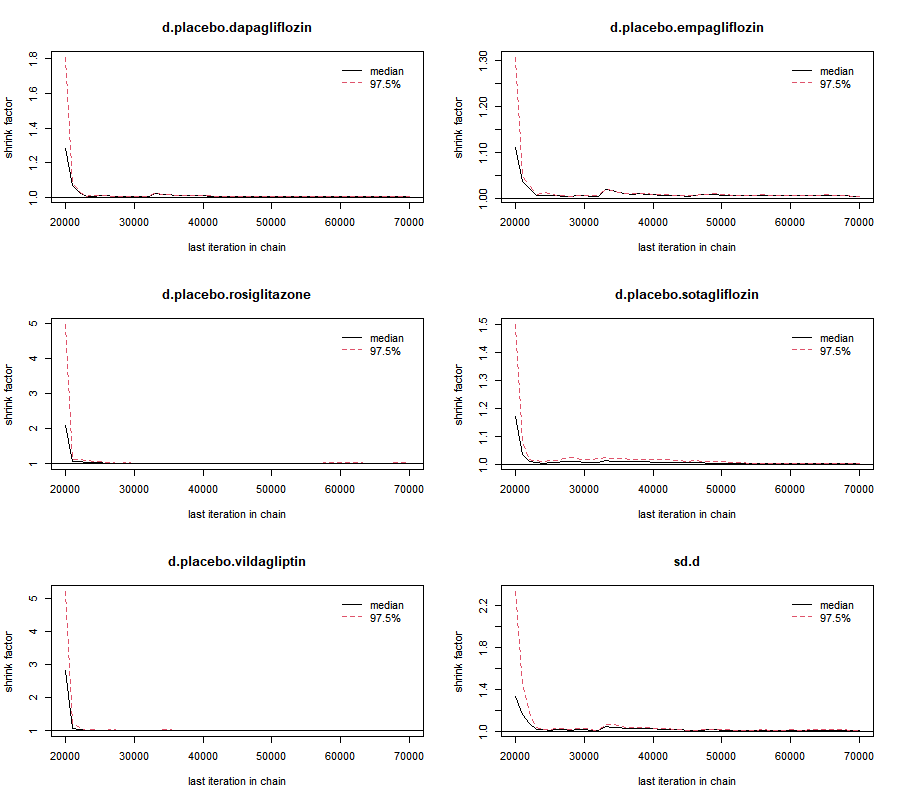


**Supplementary Fig. 52.** Diagnosis of convergence in follow-up more than one year patients' all-cause death.


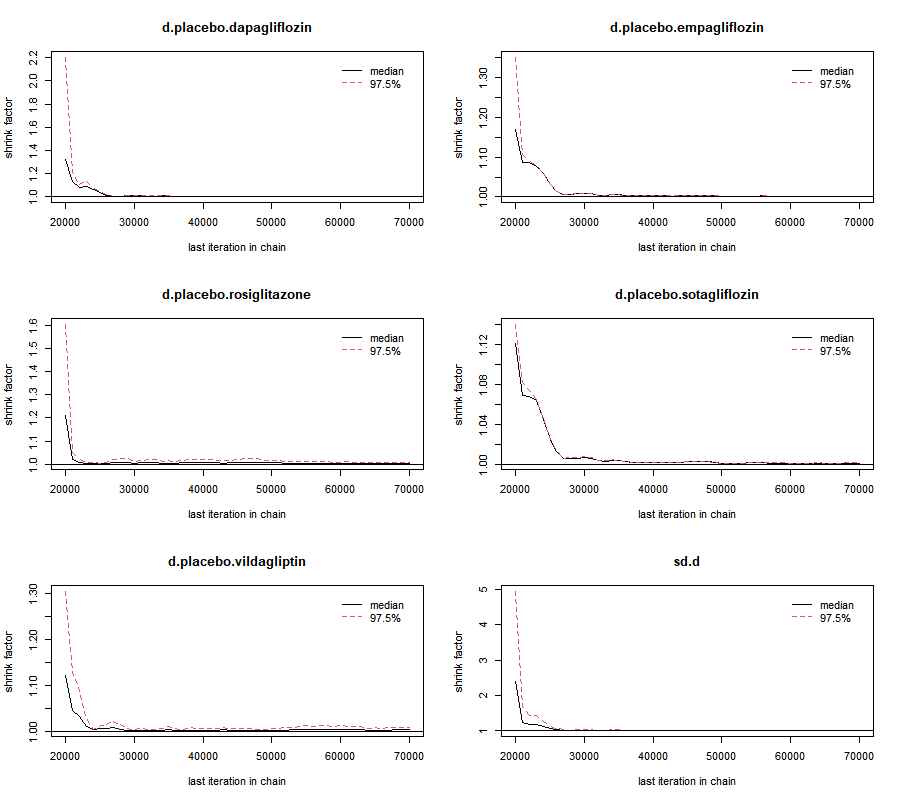


**Supplementary Fig. 53.** Diagnosis of convergence in follow-up more than one year patients' cardiovascular death.


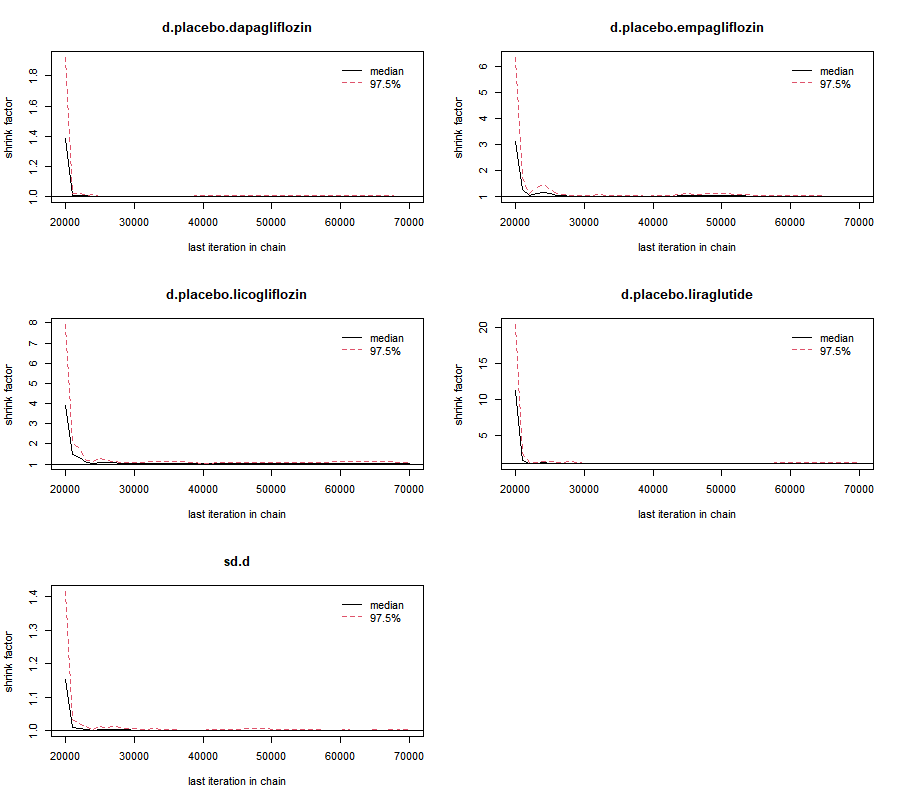


**Supplementary Fig. 54.** Diagnosis of convergence of changes in follow-up less than one year patients' cardiovascular death.


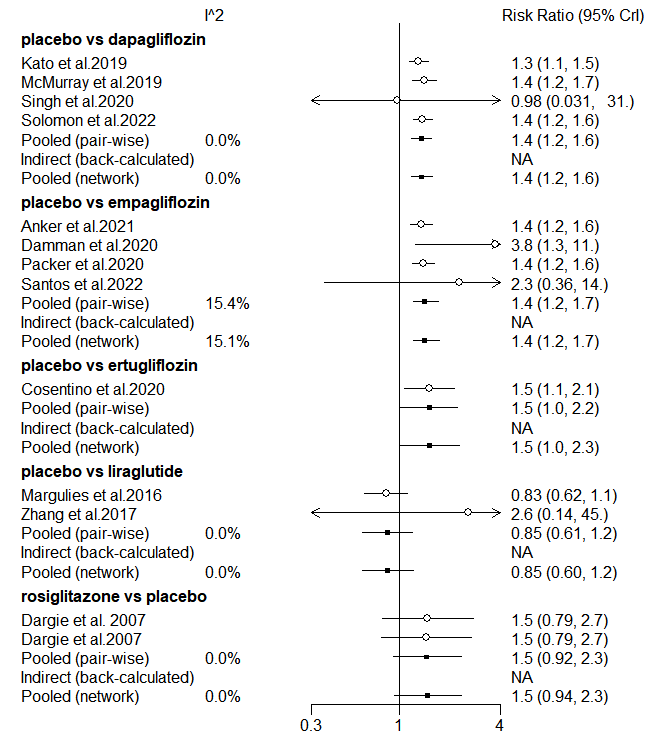


**Supplementary Fig. 55.** heterogeneity analysis of readmission due to HF.


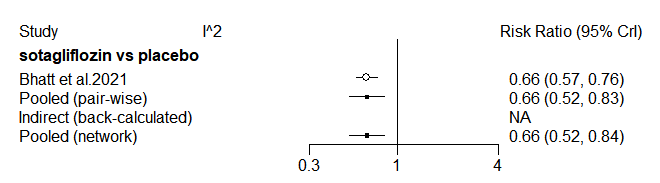


**Supplementary Fig. 56.** heterogeneity analysis of readmission due to HF 2.


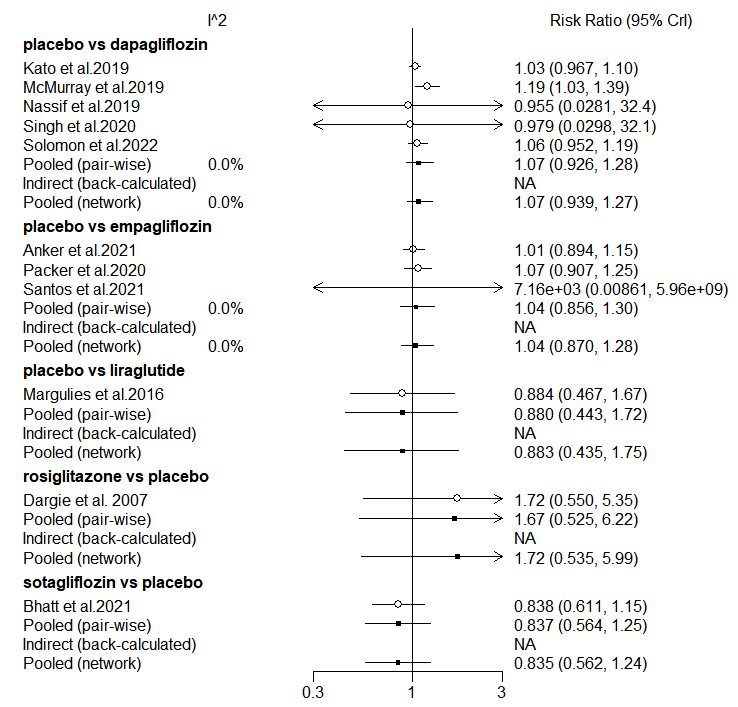


**Supplementary Fig. 57.** heterogeneity analysis of all-cause death.


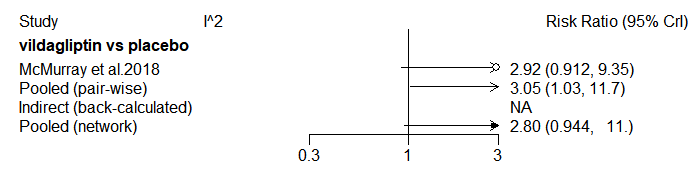


**Supplementary Fig. 58.** heterogeneity analysis of all-cause death 2.


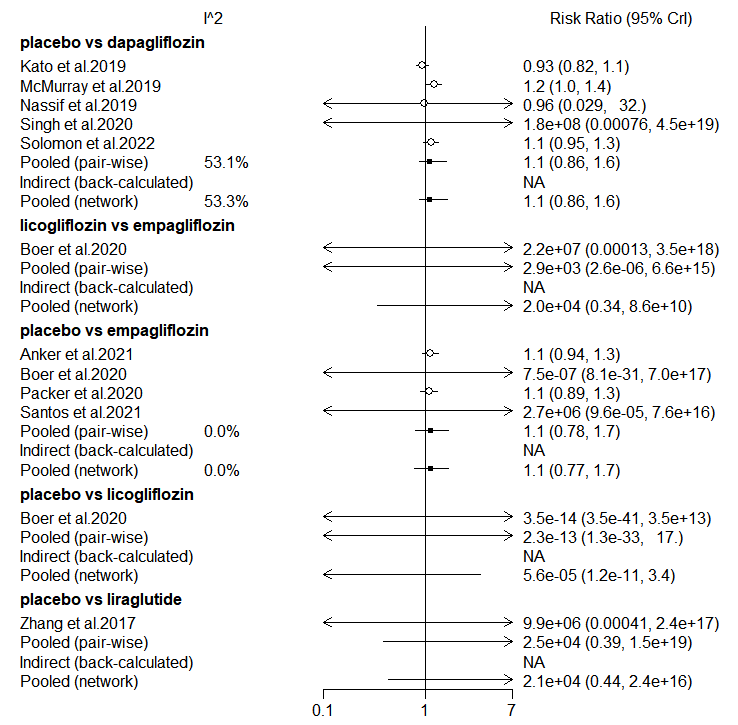


**Supplementary Fig. 59.** heterogeneity analysis of cardiovascular death.


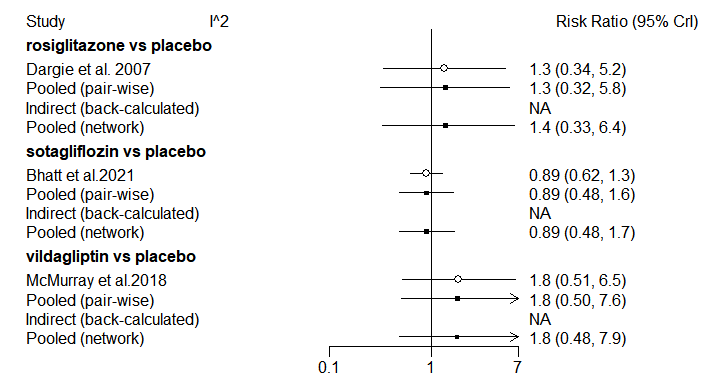


**Supplementary Fig. 60.** heterogeneity analysis of cardiovascular death 2.


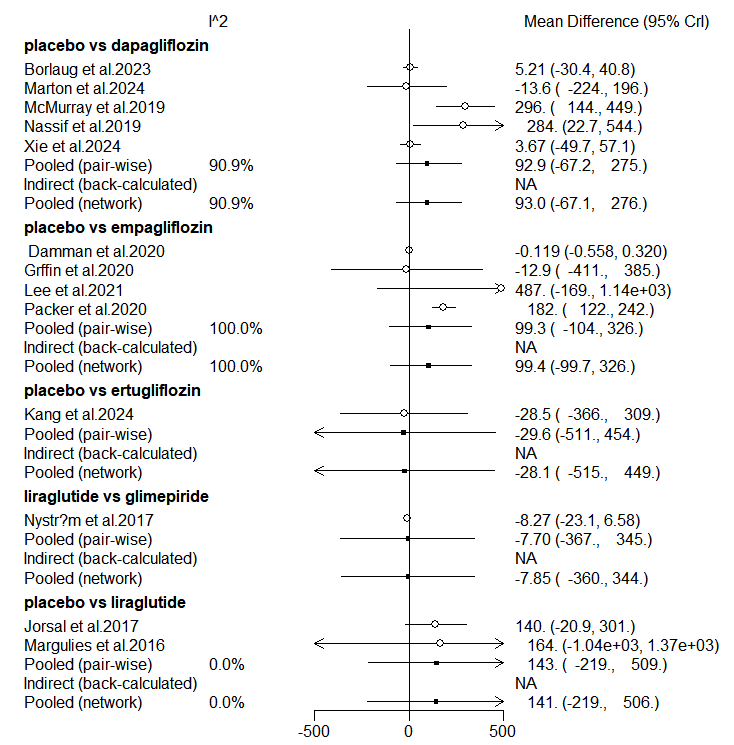


**Supplementary Fig. 61.** heterogeneity analysis of NTpro-BNP.


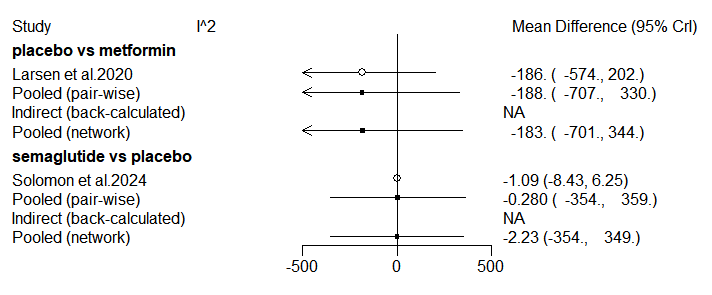


**Supplementary Fig. 62.** heterogeneity analysis of NTpro-BNP 2.


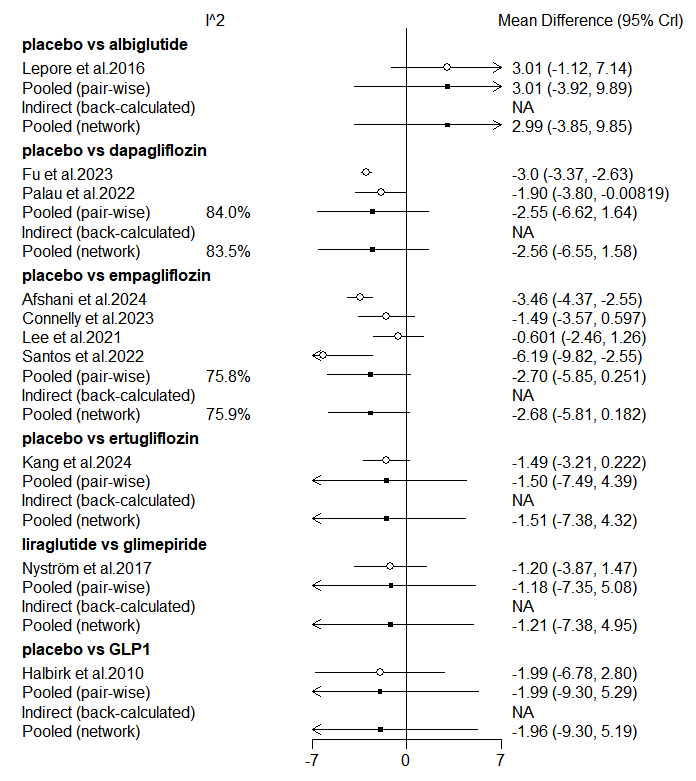


**Supplementary Fig. 63.** heterogeneity analysis of LVEF.


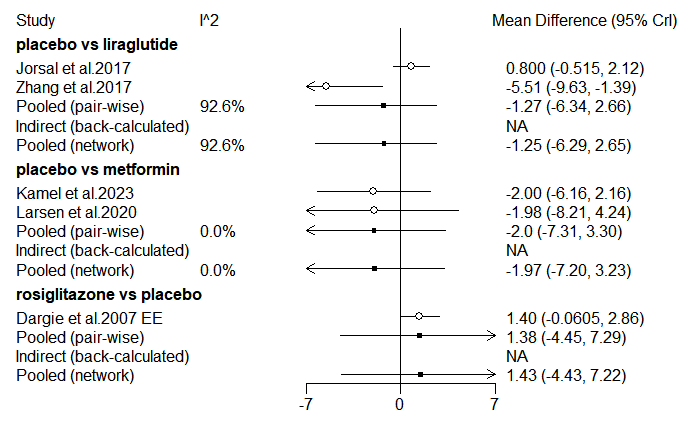


**Supplementary Fig. 64.** heterogeneity analysis of LVEF 2.


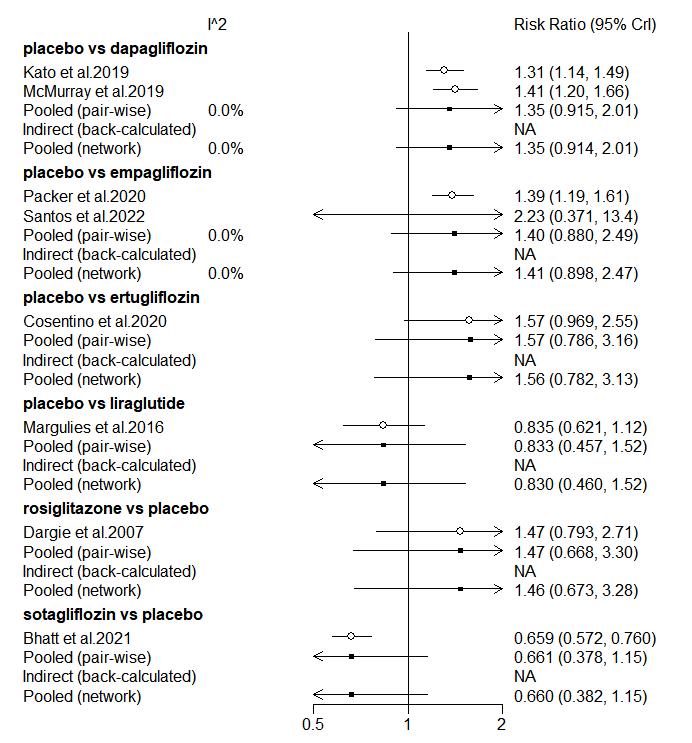


**Supplementary Fig. 65.** heterogeneity analysis of HErEF patients' readmission due to HF.


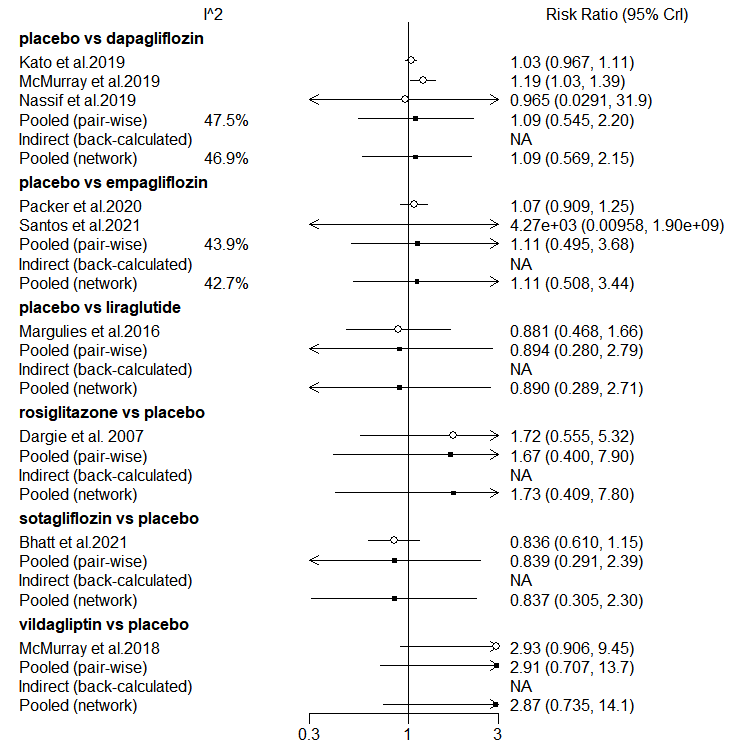


**Supplementary Fig. 66.** heterogeneity analysis of HErEF patients' all-cause death.


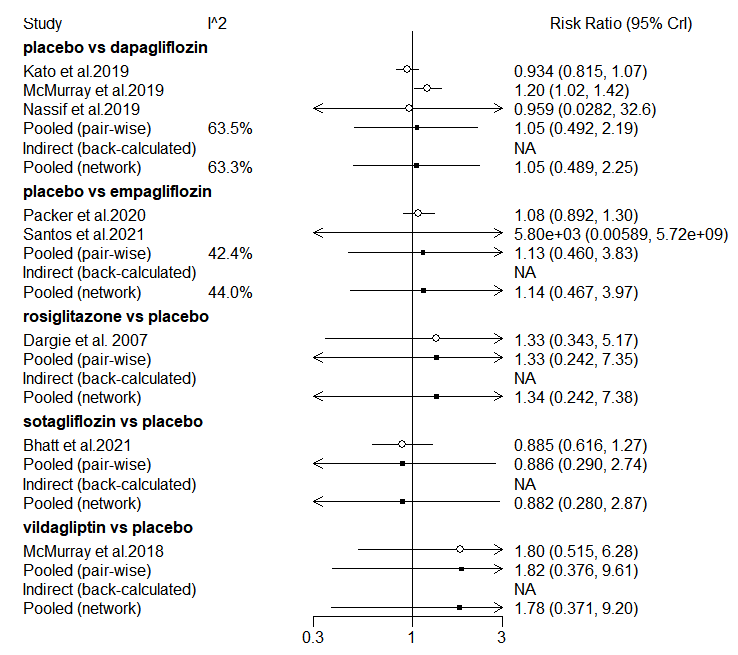


**Supplementary Fig. 67.** heterogeneity analysis of HErEF patients' cardiovascular death.


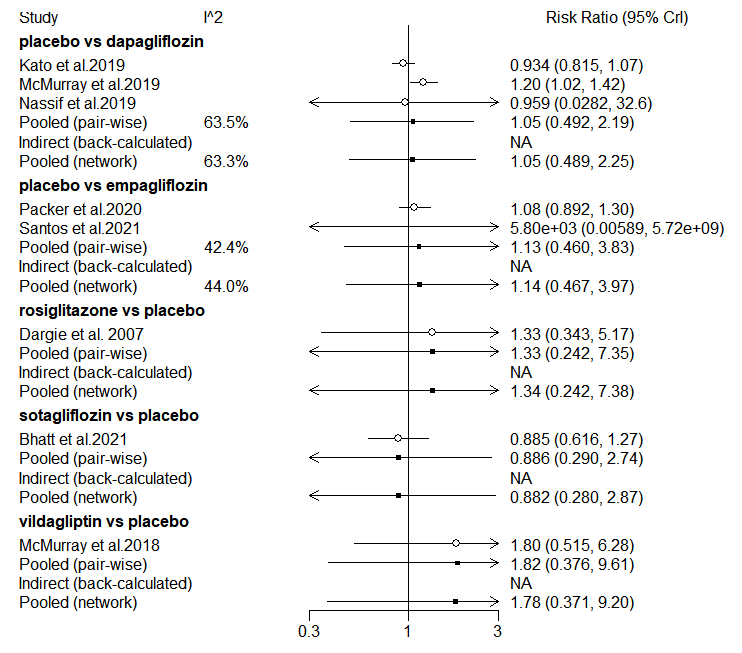


**Supplementary Fig. 68.** heterogeneity analysis of HErEF patients' LVEF.


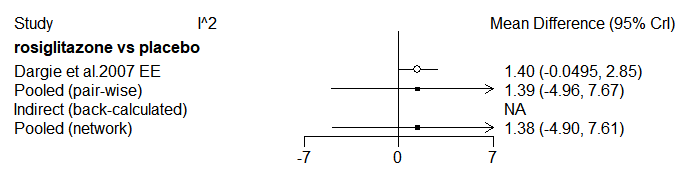


**Supplementary Fig. 69.** heterogeneity analysis of HErEF patients' LVEF 2.


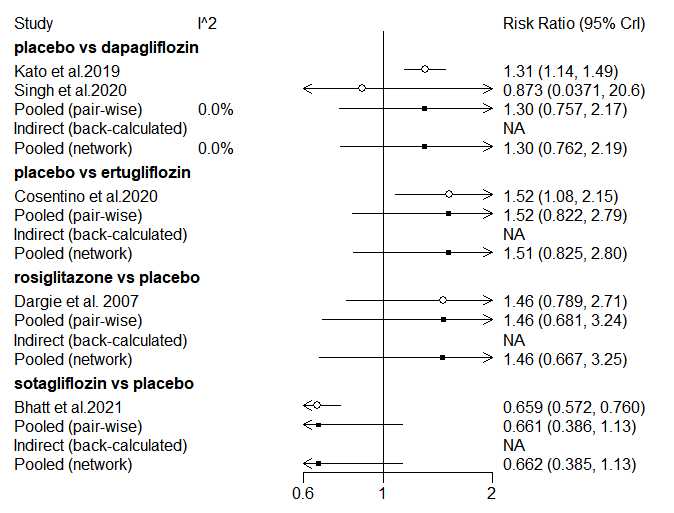


**Supplementary Fig. 70.** heterogeneity analysis readmission due to HF in HF patients with T2DM.

**
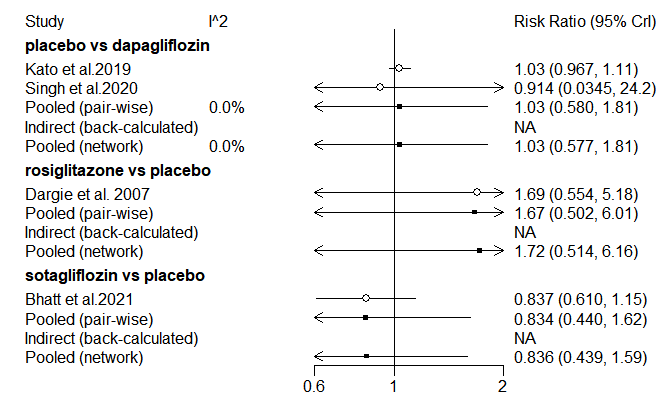
**

**Supplementary Fig. 71.** heterogeneity analysis of all-cause death in HF patients with T2DM.

**
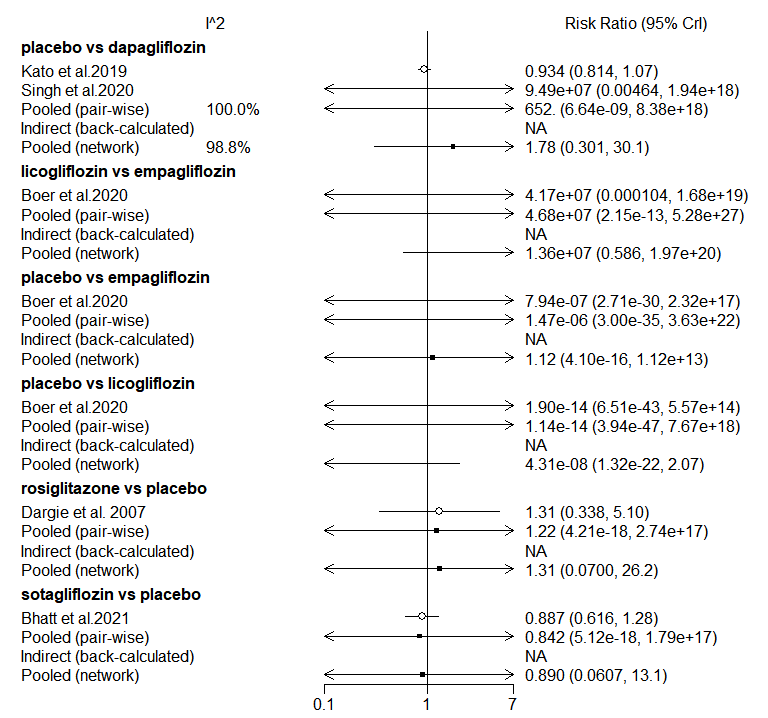
**

**Supplementary Fig. 72.** heterogeneity analysis of cardiovascular death in HF patients with T2DM.


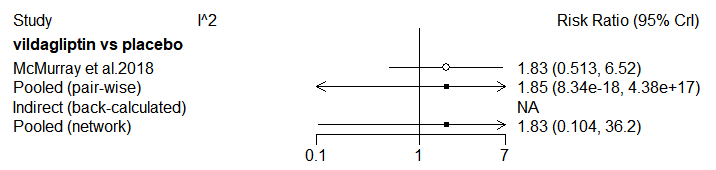


**Supplementary Fig. 73.** heterogeneity analysis of cardiovascular death in HF patients with T2DM.


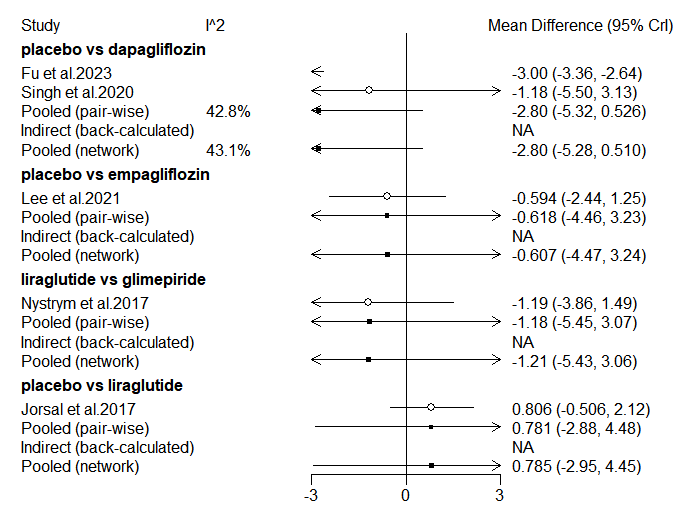


**Supplementary Fig. 74.** heterogeneity analysis of changes in LVEF in HF patients with T2DM.

**
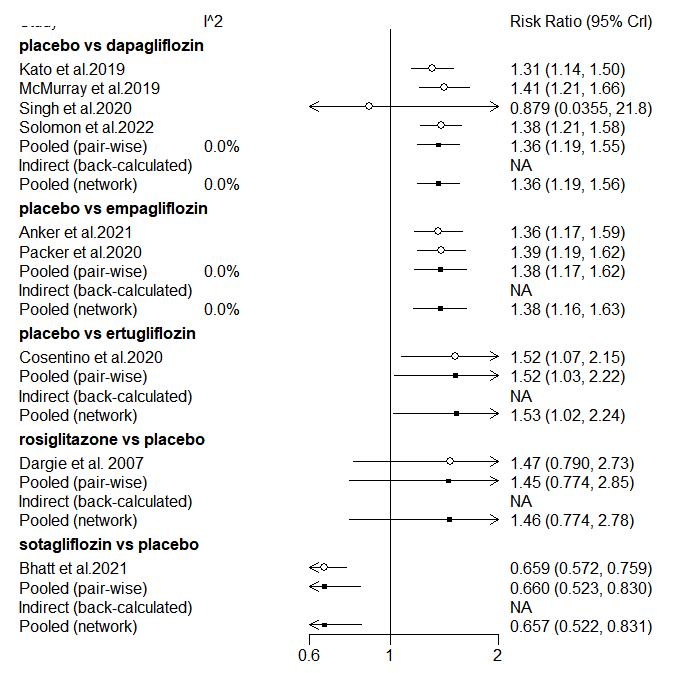
**

**Supplementary Fig. 75.** heterogeneity analysis of changes in follow-up more than one year patients' readmission due to HF.


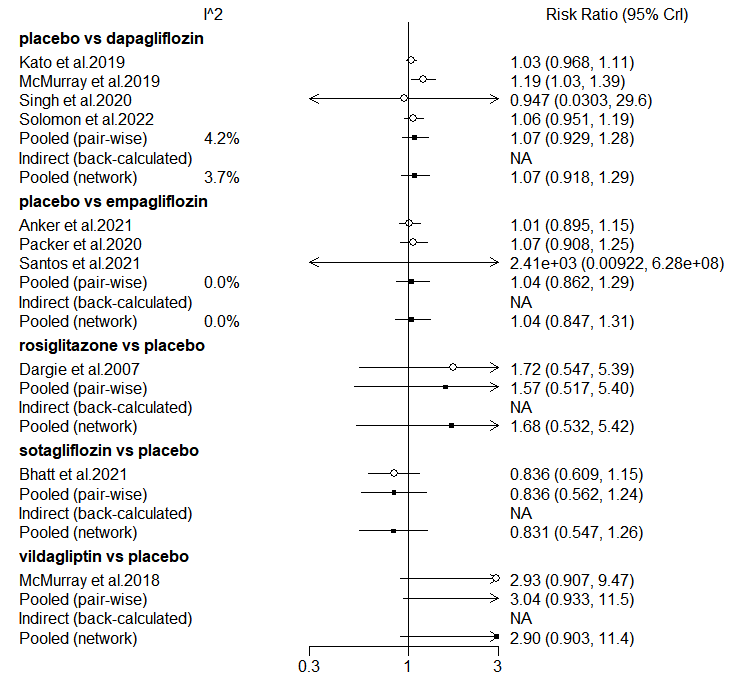


**Supplementary Fig. 76.** heterogeneity analysis in follow-up more than one year patients' all-cause death.


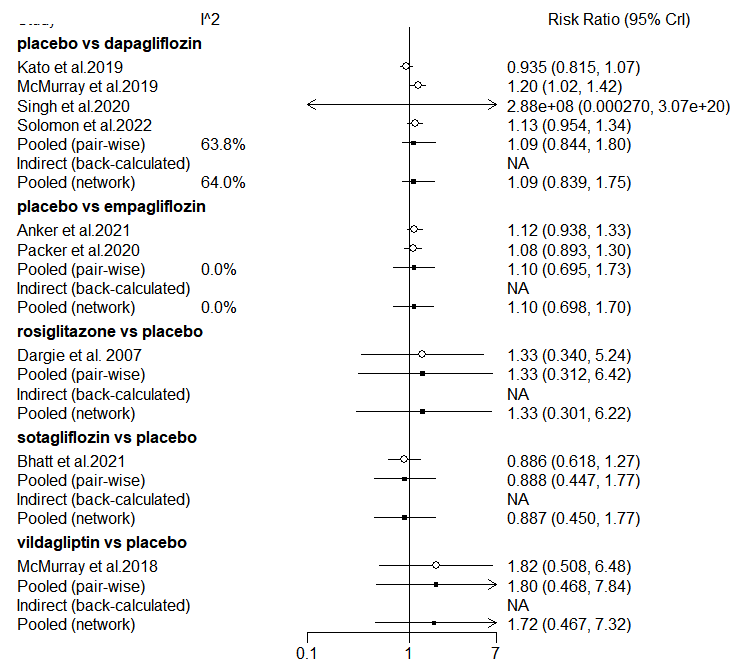


**Supplementary Fig. 77.** heterogeneity analysis in follow-up more than one year patients' cardiovascular death.


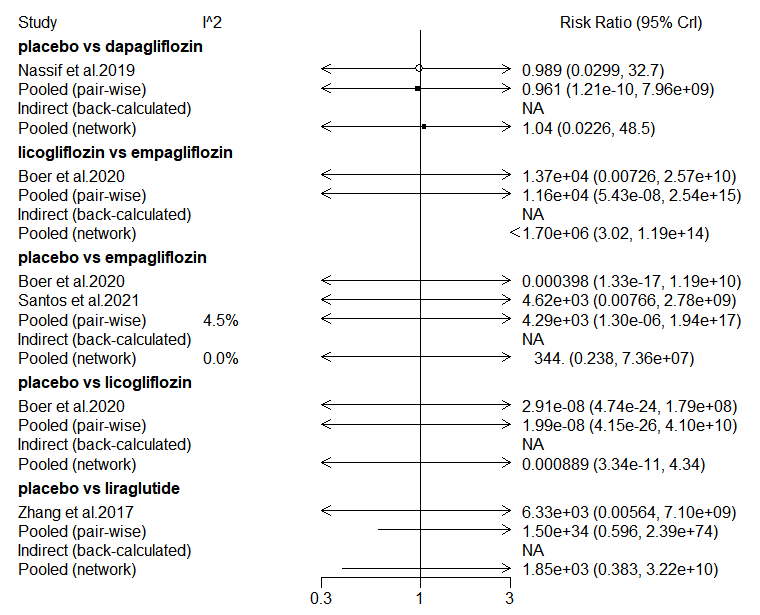


**Supplementary Fig. 78.** heterogeneity analysis of changes in follow-up less than one year patients' cardiovascular death.
